# Effects of changing ions on the crystal design, non-covalent interactions, antimicrobial activity, and molecular docking of Cu(II) complexes with a pyridoxal-hydrazone ligand

**DOI:** 10.3389/fchem.2024.1347370

**Published:** 2024-02-01

**Authors:** Claudia C. Gatto, Lucas M. Dias, Clarisse A. Paiva, Izabel C. R. da Silva, Daniel O. Freire, Renata P. I. Tormena, Érica C. M. Nascimento, João B. L. Martins

**Affiliations:** ^1^ Laboratory of Inorganic Synthesis and Crystallography, Institute of Chemistry, University of Brasilia, Brasília-DF, Brazil; ^2^ Graduate Program in Health Sciences and Technologies, Faculty UnB Ceilândia, University of Brasilia, Brasília-DF, Brazil; ^3^ Laboratory of Computational Chemistry, Institute of Chemistry, University of Brasilia, Brasília-DF, Brazil

**Keywords:** crystal structure, pyridoxal, Cu(II) complex, Hirshfeld surface, molecular docking, antibacterial activity

## Abstract

The present work reports the influence of the presence of different ions (Cl^−^, Br^−^, NO_3_^−^, or SO_4_^2−^) on the formation and proprieties of Cu(II) complexes with pyridoxal-benzoylhydrazone (PLBHZ). Four new complexes were successfully synthesized, [CuCl_2_(PLBHZ)] (1), [CuBr_2_(PLBHZ)] (2), [CuCl(PLBHZ)H_2_O]⋅NO_3_⋅H_2_O (3), and [CuSO_4_(PLBHZ)H_2_O]⋅3H_2_O (4), and characterized by spectroscopic and physicochemical methods. A single-crystal X-ray study reveals the Schiff base coordinated to the metal center tridentate by the *ONS*-donor system, resulting in distorted square pyramidal coordination geometries. Noncovalent interactions were investigated by 3D Hirshfeld surface analysis by the *d*_*norm*_ function, 2D fingerprint plots, and full interaction maps. The ion exchange is important in forming three-dimensional networks with π⋅⋅⋅π stacking interactions and intermolecular hydrogen bonds. The *in vitro* biological activity of the free ligand and metal complexes was evaluated against Gram-positive and Gram-negative bacterial strains and the free pyridoxal-hydrazone ligand showed higher activity than their Cu(II) complexes. Molecular docking was used to predict the inhibitory activity of the ligand and complexes against Gram-positive *Staphylococcus aureus* and Gram-negative *Escherichia coli* bacteria.

## 1 Introduction

B vitamins are essential for human metabolism and their lack can result in a wide range of health problems. Pyridoxal is among the six water-soluble vitamins of vitamin B_6_ that can be used for cell or bioimaging and anticancer applications (Gupta, 2022; Karthick et al., 2023). Pyridoxal and their metal complexes have been found useful in catalytic reactions and biological activities (Askarian et al., 2021; Ma et al., 2022; Dherbassy et al., 2023). The aldehyde group of pyridoxal can form through condensation reaction a variety of Schiff bases (Casas et al., 2012). These Schiff bases can offer diverse coordination possibilities with several transition metals (Casas et al., 2012; Askarian et al., 2021; Murašková et al., 2021; Poladian et al., 2021; Gamov et al., 2023). Their effectiveness is increased when coordinated with specific metals since the charges of the molecules are stabilized due to the binding of the metal ion with the ligand (Gatto et al., 2019; Gan et al., 2024). Pyridoxal-based ligands also exhibit antitumor activity, particularly those resulting from the reaction with semicarbazide or thiosemicarbazide (Qi et al., 2022; Jevtovic et al., 2023).

Hydrazones belong to a class of organic compounds that possess an azomethine–NHN=CR2 group showing wide application and biological properties due to their structural versatility and chelating capability (Sharma et al., 2020; Yan et al., 2024). Their wide popularity also is due to their straightforward synthesis from the condensation of hydrazides or hydrazines with aldehydes and ketones. Moreover, the attractiveness of hydrazone-based ligands is their stability and flexibility of coordination, evidenced in medicinal chemistry and enriched with pharmacological potential—including antibacterial, antifungal, antioxidant, and antitumor activity (Casas et al., 2012; Liu et al., 2022; Arora et al., 2023; Yuan et al., 2023). Pyridoxal–hydrazone compounds have many possible applications, such as promising candidates for the future development of antitubercular drugs (Shtyrlin et al., 2021), chemosensors for aluminum detection (Kejík et al., 2016), and iron-chelating agents for anticancer therapy (Sarno et al., 2018). Additionally, metal complexes with Schiff bases, particularly Cu(II) complexes, are important in the development of new compounds with applications such as coordination chemistry, industries, medicine, and biological activities (Soroceanu and Bargan, 2022; Boulechfar et al., 2023; Li et al., 2024). Studies have reported these complexesas having great antibacterial activity (Salehi et al., 2016; Fekri et al., 2019; Xu et al., 2020; Omidinia et al., 2022).

The antibiotic drug resistance generated in the treatment of bacterial infections in hospitals is critical in the cure of diseases caused by microorganisms (Marrero et al., 2006; Ziervogel and Roux, 2013). One of these resistances is the Gram-positive methicillin-resistant *Staphylococcus aureus* strain, which presents strong resistance to most beta-lactam antibiotics. This profile is determined for the biosynthesis of the penicillin-binding protein that molecularly decreases the affinity for beta-lactam drugs. The extracellular antibiotic-sensor domain of *S. aureus* (MecR1) is a serine protease-like enzyme responsible for the signal-transduction system related to antibiotic resistance. Once this enzymatic machinery is inhibited, the resistance of *S. aureus* to known antibiotic drugs is reduced (Marrero et al., 2006).

Another important pathway in antibiotic resistance for Gram-negative *Escherichia coli* and *Salmonella typhimurium* is the reduction of the permeability of their outer membrane (Ziervogel and Roux, 2013). This membrane has a barrier function that selects outside harmful agents, molecules, and compounds. *E. coli* produce porin channels that are responsible for cell diffusion (OmpF). When this porin channel develops mutations in key residues, the biological response is the modification of the cellular antibiotic levels which reduces their activity and inactivity. The development of new molecules for antibiotic therapy strategies is urgent and is vital for solving the problem of bacteria resistance to known antibiotic drugs (Ziervogel and Roux, 2013).

Due to our structural and biological interests in the study of new metal complexes based on hydrazone (Santiago et al., 2020; Santiago et al., 2022), we here study the reactions and resulting crystal structures between pyridoxal-benzoylhydrazone ligand and different Cu(II) salts. The compounds were analyzed by single-crystal X-ray diffraction, mass spectrometry, FT-IR, UV-Vis, and molecular docking. The influence of ion exchange on the structure and noncovalent interactions was investigated by 3D Hirshfeld surface analysis using the *d*_*norm*_ function, 2D fingerprint plots, and full interaction maps. Furthermore, biological activity was evaluated against Gram-positive *S. aureus* and Gram-negative *E. coli* bacterial strains. The results of this study of antibacterial activity show an interesting comparison between the free ligand and the Cu(II) complexes with the changing ions.

## 2 Materials and methods

### 2.1 Materials, methods, and instruments

All reagents and solvents used were obtained from commercial sources (Merck) and used as received. Elemental analyses were performed with a Perkin Elmer/Series II 2400 analyzer. Melting point was determined using the Micro Chemistry MQAPF-302 digital instrument. For comparison purposes, a flame atomic absorption spectrometer (AAS), Model PSD 120, operating with an acetylene/air flame, was used to determine the Cu(II) content in each complex. The lamp used was of the multi-element type Co/Cr/Cu/Fe/Mn/Ni, and each complex sample was made at a concentration of 3 mg L^−1^. Infrared spectra in ATR were recorded from KBr pellets (4,000–400 cm^−1^) using FT-IR Varian 640. A UV-Vis-NIR Varian Cary 5000 spectrophotometer was used, and the concentration used for all analyses was 2 × 10^−5^ M in methanol (MeOH) and N, N-dimethylformamide (DMF). ^1^H NMR and ^13^C NMR analyses were conducted using an NMR YH 600 Oxford. The ESI-MS and ESI-MS/MS spectra were obtained from AB Sciex TripleTOF 5600+ spectrometer equipment in positive mode, 5,500 V, and 200°C. Electrospray ionization mass spectrometry (ESI-MS) analysis was performed on an AB Sciex Triple TOF 5600+ mass spectrometer in positive mode, with a voltage of 5500 V and source temperature of 200°C.

### 2.2 Synthesis of pyridoxal-benzoylhydrazone hydrochloride monohydrated (PLBHZ)

The synthesis of PLBHZ was based on the literature (Back et al., 2009). The mixture of 3 mmol of pyridoxal hydrochloride and 3 mmol of benzoylhydrazide with 15 mL of ethanol was refluxed and heated for 2 h. A yellow solid product was obtained and filtered off the mother solution. Yield: 89.10%. Melting point: 197°C–198 °C. Elemental analysis calculated for C_15_H_16_ClN_3_O_3_: C, 55.99%; H, 5.01%; N, 13.06%. Found: C, 55.49%; H, 5.16%; N, 12.86%. Selected IR bands (KBr, ν/cm^−1^): ν(N–H) 2936, ν(O–H) 3349, ν(C=O) 1669, ν(C=N) 1589, ν(N–N) 1053. UV-Vis (MeOH): λmax = 285 nm, 306 nm, and 341 nm; RMN ^1^H (DMSO-d_6_ δ, ppm): 2.63 (s, 3H, CH_3_); 4.78 (s, 2H, CH_2_); 7.60 [t, 3J = 7.48 Hz, 2H, –CH (Gupta, 2022) Ar]; 7.69 [t, 3J = 7.48 Hz, H, –CH (Karthick et al., 2023) Ar]; 8.05 [d, 3J = 7.65 Hz, 2H, –CH (Ma et al., 2022) Ar]; 8.22 [s, H, –CH (Poladian et al., 2021) Ar]; 9.06 (s, H, –CH); 13.13 (s, H, –CH). ^13^C NMR (DMSO-d_6_ δ, ppm): 15 (CH_3_), 58 (–CH_2_), 126 (C_0_), 128.46 (–CH), 129 (–CH), 130 (–CH), 131 (–CH), 133 (–C_0_), 136.87 (–C_0_), 144 (–CH), 153 (–C_0_), 163 (–C_0_). ESI-MS [M+H]^+^ (calcd, found, m/z): 286.1192, 286.1192, 150.0522.

### 2.3 Synthesis of [CuCl_2_(PLBHZ)] (1)

The complex **(1)** was obtained by reaction of 0.1 mmol (32 mg) of PLBHZ and 0.1 mmol (17 mg) of CuCl_2_·2H_2_O. The compounds were solubilized in 10 mL of MeOH, and the mixture was kept under heat and reflux for 2 h. The final green solution was left at room temperature for the slow evaporation of the solvent. After 2 weeks, green solution suitable for single crystal X-ray analysis was obtained directly from the mother solution. Yield: 54% (23 mg). Elemental analysis calculated for C_15_H_15_Cl_2_CuN_3_O_3_: C, 48.46%; H, 4.07%; N, 11.30%. Found: C, 48.71%; H, 3.97%; N, 12.95%. Cu content (% proposed/FAAS ± sd): 15.14 ± 0/13.32 ± 0.0095. Selected IR bands (KBr, ν/cm^−1^): ν(O–H) 3131 cm^−1^, ν(C=O) 1594 cm^−1^, ν(C=N) 1560 cm^−1^, ν(C–O) 1305 cm^−1^, ν(N–N) cm^−1^. UV-Vis (MeOH): λmax = 326 nm, 341 nm, and 411 nm. UV-vis (DMF): λmax = 331 nm, 348 nm, and 415 nm. ESI-MS [M+H]^+^ (calcd, found, m/z): 347.0323, 347.0331.

### 2.4 Synthesis of [CuBr_2_(PLBHZ)] (2)

Complex **(2)** was obtained by reaction of 0.1 mmol (32 mg) of PLBHZ and 0.1 mmol (22 mg) of CuBr_2_. The compounds were solubilized in 10 mL of MeOH, and the mixture was kept under heat and reflux for 2 h. The green single crystals suitable for X-ray analysis were obtained directly from the mother solution with the slow evaporation of the solvent. Yield: 51% (26 mg). Elemental analyses calculated for C_15_H_15_Br_2_CuN_3_O_3_: C, 39.11%; H, 2.28%; N, 9.12%. Found: C, 39.36%; H, 2.31%; N, 9.48%. Cu content (% proposed/FAAS ± sd): 14.82 ± 0/15.63 ± 0.0125. FT-IR spectra selected bands (KBr, ν/cm^−1^): ν(O–H) 3360 cm^−1^, ν(C=O) 1594 cm^−1^, ν(C=N) 1546 cm^−1^, ν(C–O) 1308 cm^−1^, ν(N–N) 1036 cm^−1^. UV-Vis (MeOH): λmax = 326 nm, 341 nm, and 410 nm. UV-Vis (DMF): λmax = 330 nm, 348 nm, and 415 nm. ESI-MS [M+H]^+^ (calcd, found, m/z): 347.0331, 347.0330.

### 2.5 Synthesis of [CuCl(PLBHZ)H_2_O]⋅NO_3_⋅H_2_O (3)

Reactions were carried out by refluxing 0.1 mmol (32 mg) of PLBHZ previously dissolved in 5 mL of MeOH and 0.1 mmol (24 mg) of Cu(NO_3_)_2_·3H_2_O in 5 mL of MeOH for 2 h. Green crystals suitable for single-crystal X-ray diffraction were obtained after slow evaporation of the solvent. Yield: 52% (25 mg). Elemental analyses calculated for C_15_H_19_ClCuN_4_O_8_: C, 37.35%; H, 3.97%; N, 11.62%. Found: C, 37.15%; H, 3.67%; N, 11.75%. Cu content (% proposed/FAAS ± sd): 13.17 ± 0/13.05 ± 0.0067. FT-IR spectra selected bands (KBr, ν/cm^−1^): ν(O–H) 3254 cm^−1^, ν(C=O) 1589 cm^−1^, ν(C=N) 1554 cm^−1^, ν(C–O) 1311 cm^−1^, ν(N–N) 1022 cm^−1^. UV-Vis (MeOH): λmax = 327 nm, 342 nm, and 405 nm. UV-vis (DMF): λmax = 331 nm, 348 nm, and 413 nm. ESI-MS [M+H]^+^ (calcd, found, m/z): 347.0329, 347.0329.

### 2.6 Synthesis of [CuSO_4_(PLBHZ)H_2_O]⋅3H_2_O (4)

A solution of CuSO_4_·5H_2_O 0.1 mmol (25 mg) in MeOH (5 mL) was added to a stirred solution of PLBHZ (0.1 mmol, 32 mg) in MeOH (5 mL). The mixture was heated at reflux for 2 h. Dark green crystals suitable for X-ray diffraction were obtained directly from the mother liquor upon standing the solution at room temperature for several days. Yield: 50% (26 mg). Elemental analyses calculated for C_15_H_23_CuN_3_O_11_S: C, 37.15%; H, 4.78%; N, 8.67%. Found: C, 37.05%; H, 4.42%; N, 8.65%. Cu content (% proposed/FAAS ± sd): 12.29 ± 0/11.95 ± 0.0117. FT-IR spectra selected bands (KBr, ν/cm^−1^): ν(O–H) 3248 cm^−1^, ν(C=O) 1591 cm^−1^, ν(C=N) 1554 cm^−1^, ν(C–O) 1308 cm^−1^, ν(N–N) 1045 cm^−1^. UV-Vis (MeOH): λmax = 327 nm, 342 nm, and 406 nm. UV-Vis (DMF): λmax = 331 nm, 348 nm, and 415 nm. ESI-MS [M+H]^+^ (calcd, found, m/z): 347.0334, 347.0330.

### 2.7 Crystal structure determinations

X-ray diffraction data were collected at room temperature (296 K) on a Bruker CCD SMART APEX II single crystal diffractometer with Mo Kα radiation (0.71073 Å). The structures were solved using the Olex2 program (Dolomanov et al., 2009) directly with SHELXS (Sheldrick, 2008); subsequent Fourier-difference map analyses yielded the positions of the non-hydrogen atoms. The refinement was performed using SHELXL (Sheldrick, 2015). The empirical absorption corrections were applied by the SADABS program (Sheldrick, 1997). All refinements were made by full-matrix least-squares on F^2^ with anisotropic displacement parameters for all non-hydrogen atoms. Hydrogen atoms were included in the refinement in calculated positions, but those performing special bonds were located in the Fourier map. Molecular graphics were generated via Olex2 and MERCURY software (Dolomanov et al., 2009; Macrae et al., 2020). Crystal data, experimental details, and refinement results are summarized in Table 1.

**TABLE 1:** X-ray structure data collection and refinement parameters for complexes (1–4).

	(1)	(2)	(3)	(4)
Empirical formula	C_15_H_15_Cl_2_CuN_3_O_3_	C_15_H_15_Br_2_CuN_3_O_3_	C_15_H_19_ClCuN_4_O_8_	C_15_H_23_CuN_3_O_11_S
Formula weight (g mol^−1^)	419.74	508.66	482.33	516.96
Temperature/K	296 (2)	296 (2)	296 (2)	296 (2)
Crystal system	Monoclinic	Monoclinic	Monoclinic	Monoclinic
Space group	*P2*_*1*_ */c*	*P2*_*1*_ */c*	*P2*_*1*_ */n*	*P2*_*1*_ */c*
a (Å)	7.643 (17)	7.812 (6)	9.514 (13)	7.578 (7)
b (Å)	18.955 (4)	19.072 (16)	15.720 (2)	16.872 (16)
c (Å)	12.926 (3)	12.912 (10)	12.825 (17)	16.665 (15)
β (°)	96.112 (5)	95.617 (2)	100.198 (2)	91.085 (2)
Volume (Å^3^)	1862.1 (7)	1914.5 (3)	1887.8 (4)	2130.1 (3)
Z	4	4	4	4
*ρ*_calcd_ (mg cm^−3^)	1.497	1.765	1.697	1.612
*μ* (mm^−1^)	1.477	5.332	1.352	1.186
F (000)	852	996	988	1,068
Reflections collected	42,873	43,653	43,261	48,277
Reflections independent/R_int_	3,440/0.099	3,520/0.076	3,485/0.063	3,921/0.070
Data/restraints/parameters	3,440/0/220	3,520/0/220	3,485/0/273	3,921/0/287
Max/min transmission	0.806/0.737	0.370/0.300	0.745/0.672	0.745/0.621
Final R indexes [I > 2σ(I)]	0.041/0.102	0.058/0.181	0.029/0.072	0.033/0.075
GooF	1.056	1.059	1.043	1.024
Largest diff.peak/hole (eÅ^–3^)	0.306/−0.327	1.38/−1.855	0.303/−0.346	0.296/−0.310
CCDC	2296191	2296192	2296193	2296194

The crystallographic space group is a set of geometrical symmetry operations that take a three-dimensional periodic object (i.e., the crystal) into itself according to the International Table of Crystallography.

### 2.8 Computational details

CrystalExplorer 21.5 software (Spackman et al., 2021) was used to calculate the Hirshfeld surfaces (HS) and related 2D fingerprint plots, using the crystallographic information files (CIFs) obtained by single-crystal X-ray. A fixed color scale of −0.4740 (red) to 2.6654 (blue) was used to map the 3D d_norm_ surfaces (normalized contact distances) for the ligands and complexes. A 2D fingerprint plot of d_i_ versus d_e_ was used to identify and quantify in percentages the intermolecular interactions of the compounds. The full interaction maps were produced using MERCURY software (Macrae et al., 2020). The interaction maps are indicative of hydrogen bond acceptors (red), donors (blue), and hydrophobic interactions (brown), respectively.

### 2.9 Biological activity

Antibacterial tests were performed by the microdilution broth method in 96-well microplates, in triplicate, using bacterial strains obtained from the American Type Culture Collection (ATCC): Gram-positive *S. aureus* ATCC 29213 and Gram-negative *E. coli* ATCC 25922 bacterial. Both strains were grown in Müller–Hinton Broth (MHB) at 37 °C overnight, resulting in approximately 5 × 10^8^ colony forming units (CFU) per mL according to the guidelines of the Clinical and Laboratory Standards Institute (CLSI) (Patel et al., 2015). The positive control was oxacillin for *S. aureus* at concentration 1 μg mL^−1^ and ampicillin at concentration 6 μg mL^−1^ for *E. coli*. Minimal inhibitory concentrations (MICs) of 0.1 and 99.9 were determined by the broth microdilution method, according to CLSI (Patel et al., 2015).

### 2.10 Molecular docking

Molecular docking is a computational chemistry tool used on a large-scale system to theoretically predict the most favorable orientation of the ligand when interacting with a given target protein. Molecular docking empirically determines the interaction energy and predicts the inhibition constant of the receptor–ligand complex (R–L). Knowing the energetically most favorable orientation of a ligand is an important step in predicting the binding affinity of the R–L system. The score functions are applied to determine the energies involved in the activation or inhibition process of a given biochemical target (Morris et al., 2009; Nascimento et al., 2017).

In this work, two rigid-flexible docking studies (receptor–ligand) were carried out to elucidate a possible interaction path between enzymatic bioreceptors of Gram-positive (*S. aureus*) and Gram-negative (*E. coli*) bacteria and the PLBHZ molecules, their metal complexes (1–4), and the classic inhibitors ampicillin (to *E. coli*) and oxacillin (to *S. aureus*). For the docking study, the AutoDockTools 4 package (Morris et al., 2009) was used with the AutoDock Vina docking algorithm (Trott and Olson, 2010). The enzymatic systems used to evaluate the effectiveness of inhibition of the PLBHZ ligand and their Cu(II) complexes were (a) the methicillin resistance protein MecR1, which is an extracellular penicillin-sensor domain of *S. aureus*, whose crystallographic structure is deposited in the Protein Data Bank under the code 2IWD (Marrero et al., 2006), complexed with oxacilloyl and with a resolution of 2.40 Å; (b) the outer membrane protein porin of *E. coli* (OmpF/A), with crystallographic structure deposited in the PDB under the code 4GCP, complexed with ampicillin at 1.98 Å resolution.

For the molecular docking protocol, the systems were prepared as follows:

i) 3D molecular structures: The bioreceptors were obtained by searching crystallographic data deposited in the PDB. The protein structures were extracted directly from the PDB under the codes 2IWD and 4GCP. Sequentially, only the residues of the protein structure were maintained, and the amino acid residues with the best crystallographic resolution were selected. The geometric structures of the classical inhibitors were extracted directly from the PDBs: oxacilloyl (PDB: 2IWD) and ampicillin (PDB: 4GCP). The crystallographic structures of the PLBHZ ligand and their Cu(II) complexes were obtained directly from the crystallographic data carried out in this work.
ii) Hydrogen atoms: Hydrogen atoms were added to proteins and small molecules using the standard AutoDock protocol.
iii) Addition of charges: For proteins, the Kollman-type charges were addressed; for ligands, Gasteiger charges were used; for both, the standard protocol of AutoDockTools was followed.
iv) Binding site setup: A grid box was built around the center of mass of each complexed classical inhibitor with the bioreceptor target. To study the structure of MecR1 (PDB: 2IWD), the dimensions of the grid box were 18.0 Å × 18.0 Å × 15.0 Å, with a spacing of 0.375 (points spacing). The box was centered at the point (20.622, 12.347, and 79.033). For the Ompf/A study (PDB: 4GCP), the dimensions of the grid box were 17.25 Å × 18.0 Å × 17.25 Å, with a spacing of 0.375 (points spacing) centered on the point (39.044, −26.679, and −18.162). These dimensions ensured that all ligands had an area of probable interactions suitable for their volume and the active site residues of the enzymes.
v) Docking run: To perform this study, the AutoDock Vina algorithm was applied to search for a maximum of 1,000 solutions at an exhaustiveness level of 32.
vi) The score obtained through the score function of the program relates the negative score value directly to the binding energy, which is given in kcal.mol^−1^. This value represents the highest interaction affinity between a given ligand, in the most appropriate conformation and the proteomic target of interest.
vii) For both enzymatic targets, redocking studies were performed to validate the proposed docking protocols above. For MecR1 and its oxacilloyl ligand, the value of the RMSD was 0.86 Å, and the binding energy (score) was −6.90 kcal mol^−1^. In the case of OmpF/A and its ampicillin ligand, the RMSD value was 0.36 Å and the binding energy (score) was −6.90 kcal mol^−1^. The values of the RMSD, under 2.0 Å for both redocking studies, validate our docking protocol properly.
viii) Hydrogen and van der Waals interactions are fundamental for carrying out biochemical processes. In these studies, the hydrogen interactions were considered with the maximum distance between a hydrogen atom and a carbon atom or heteroatoms of 3.4 Å. For medium- and long-range (van der Waals) interactions, such as π⋅⋅⋅π stacking, π-sigma, π-alkyl, and π-sulfur interactions, interactions with a maximum distance of 6.0 Å between the ligand were taken into account. To visualize the results and the main distances between the ligand atoms and the amino acid, residue of the active site Discovery Studio Visualizer software was used (Biovia and Release, 2020).

## 3 Results and discussion

**FIGURE 1:**
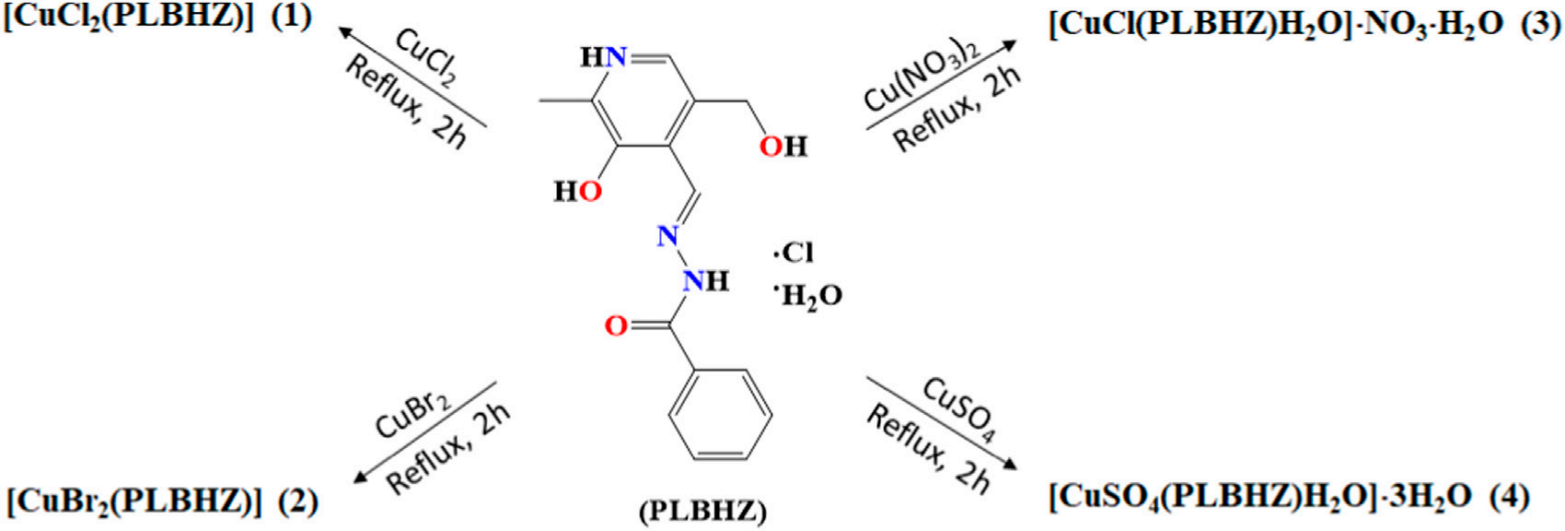
Summary of products obtained from the reactions between pyridoxal-benzoylhydrazone ligand (PLBHZ) and different Cu(II) salts.

Pyridoxal-benzohylhydrazone ligand (PLBHZ) was synthesized by the condensation reaction of pyridoxal hydrochloride with benzohylhydrazine. The reactions were carried out in ethanol with a catalytic amount of acetic acid to ensure the formation of the azomethine and the separation of the product from the mixture. Their Cu(II) complexes were obtained from the reactions between the PLBHZ ligand and different Cu(II) salts (Figure 1). The compounds were characterized by physicochemical and spectroscopic methods. The crystal structures of the complexes were established by single-crystal X-ray diffraction.

### 3.1 Structural analyses

**FIGURE 2:**
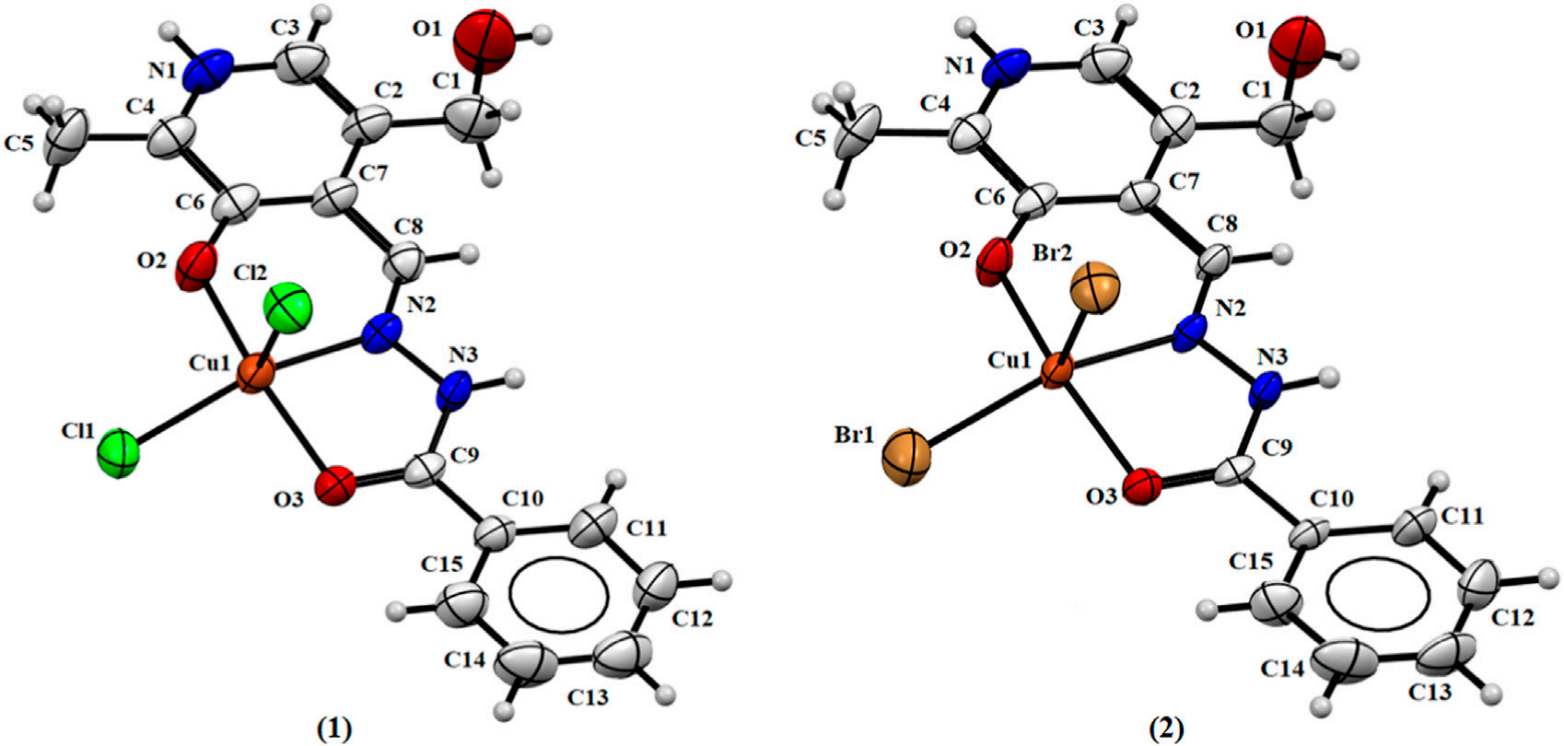
Molecular structure of (1) and (2) with crystallographic labeling (50% probability displacement).

The single-crystal structural analysis revealed isoelectronic structures of the Cu(II) complexes (1) and (2) (Figure 2). The metal centers are found in distorted square pyramidal environments and coordinated by the phenolic oxygen atom, the nitrogen atom of the azomethine group, and the oxygen atom of the ketone group of ligand molecules. Two chloride or bromide ions from the starting metal salt fulfill the coordination sphere. The bond length Cu-Cl of 2.238 (2) and 2.585 (2) Å in (1) and Cu-Br of 2.332 (2) and 2.699 (2) Å in (2) are in agreement with the range found for Cu(II) complexes (Vidovic et al., 2011; Gatto et al., 2019). The apical bond copper–halogen is longer than the axial bond due to the repulsion along the *z-*axis with the presence of two electrons in the *dz* (Gupta, 2022) orbitals. The data show that the ligand was coordinated to the Cu(II) atom by the isomer *E* and keto tautomer, with bond lengths C9-O3 of 1.259 (4) Å and 1.235 (9) Å and C9-N3 of 1.340 (4) Å and 1.351 (9) Å, respective of (1) and (2).

Addison’s parameter (τ_5_), where τ_5_ = (β-α)/60 (α and β are the largest coordination angles) was used to predict the pentacoordinate geometry, where the value of τ = 0 is for a perfect square pyramid geometry and τ = 1 is for a perfect trigonal bipyramid geometry (Addison et al., 1984). The calculated Addison’s parameters for complexes (1) and (2) were calculated with the angles O2-Cu1-O3 and N2-Cu1-X1 (where X = Cl and Br to (1) and (2), respectively), with the values of 167.46 (1)° and 161.75 (9)° in complex (1) and 167.3 (2)° and 161.64 (2)° in complex (2). The found τ values were 0.095 and 0.094 for (1) and (2), respectively, suggesting a distorted square-based pyramid for the metal ions, with a chloride or bromide ion in the apical position of the pyramid.

**FIGURE 3:**
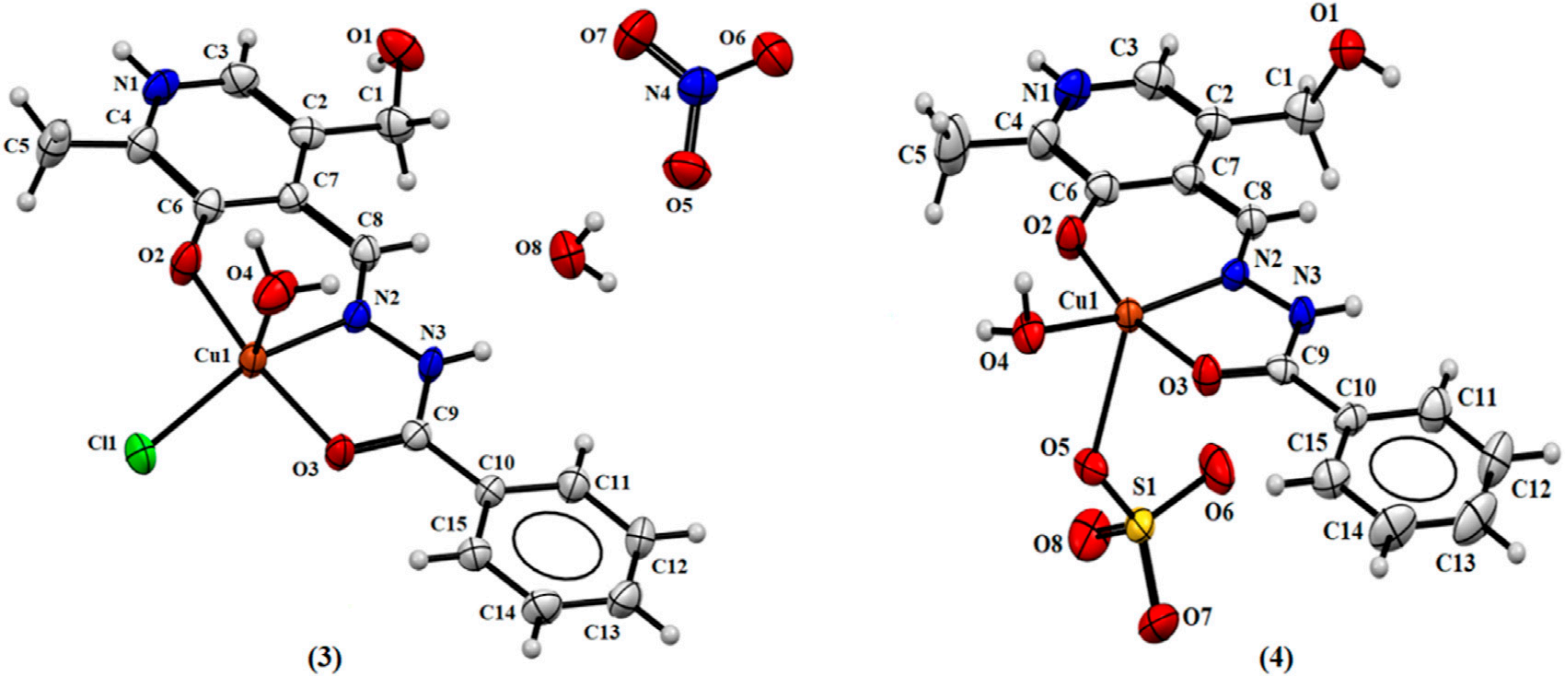
Molecular structure of (3) and (4) with crystallographic labeling (50% probability displacement).

The Cu(II) atoms in both complexes (3) and (4) show square pyramidal distorted geometry, with a water molecule and sulfate ion in the apical position of the pyramid, and Addison’s parameters of 0.262 and 0.045, respectively (Addison et al., 1984). The coordination mode of these complexes is similar to the two previous examples by the *ONO*-donor system. In the case of complex (3), there is, in addition, the coordination of a chloride ion and a water molecule, and the asymmetric unit shows a nitrate ion and a water molecule. In the case of complex (4), coordination of a sulfate ion and a water molecule occurs, and there are three water molecules in the asymmetric unit. Figure 3 shows the ORTEP representations of the structures of complexes (3) and (4).

Additionally, it is important to note that the substitution of the Cu(II) salt produces similar crystal structures with the same ligand coordination mode and with the metal center presenting a square pyramidal geometry. In complexes (1), (2), and (3), the Cu(II) ion is coordinated with Cl^−^, Br^−^, and SO_4_^−^, respectively, from the starting material. However, it was observed when Cu(NO_3_)_2_ was used that the NO_3_^−^ ion is not coordinated with the metal atom and there is a chloride ion coordinated with the free ligand.

It is noteworthy that, in all complexes, the ligand is in *zwitterionic* form with the protonation of the N1 nitrogen pyridine ring and deprotonation of the O2 atom, as reported in the literature for other similar metal complexes with pyridoxal ligands (Jevtovic et al., 2023; Jevtovic et al., 2022). The value found between 123.7 (7) and 125.3 (2)° for the bond angle C3–N1–C4 indicates the presence of a proton on the N1 atom, as this angle has been observed for deprotonated forms of similar ligands with the value of about 118° (Jevtovic et al., 2022). Comparing the free ligand PLBHZ (Back et al., 2009) with complexes (1–4), the bond distances found N2–N3 between 1.370 (8) and 1.387 (3)° and C9-O3 between 1.235 (9) and 1.259 (4)° are indicative of some electron delocalization throughout the hydrazone function with the coordination of the N3 and O3 atoms to the metal centers. This behavior is comparable with similar bond lengths previously reported (Vidovic et al., 2011; Jevtovic et al., 2022). Table 2 shows selected bond distances and angles for complexes (1–4).

**TABLE 2:** Selected bond lengths (Å) and angles (°) for complexes (1–4), where X = Cl (for 1 and 3) or X = Br (for 2).

Bond length (Å)				
	(1)	(2)	(3)	(4)
Cu1-O2	1.909 (2)	1.912 (5)	1.906 (2)	1.904 (2)
Cu1-N2	1.959 (3)	1.955 (6)	1.961 (2)	1.934 (2)
Cu1-O3	2.002 (2)	2.010 (5)	2.000 (2)	2.020 (2)
Cu1-X1	2.238 (2)	2.332 (2)	2.234 (7)	—
Cu1-X2	2.585 (2)	2.699 (2)	—	—
Cu1-O4	—	—	2.254 (2)	1.063 (2)
Cu1-O5	—	—	—	2.240 (2)
Bond angle (°)				
	(1)	(2)	(3)	(4)
O2-Cu1-N2	90.39 (11)	90.6 (2)	90.40 (7)	90.50 (8)
N2-Cu1-O3	79.88 (10)	79.3 (2)	79.86 (7)	80.16 (8)
O2-Cu1-X1	94.61 (8)	95.01 (16)	96.05 (6)	—
O3-Cu1-X1	92.36 (7)	92.34 (15)	92.69 (5)	—
X1-Cu1-X2	102.23 (4)	102.19 (5)	—	—
O3-Cu1-O4	—	—	85.66 (7)	94.22 (8)
O4-Cu1-O5	—	—	—	82.10 (7)
N2-Cu1-X1	161.75 (9)	161.64 (18)	154.52 (6)	—
O2-Cu1-O3	167.46 (10)	167.3 (3)	170.26 (7)	168.35 (8)

**FIGURE 4:**
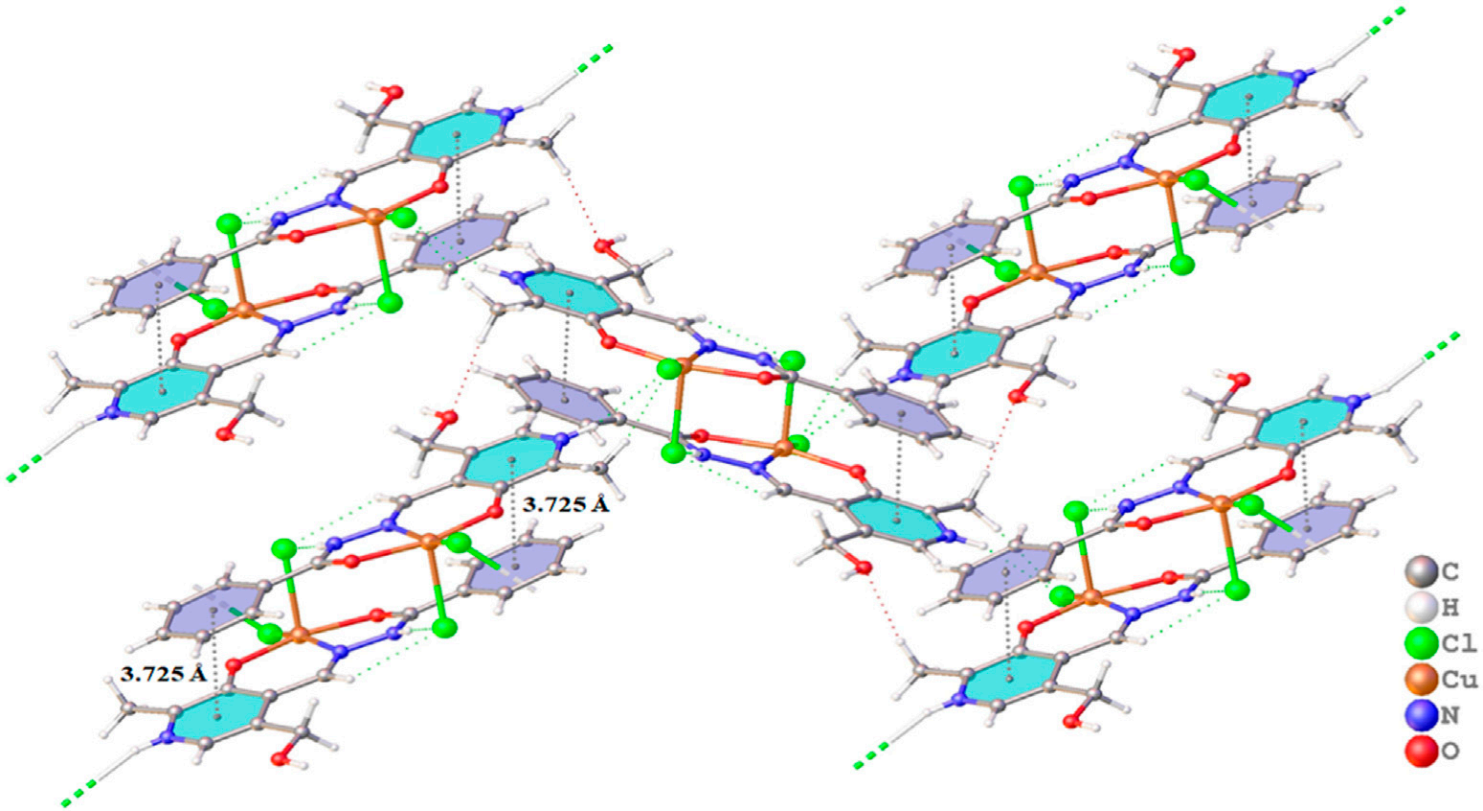
Projection view of (1) showing an intramolecular hydrogen bond and the π⋅⋅⋅π stacking interactions (as a dashed line).

The abundant non-covalent interactions observed, such as π⋅⋅⋅π stacking interactions and hydrogen bonds, are vital to connecting adjacent molecules to form supramolecular structures (Man et al., 2023). The different ions present in the unit cell play an important role in the formation of the intermolecular interactions and are essential for the formation and assembly of the complex. X-ray analysis data of all complexes show the presence of a π⋅⋅⋅π stacking interaction between the pyridine rings and benzene groups with distances between the centroids of 3.685 and 3.825 (5) Å (Supplementary Figures S1–S4). In all complexes, due to the presence of protonated nitrogen atoms N1 and N3, water molecules, and different ions, it is possible to find many intra- and intermolecular interactions. Figure 4 shows the π⋅⋅⋅π stacking interactions and hydrogen bonds in complex (1). Supplementary Table S1 lists the non-covalent interactions found in complexes (1–4).

### 3.2 Spectroscopy data

In order to certify the coordination modes of PLBHZ ligand in complexes (1–4), the vibrational infrared spectra of the free hydrazone and complexes were collected (Supplementary Figures S7–S11). The infrared spectrum of pyridoxal-benzohylhydrazone shows the ν(C=O) and ν(C=N) bands at 1,589 cm^−1^, and after the coordination of Cu(II) atom with the hydrazone, they are shifted to lower wavelength values and decrease the strength of the connection. The bands of ν(O-H) at 3,349 cm^-1^, ν(N-N) at 1053 cm^−1^, and ν(N–H) at 2936 cm^−1^ are observed for the free ligand. The ν(N–H) vibration was observed as broadband between 2830 and 2760 cm^−1^ in the spectrum of complexes (1–4), characteristic of the vibrational mode ν(N–H) in pyridine groups, suggesting that the PLBHZ acts as a neutral ligand (Sharma et al., 2020). The band of ν(C=O) shows in the ligand at 1669 cm^−1^; a shift of this band to lower and weak wave number by 70–60 cm^−1^ suggests the coordination of the carbonyl group to the metal ion (Jevtovic et al., 2022). The same behavior is observed for the strong band of ν(C=N) at 1589 cm^−1^; a shift of this band to lower wave number by 50–40 cm^−1^ suggests the coordination of the hydrazone to the metal ion through the azomethine nitrogen (Jevtovic et al., 2023). It is possible to find the band of ν(C-N) in the spectra of the complexes between 1,100 and 1,000 cm^−1^.

For the complexes, bands in the region of 3,600 cm^−1^ are associated with the asymmetric and symmetric OH stretching vibrations of water molecules. In (3), the strong absorption at 1,380 cm^−1^ is relative to the nitrate in ionic form, according to previous data (Burgos-Lopez et al., 2019). The presence of ν(SO_4_^2−^) in (4) at 1000 cm^−1^ confirms the monodentate coordination with the Cu(II) atom. Weak bands around 500 and 400 cm^−1^ that are not present in the spectra of the free ligand are assigned to ν(N1–Cu–O1), ν(Cu–N1), and ν(Cu–O2) modes in the complexes. The infrared analysis is according to similar complexes described previously and supports the crystal data analysis (Back et al., 2009; Gatto et al., 2019; Santiago et al., 2020).

It is possible to observe the absorption spectrum of free ligand bands corresponding to the π→π* transition of the azomethine group at around 285 nm in MeOH. The bands at 306 and 341 nm are attributed as being π→π* transitions of aromatic groups and n→π* of the hydrazone moiety, respectively, and underwent shifts upon complexation. The hypsochromic displacement of the π→π* band corresponding to the π→π* transition of the azomethine can be observed, which decreases to 326 nm in MeOH and 331 nm in DMF; this is indicative of the coordination of the group to the Cu(II) atom. The ligand–metal charge transition (LMCT) can be attributed in the range of 406–411 nm in MeOH and 413–415 nm in DMF as an indication of the coordination of the hydrazone to the Cu(II) atom (Santiago et al., 2022). In addition, to observe d-d transitions of the metal center, the electronic spectra of the complexes were determined at a higher concentration (2 mM) in methanol (Supplementary Figure S15 and Supplementary Table S3). The absorption bands of the d-d transitions were observed in the range of 672–680 nm, with values of log ε between 1.54 and 1.85 being more evidence of the complexation of the copper ion with the ligand (Ramírez-Contreras et al., 2022).

**FIGURE 5:**
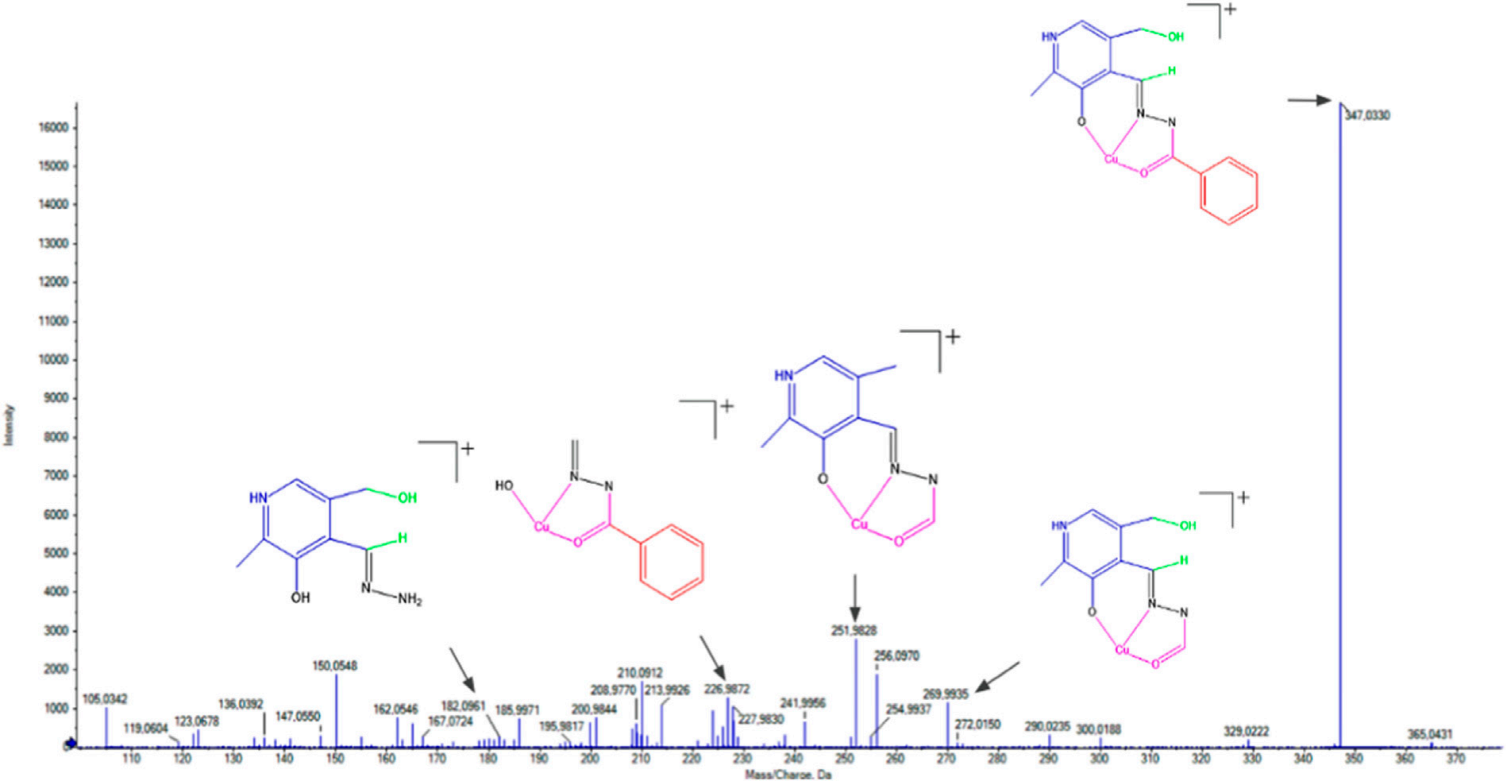
ESI(+)-MS/MS spectrum for (1).

ESI(+)-MS(/MS) electrospray mass spectra, with a concentration of 250 μM (methanol) in an acid medium (0.1% acetic acid), were obtained to evaluate the real species in the solution of the ligand and complexes. The spectra containing the fragmentations are shown in Figure 5 and Supplementary Figures S16–S20. In the ESI(+)-MS spectrum of the free hydrazone, it is possible to observe the isotopic distribution for the [M+H]^+^ ion of m/z = 286.1192 and a base peak at m/z=150.0551 for the substituted pyridine group; this can be explained by a rearrangement of the hydroxyl followed by an alpha cleavage of the azomethine group. Two other peaks can be observed at m/z=165.0663 and m/z=119.0612 due to the breaking of the azomethine group via a concerted mechanism.

In the case of complexes (1–4), it is possible to observe that some fragmentations are common to the ligand due to the similarity in their structures. The first peak corresponds to the molecular ion [M+H]^+^ after the loss of the coordinated ions and water molecules of m/z=347.0330 in (1), m/z=347.0326 in (2), m/z=347.0334 in (3), and m/z=347.0323 in (4). Other characteristic peaks observed are m/z=269.9929 in (1), m/z=269.9930 in (2), and m/z=269.9935 in (3) and (4) after the loss of the benzene group via alpha cleavage. The peaks m/z=251.9928 in (1), m/z=251.9825 in (2), m/z=251.9829 in (3), and m/z=251.9929 in (4) are observed due to the loss of water molecules by rearrangement of the hydroxyl group and the hydrogen attached to the azomethine group.

### 3.3 Hirshfeld Surface

The Hirshfeld surface (HS) allowed the evaluation of the electronic density distributions at the crystal structures to predict the intermolecular interactions between neighboring molecules of the complexes (McKinnon et al., 2004; Spackman and Jayatilaka, 2009). HS was obtained by the combination of normalized distances (*d*_*norm*_) between atoms inside (d_i_) and outside (d_e_) the surface. The contacts shorter than the sum of the van der Waals radii show a red color, close to the sum (white) and longer than the sum of the radii (blue).

**FIGURE 6:**
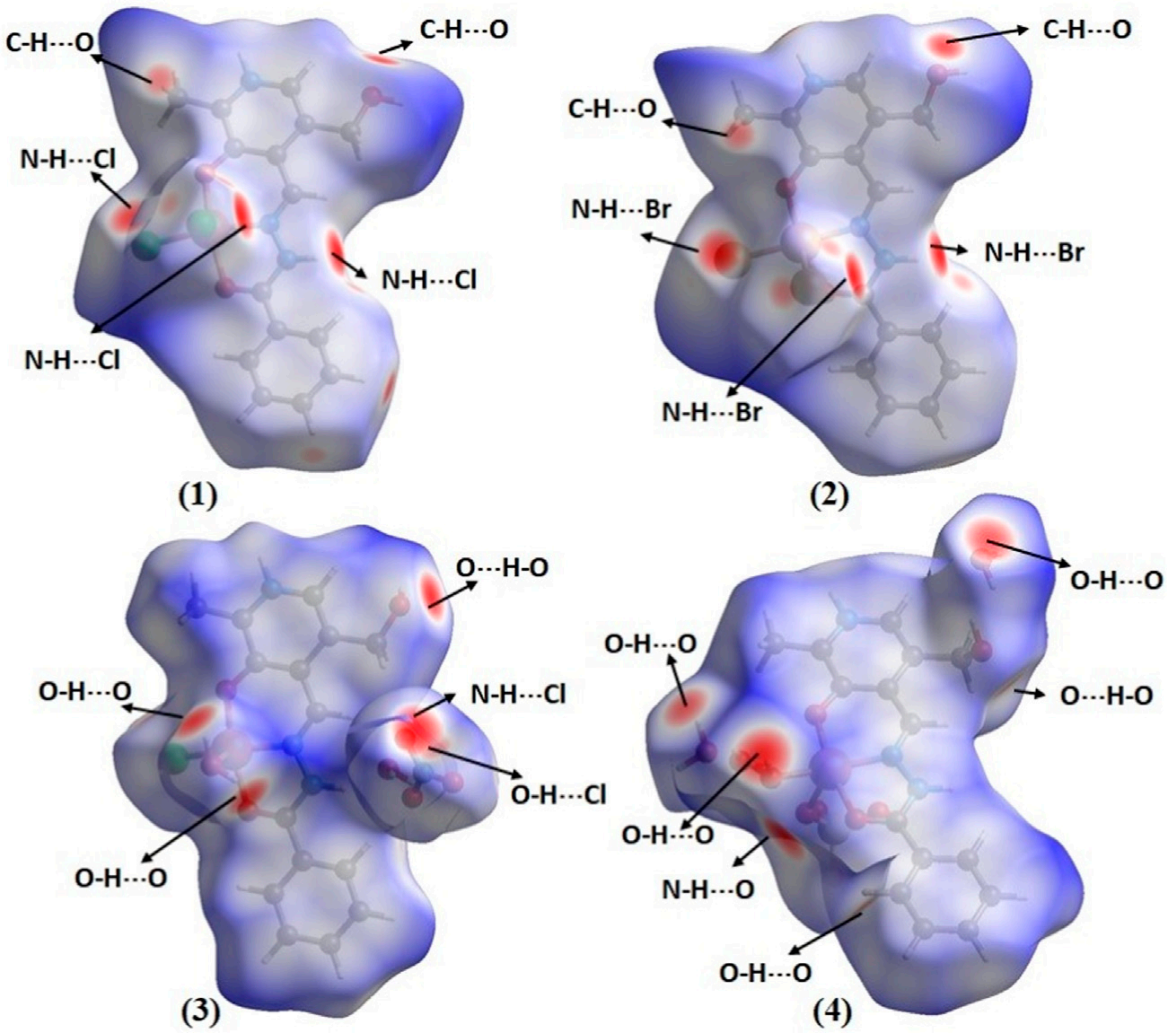
Hirshfeld Surface mapped with *d*_*norm*_ of complexes (1–4).

Red spots in the *d*_*norm*_ HS indicate the presence of hydrogen bonds in the structure of all complexes (Figure 6). The most intense red regions are related to N–H∙∙∙Cl and N–H∙∙∙Br in (1) and (2), respectively, with an internuclear distance between 2.35 and 2.58 Å, and O–H∙∙∙O in (3) and (4) with an internuclear distance between 2.00 and 2.43 Å. Non-classical interactions are verified in the HS of the four compounds, such as C–H∙∙∙O interactions (Man et al., 2023; Yilmaz et al., 2024). The shape index map allows a clearer observation of the π⋅⋅⋅π interactions, which show a pattern of red and blue triangles. The *shape index* for complexes (1–4) is shown in Supplementary Figure S21, where it is possible to observe the presence of π⋅⋅⋅π stacking interactions in all complexes, agreeing with the observed single-crystal X-ray diffraction.

The 2D-fingerprint plots (FP) associated with the Hirshfeld surface of the Cu(II) complexes (Supplementary Figures S22–S25) were obtained to verify the contribution of each contact and to help better comprehend the interactions occurring within the crystal (Man et al., 2023; Yilmaz et al., 2024). The H∙∙∙H contact contributes most to the molecular surface of the complexes with values 32.3% – 38%. The Cl∙∙∙H, Br∙∙∙H, and O∙∙∙H interactions appear as long and narrow spikes in the FP, demonstrating that these interactions are strong and intense. The C∙∙∙C contacts have contribution values between 5% and 7% due to the presence of π∙∙∙π stacking interactions in all compounds.

### 3.4 Full interaction maps

**FIGURE 7:**
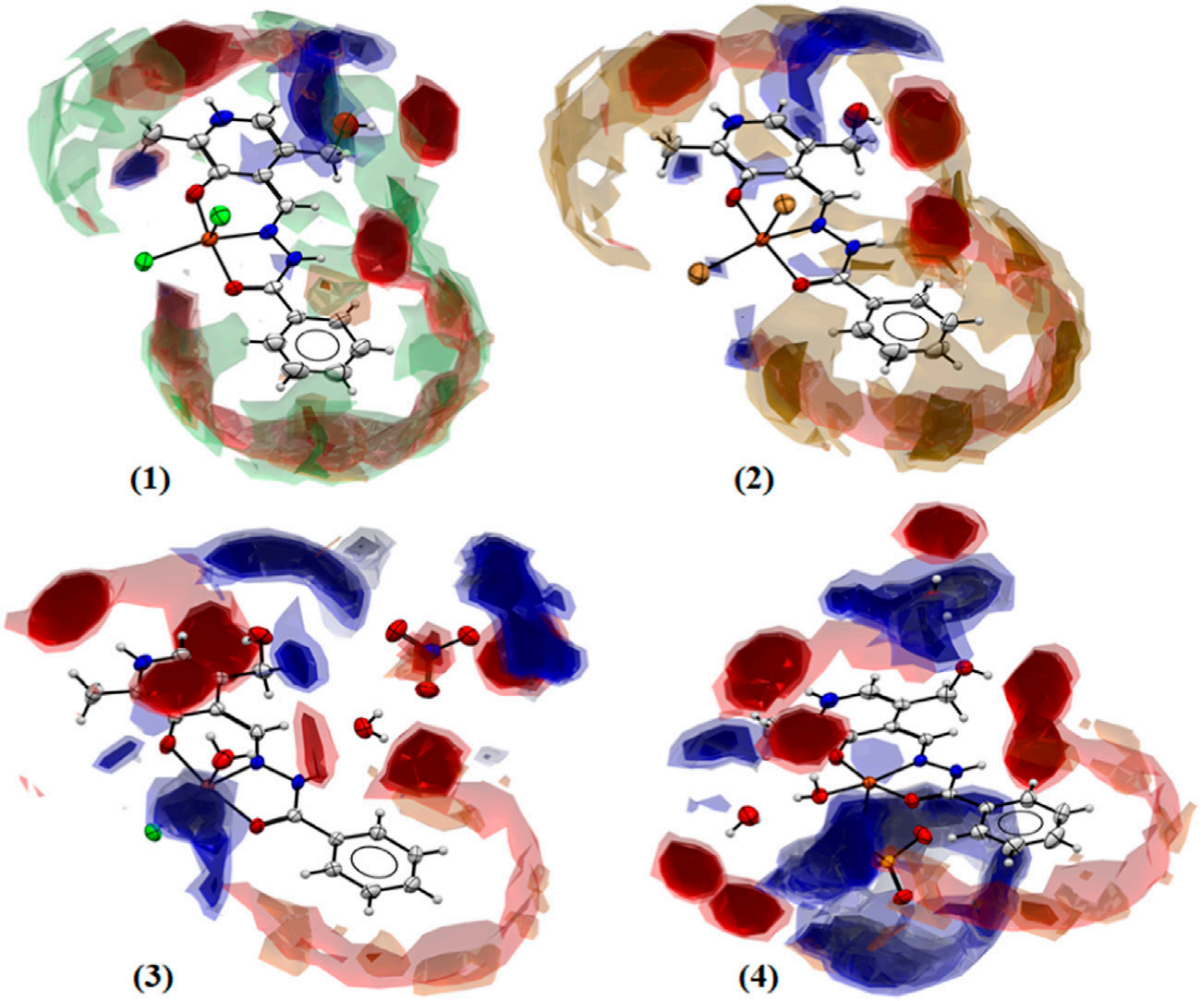
Full interaction maps for complexes (1–4) showing the acceptor and donor likelihood regions, and hydrophobic groups.

The full interaction maps (FIMs) were used to predict the most likely locations for a variety of acceptor and donor groups in the crystal structures and evaluate the preferred interactions of the molecules (Wood et al., 2013). The interaction maps highlight the red regions that indicate the acceptor positions and the blue regions where the donor atoms are expected. Figure 7 shows the FIMs of complexes (1–4) where the intense red regions near the NH or OH are indicative of hydrogen bond acceptors, and the intense blue regions near oxygen atoms are indicative of hydrogen bond donors. Large and intense red and blue regions are observed in complexes (3) and (4) due to the presence of solvent water molecules, nitrate, and sulfate ions. The hydrophobic regions in brown can be found above and below the phenyl rings of the ligands. The green color found for complex (1) in the FIM is indicative of noncovalent interactions with the chloride ions.

### 3.5 Biological activity analysis

**FIGURE 8:**
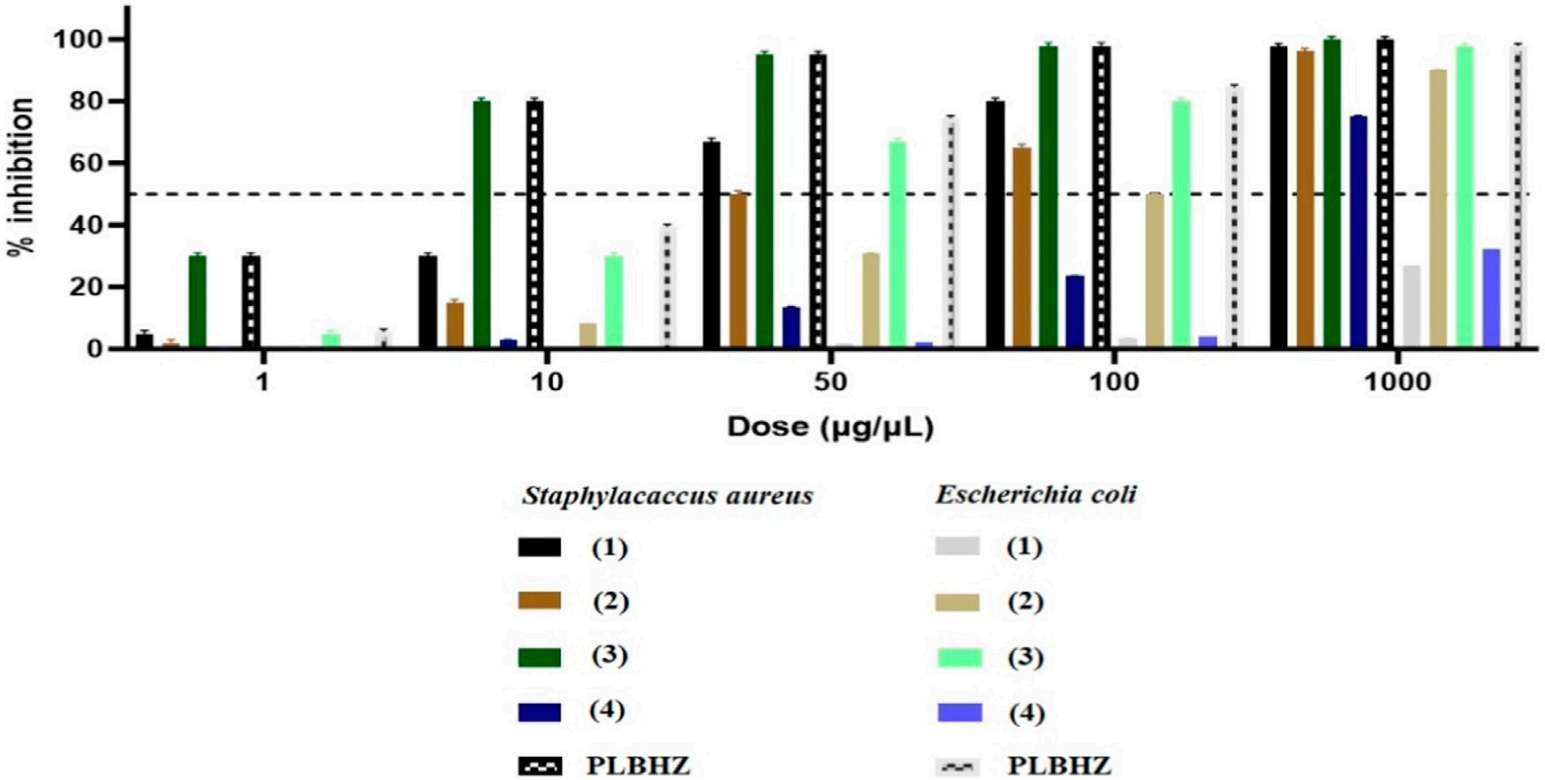
Percentage of inhibition for minimal inhibitory concentration (μg/mL) of the synthesized compounds.

To investigate the antibacterial activity of the synthesized compounds, the free ligands and the Cu(II) complexes were tested on Gram-positive *S. aureus* and Gram-negative *E. coli* bacteria strains. The MICs of the compounds were determined, corresponding to the lowest concentration of the tested compound necessary to inhibit the growth of a microorganism, and are compared in Figure 8. Table 3 shows the tested different formulations for antimicrobial activity and the MIC values determined for PLBHZ and their metal complexes (1–4).

**TABLE 3:** Antibacterial activity was determined for PLBHZ and complexes (1–4), expressed as MIC and MBC (μg⋅mL^−1^) for the different formulations against *S. aureus* and *E. coli* strains.

Compound	*S. aureus*			*E. coli*		
	MIC	IC {IC_50_}	IC 99.9 {MBC}	MIC	IC {IC_50_}	IC 99.9 {MBC}
PLBHZ	1.37 × 10^−6^	0.001–0.004	1.37	1.73 × 10^−6^	0.001–0.032	1.73
**(1)**	3.48 × 10^−5^	0.012–0.043	24.95	3.11 × 10^−3^	2.250–3.251	2997.03
**(2)**	5.15 × 10^−5^	0.02–0.09	49.95	9.83 × 10^−4^	0.070–0.156	69.82
**(3)**	6.34 × 10^−5^	0.002–0.034	12.99	3.86 × 10^−4^	0.01–0.04	21.32
**(4)**	3.51 × 10^−4^	0.21–0.43	350.84	3.51 × 10^−1^	3.4–3.9	3,696.83

The compounds were generally more effective on Gram-positive than Gram-negative bacteria. The results show that the free ligand and complexes (1–3) presented a better MIC value than the other complexes for Gram-positive bacteria, and that complex (3) presented a lower MIC value for Gram-negative bacteria. The free ligand and complexes (1–3) presented IC_50_ values lower than the positive control oxacillin used for *S. aureus* with an IC_50_ of 0.05 μg mL^−1^. However, PLBHZ and complexes 2 and 3 presented IC_50_ values much lower than the positive control, ampicillin with IC_50_ of 1.78 μg mL^−1^, for *E. coli*. The results of the biological activity also show various compounds with significant antibacterial activity against *S. aureus* (MIC < 1 μg mL^−1^) (Gibbons, 2004). Comparing the obtained values, it is possible to verify the high antimicrobial activity of the hydrazone before coordination with the Cu(II) ion. In contrast, Gram-negative bacteria have an asymmetrical functional lipid bilayer outer membrane that protects them from harmful chemicals present in their environment. This outer membrane is composed mainly of phospholipids in the inner leaflet and lipopolysaccharides (LPS) in the outer layer. Due to the polar nature of the LPS, the outer membrane is charged and creates a significant barrier against lipophilic molecules, thus protecting the bacteria from various detergents (e.g., bile salts), dyes (e.g., methylene blue), and hydrophobic antibiotics (Neidhardt and Roy, 1996). In addition to containing lipoproteins, the outer membrane features porin proteins, with their conserved β-barrel fold that encloses a central aqueous channel that regulates the passage of hydrophilic molecules (Koebnik et al., 2000).

### 3.6 Molecular docking simulation

The molecular modeling of the complexes formed by the MERC1 and OpmF/A enzymes and the small molecules was performed using a molecular docking simulation with AutoDock Vina. The protocols for the docking simulations were validated through previous redocking studies with crystallographic structures of the complexes and their ligands molecules, oxacilloyl (*S. aureus*) and ampicillin (*E. coli*). In both cases, the superposition of the docked pose and crystallographic pose agree well (see Supplementary Figure S26). The RMSD was 0.86 Å for MecR1-oxacilloyl and 1.36 for the OmpF/A-ampicillin system. After establishing the docking protocol, the molecular modeling of both systems proceeded. The docking simulation revealed good agreement in the tendency of the inhibitory power with the experimental IC_50_ values for both enzymatic systems and their ligands. The same trend of inhibition was observed comparing the antibacterial activity in the experimental essay (Table 3) and the theoretical energy of binding (score values in Table 4).

**TABLE 4:** Theoretical binding energy (score) of docking studies with the complexes systems formed between Gram-positive and Gram-negative bacteria and the small molecules PLBHZ, complexes (1–4), and control molecules oxacillin and ampicillin.

*S. aureus* (Bioreceptor: MecR1—PDB 2IWD)		*E. coli* (Bioreceptor.: OmpF/A—PDB 4GCP)	
Compound	Score (kcal mol^−1^)	Compound	Score (kcal mol^−1^)
PLBHZ	−8.0	PLBHZ	−7.9
(1)	−7.5	(1)	−6.8
(2)	−6.9	(2)	−7.0
(3)	−7.7	(3)	−7.4
(4)	−6.7	(4)	−6.6

**FIGURE 9:**
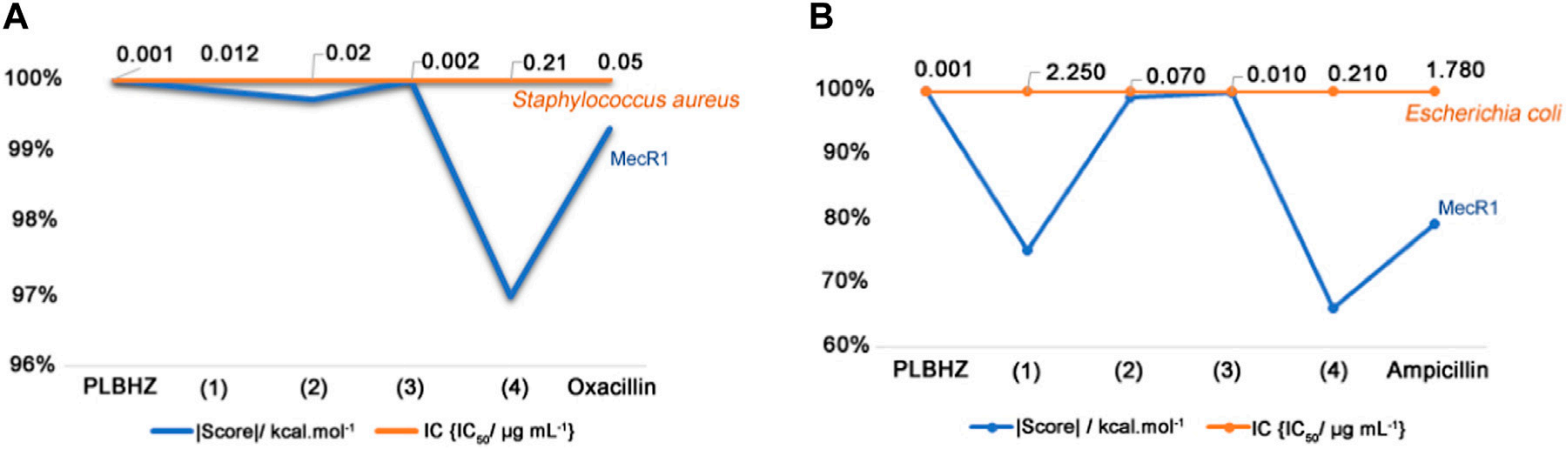
Relative trend of inhibition and the correlation to IC_50_ versus *in silico* energy of binding (score). **(A)** *S. aureus*—MecR1, **(B)** *E. coli*—OmpF/A.

The relative trend of inhibition to quantitatively evaluate the correlation and assertiveness between the experimental values of IC_50_ versus the *in silico* energy of binding (score) is shown in Figure 9. For the *S. aureus* proteomic system (Figure 9A), the order of power of inhibition of the studied molecules is PLBHZ > (3) > (1) > (2) > oxacillin > (4); the trend of the power of inhibition for the *E. coli* proteomic system (Figure 9B) is PLBHZ > (3) > (2) > ampicillin > (1) > (4). The PLBHZ and compound (3) are the most powerful inhibitors for both enzymes in the set of ligands studied, and compound (4) presents for both bacteria a low power of inhibition.

In general, the compounds show a promising inhibitory power against Gram-positive and Gram-negative bacteria, although the experimental and theoretical studies pointed to a better affinity between the Gram-positive bacteria system—*S. aureus*.

#### 3.6.1 Molecular docking simulation of MecR1 *S. aureus*

**FIGURE 10:**
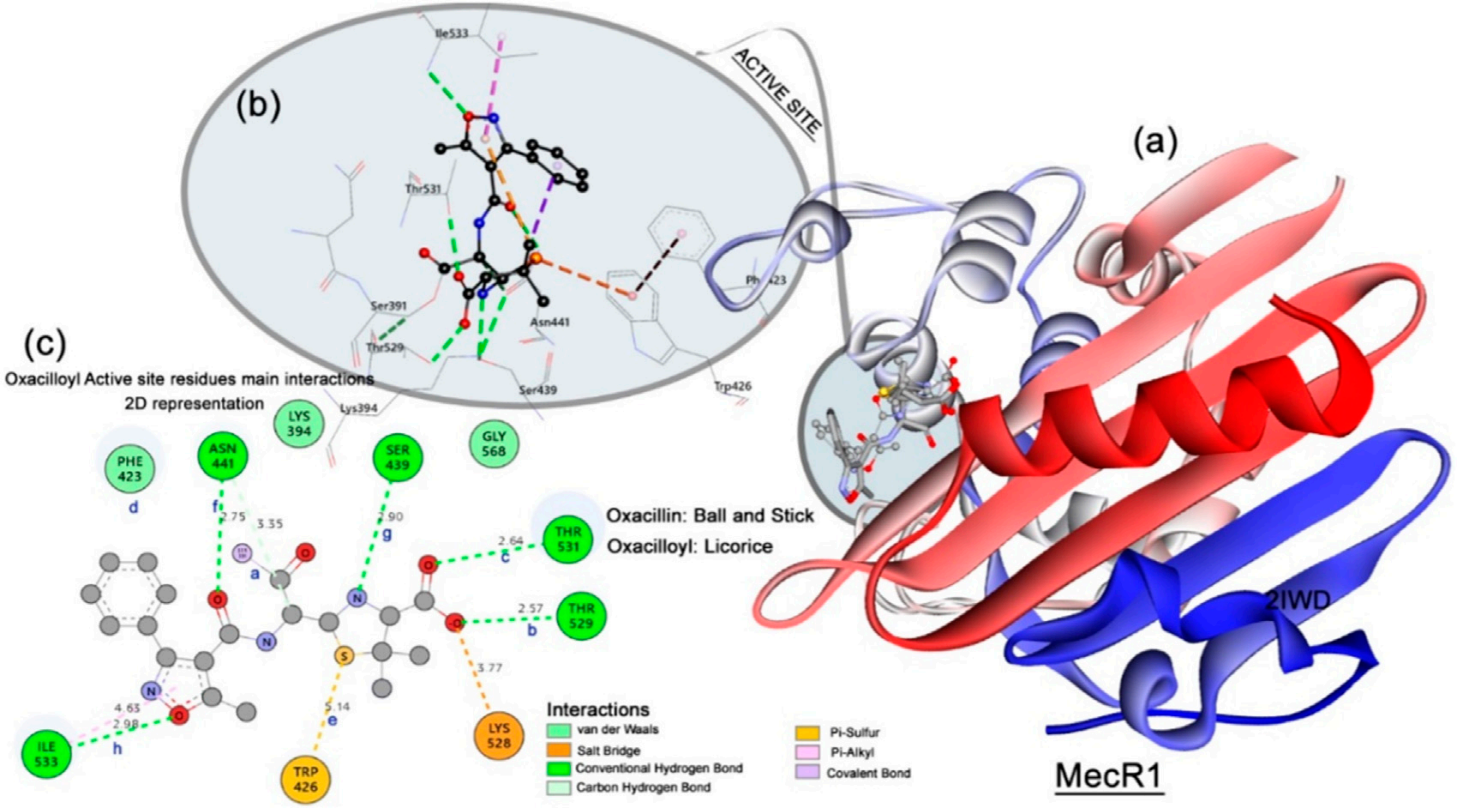
MecR1 structure. **(A)** Enzyme N-to-C terminal representation, superposition between the oxacilloyl (represented in licorice), and best docking pose of oxacillin (represented in balls and stick). **(B)** 3D views of active site and main residues interacting with oxacilloyl—MecR1. **(C)** 2D interaction and distance map of the complex formed by MecR1 and oxacilloyol adduct. The distances are set as follows: **a** Ser391:OG⋅⋅⋅C1:ligand; **b** Thr529:OG⋅⋅⋅O11:ligand; **c** Thr531:OG⋅⋅⋅O12:ligand; **d** Phe423:centroid⋅⋅⋅phenyl ring (centroid):ligand; **e** Trp426:phenyl ring centroid⋅⋅⋅S2:ligand; **f** Asn411:ND2O16:ligand.

The crystallographic structure of the MecR1 (**PDB** 2IWD) bioreceptor representing the proteomic of *S. aureus* is originally complexed with the reacted oxacillin, forming a covalent bond with the catalytic residue Ser391, thus forming the oxacilloyil adduct (Figure 10A). The docking study for oxacilloyl and oxacillin molecules was performed and showed good agreement in superposition with the original crystallographic structure.

As presented in Figure 10B, the active site of the MecR1 enzyme is compound of catalytic Ser391 residue and Pro389, Asn390, Thr392, Lys394, Ser439, Phe423, Trp426, Asn441, Thr529, Thr531, and Ile533 residues. To block the activity of the MecR1, and consequently interrupt the machinery of resistance in the *S. aureus*, a covalent interaction (set as **a**, see Figure 10C) between the antibacterial inhibitor and the Ser391 active site residue is necessary. In addition, some important interactions were observed by Marrero et al. (2006) for effective inhibition.

Important hydrogen bonds should be established between the Thr529 (2.50 Å), Thr531 (2.70 Å), and Asn441 (2.80 Å) residues (set as **b**, **c**, and **f** in Figure 10C) to perform efficient inhibition. Furthermore, medium range pi–alkyl, pi–sulfur interaction types between the adduct specie and the residues Phe423 (set as **d**, Figure 10C) and Trp426 (set as **e**, Figure 10C) residues are very important for stabilizing the adduct in the active site of MecR1. Table 5 presents the main interactions performed for the ligand PLBHZ, complexes (1–4), oxacilloyl, and oxacillin during the molecular docking study with the MecR1 enzyme, with distances (Marrero et al., 2006).

**TABLE 5:** Docking main interactions and distances (measured in Å) shown in Figure 10C performed in the complexes formed by MecR1 inhibitors.

Interaction	Oxacilloyl	Oxacillin	PLBHZ	(1)	(2)	(3)	(4)
**a**	1.45^a^	2.36	2.85	>6.00	>6.00	3.80	>6.00
**b**	2.42	2.96	2.79	2.51	2.63	3.00	2.96
**c**	2.64	2.42	2.35	3.50	4.00	2.11	2.47
**d**	6.00	5.47	5.37	4.13	4.30	4.41	4.58
**e**	5.14	5.59	4.60	>6.00	>6.00	>6.00	>6.00
**f**	2.75	2.96	2.96	>6.00	>6.00	3.80	>6.00

^a^ Covalent bond length between the atoms Ser391:OG … C1:adduct.

The inhibitory power of the ligands is related to the strong, short-length hydrogen bond interactions between the compounds and the residues Thr529, Thr531, and Asn441. The pi–alkyl interaction with the Phe423 significantly stabilizes the ligand in position to react with the catalytic Ser391 residue.

The best-ranked compound as a good inhibitor for MecR1 was the ligand PLBHZ, followed by (3) and (1). The measured distances shown in Table 5 reveal that PLBHZ is the molecule that interacts with all active site residues with short distances for **c**, **d**, and **e** distances, and also mimics the profiles of interaction of the oxacillin. The shorter hydrogen interaction with the residues Thr531 (**c**) and Asn411 (**f**), observed with PBLHZ, and oxacillin suggests the importance of this range length during the inhibition process. In addition, the closer range length of the interactions (**d**) and (**e**) shows the same importance in the inhibitory process.

#### 3.6.2 Molecular docking simulation of OmpF/A *E. coli*

**FIGURE 11:**
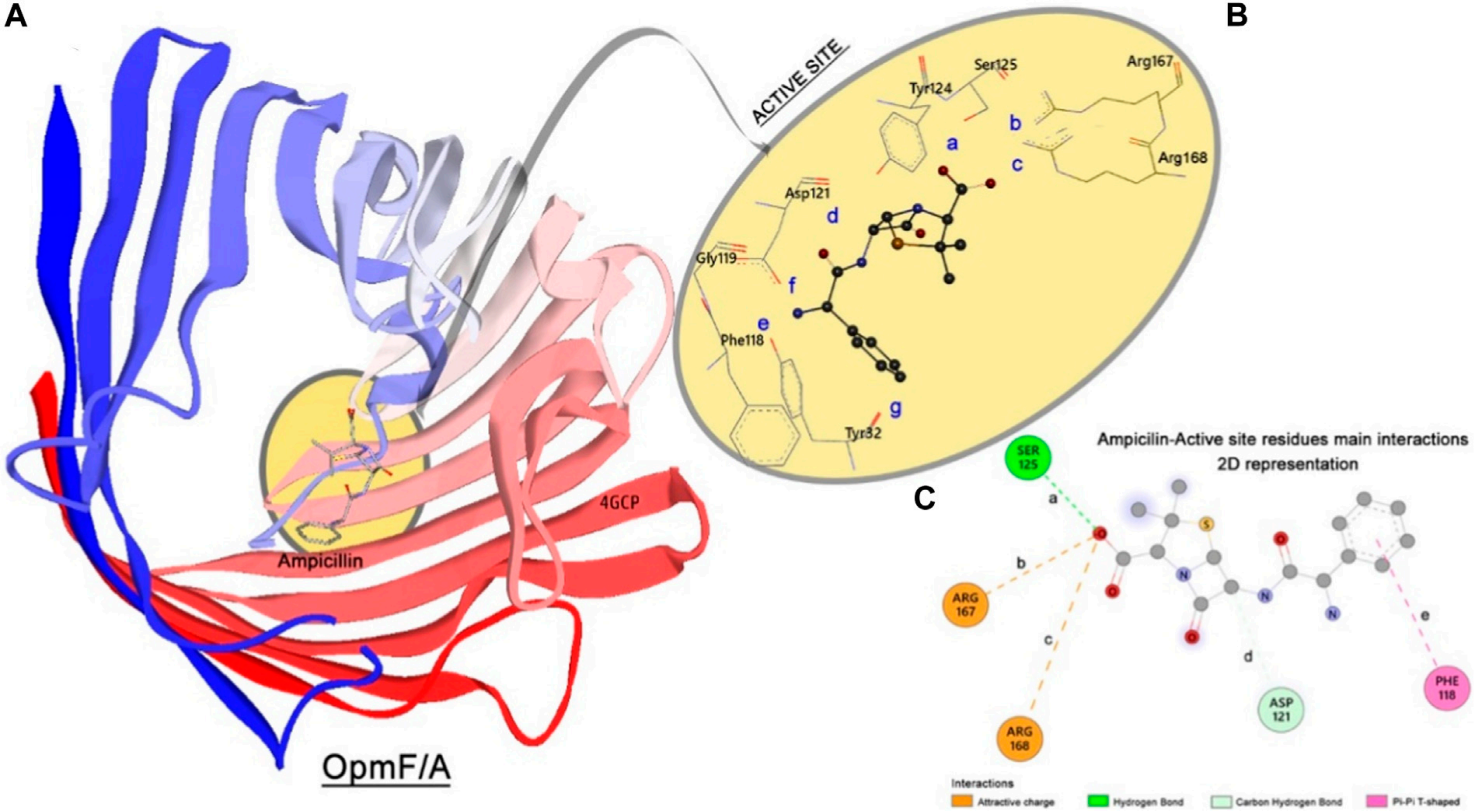
OmpF/A structure. **(A)** Enzyme–enzyme N-to-C terminal representation, complexed with ampicillin (represented in licorice). **(B)** 3D views of active site and main residues interactions. **(C)** 2D interaction and distance map of the complex formed by OmpF/A and ampicillin. The distances are set as follows: **a** Ser125:OG⋅⋅⋅O1:ligand; **b** Arg167:NZ⋅⋅⋅O1:ligand; **c** Arg168:NZ⋅⋅⋅O1:ligand; **d** Asp121:OD2⋅⋅⋅C1:ligand; **e** Phe118:centroid⋅⋅⋅centroid of phenyl ring of the ligand.

The crystallographic structure of the OmpF (**PDB** 4GCP) bioreceptor from proteomic of the *S. aureus* bacteria complexed with ampicillin is shown in Figure 11A.

The crystallographic 3D structure of Ompf/a reveals that ampicillin is oriented perpendicular to the axis of the channel. This orientation promotes proper electrostatic interactions between the negatively charged carboxylic group of ampicillin and the positive NH_3_^+^ groups of the residues Arg167 (**b**) and Arg168 (**c**), Figure 11B. These interactions stabilized the complex. Other important interactions are the hydrogen bond type, observed between the Ser125 (**a**) and the hydroxyl group of the side chain of ampicillin, and the non-conventional hydrogen bond between the Asp121 (**d**) residue and the hydrogen of the C1 atom of ampicillin. The phenyl ring of ampicillin is located in the hydrophobic pocket composed of several aromatic residues, especially the residues Tyr22, Tyr32, and Phe118. The phenyl ring of ampicillin makes a π⋅⋅⋅π T-shaped interaction with the phenyl ring of Phe118 residue (**e**), Table 6. This interaction is responsible for stabilizing the ampicillin in the active site of OmpF/a without inducing a large conformational change in the protein active site (Ziervogel and Roux, 2013).

**TABLE 6:** Docking main interactions and distances (measured in Å) shown in Figure 11C performed in the complexes formed by OmpF and A-inhibitors.

Interaction	Ampicillin	PLBHZ	(1)	(2)	(3)	(4)
**a**	2.92	2.80	3.74	>6.00	3.04	3.77
**b**	4.33	5.12	>6.00	>6.00	4.21	4.97
**c**	5.44	5.34	4.21	4.83	3.64	>6.00
**d**	3.64	3.75	4.60	3.15	3.10	4.85
**e**	5.64	4.24	5.10	4.92	3.60	5.97

The PLBHZ ligand presents stronger hydrogen bond interaction with the residue Ser125 (**a**: 2.80 Å) than other ligands, including ampicillin. The non-conventional hydrogen-bond interaction between the Asp121 (**d**) residue and the ligands plays an important role in the inhibitory process, comparing the size of the distance for this interaction and the inhibitory trend obtained through the experimental essay and *in silico* approach. [PLBHZ > (3) > (2) > ampicillin > (1) > (4)] is the correlation between the length of this interaction and the binding energy of the compounds.

The π⋅⋅⋅π T-shaped interaction with the Phe118 (**e**) residue was also shown to be related directly to the power of inhibition presented by the molecules studied. For this interaction, the PLBHZ and the organometallic compound (3) length suggest that lower values (less than 5.00 Å) stabilize the ligand in a better position, and the binding energy increases (Table 4).

According to the experimental and theoretical data, the PLBHZ and the organic metallic compounds generally show good inhibitory activity against Gram-positive and Gram-negative bacteria. A better performance following the values of IC_50_ and the energy of binding was observed for *S. aureus*. The superposition of the best-scored poses (from molecular docking results) comparing both bioreceptor systems shows better alignment between the compounds studied here and the control molecule, oxacillin, in the MecR1, *S. aureus* (see Supplementary Figure S27) enzyme. For the *E. coli* (see Supplementary Figure S28) system, the score values observed were less effective than for the *S. aureus* bacteria enzyme.

Complex (4) showed less activity in forming a complex with both bioreceptor systems. It seems that the high volume of the group SO_4_^2-^ adds a stereo hindrance to the molecule, increasing the spheric coordination of the complex with the Cu(II) metal and imposing a spatial restriction that blocks the compound from entering the active site to properly interact with its residues.

## 4 Conclusion

Four new Cu(II) complexes with pyridoxal-benzohydrazone and different Cu(II) salts were successfully synthesized, and the metal centers showed square pyramidal geometry. The spectroscopy analysis revealed that the different ions in the crystal structures have no significant influence on the spectral properties of the complexes. In addition, non-covalent interactions were detected in all compounds, showing the relevance of pyridoxal, water molecules, and different ions in establishing hydrogen bonds. Hirshfeld surface analysis combined with 2D fingerprint plots showed that the different anions in the Cu(II) complexes, together with their noncovalent interactions, are essential in determining the final crystal structure networks. The results of the biological evaluation and molecular docking of the new Cu(II) complexes with pyridoxal-benzoylhydrazone can allow the development of innovative new strategies for developing potential new drugs.

## Data Availability

The datasets presented in this study can be found in online repositories. The names of the repository/repositories and accession number(s) can be found in the article/Supplementary Material.

## References

[B1] Addison A. W. Rao T. N. Reedijk J. van Rijn J. Verschoor G. C. (1984). Synthesis, structure, and spectroscopic properties of copper(II) compounds containing nitrogen–sulphur donor ligands; the crystal and molecular structure of aqua[1,7-bis(N-methylbenzimidazol-2′-yl)-2,6-dithiaheptane]copper(II) perchlorate. J. Chem. Soc. Dalt. Trans., 1349–1356. 10.1039/dt9840001349

[B2] Arora T. Devi J. Boora A. Taxak B. Rani S. (2023). Synthesis and characterization of hydrazones and their transition metal complexes: antimicrobial, antituberculosis and antioxidant activity. Res. Chem. Intermed. 49, 4819–4843. 10.1007/s11164-023-05116-1

[B3] Askarian S. Beyramabadi S. A. Badmasti F. Heravi F. S. Tabrizi A. M. A. Azizi H. (2021). Synthesis, characterization and *in vitro* evaluation of cytotoxicity and antibacterial properties of vanadyl complexes of the pyridoxal Schiff bases. J. Mol. Struct. 1246, 131189. 10.1016/j.molstruc.2021.131189

[B4] Back D. F. Ballin M. A. de Oliveira G. M. (2009). Synthesis and structural features of UVI and VIV chelate complexes with (hhmmbH)Cl·H2O [hhmmb = {3-hydroxyl-5-(hydroxymethyl)-2-methylpyridine-4-yl-methylene}benzohydrazide], a new Schiff base ligand derived from vitamin B6. J. Mol. Struct. 935, 151–155. 10.1016/j.molstruc.2009.07.005

[B5] Biovia D. S. Release H. (2020). Biovia Discovery Studio visualizer. San Diego: Biovia.

[B6] Boulechfar C. Ferkous H. Delimi A. Djedouani A. Kahlouche A. Boublia A. (2023). Schiff bases and their metal Complexes: a review on the history, synthesis, and applications. Chem. Commun. 150, 110451. 10.1016/j.inoche.2023.110451

[B7] Burgos-Lopez Y. Del Plá J. Balsa L. M. León I. E. Echeverría G. A. Piro O. E. (2019). Synthesis, crystal structure and cytotoxicity assays of a copper(II) nitrate complex with a tridentate ONO acylhydrazone ligand. Spectroscopic and theoretical studies of the complex and its ligand. Inorganica Chim. Acta 487, 31–40. 10.1016/j.ica.2018.11.039

[B8] Casas J. S. Couce M. D. Sordo J. (2012). Coordination chemistry of vitamin B6 and derivatives: a structural overview. Coord. Chem. Rev. 256, 3036–3062. 10.1016/j.ccr.2012.07.001

[B9] Dherbassy Q. Mayer R. J. Muchowska K. B. Moran J. (2023). Metal-pyridoxal cooperativity in nonenzymatic transamination. J. Am. Chem. Soc. 145, 13357–13370. 10.1021/jacs.3c03542 37278531

[B10] Dolomanov O. V. Bourhis L. J. Gildea R. J. Howard J. A. K. Puschmann H., (2009). *OLEX2*: a complete structure solution, refinement and analysis program. Appl. Crystallogr. 42, 339–341. 10.1107/s0021889808042726

[B11] Fekri R. Salehi M. Asadi A. Kubicki M. (2019). Synthesis, characterization, anticancer and antibacterial evaluation of Schiff base ligands derived from hydrazone and their transition metal complexes. Inorganica Chim. Acta 484, 245–254. 10.1016/j.ica.2018.09.022

[B12] Gamov G. A. Kiselev A. N. Zavalishin M. N. Yarullin D. N. (2023). Formation and hydrolysis of pyridoxal-5′-phosphate hydrazones and Schiff bases: prediction of equilibrium and rate constants. J. Mol. Liq. 369, 120961. 10.1016/j.molliq.2022.120961

[B13] Gan L.-L. Li X. Du M.-X. Yan Y.-J. Zhang Y. Dong W.-K. (2024). Study on solvent effect and coordination characteristics of two different types of Cu(II) salamo-salen-salamo-hybrid complexes. J. Mol. Struct. 1299, 137199. 10.1016/j.molstruc.2023.137199

[B14] Gatto C. C. Chagas M. A. S. Lima I. J. Mello Andrade F. Silva H. D. Abrantes G. R. (2019). Copper(II) complexes with pyridoxal dithiocarbazate and thiosemicarbazone ligands: crystal structure, spectroscopic analysis and cytotoxic activity. Transit. Metall. Chem. 44, 329–340. 10.1007/s11243-018-00299-8

[B15] Gibbons S. (2004). Anti-staphylococcal plant natural products. Nat. Prod. Rep. 21, 263. 10.1039/b212695h 15042149

[B16] Gupta S. (2022). Recent reports on Pyridoxal derived Schiff base complexes. Rev. Inorg. Chem. 42, 161–177. 10.1515/revic-2020-0026

[B17] Jevtovic V. Alshamari A. K. Milenković D. Dimitrić Marković J. Marković Z. Dimić D. (2023). The effect of metal ions (Fe, Co, Ni, and Cu) on the molecular-structural, protein binding, and cytotoxic properties of metal pyridoxal-thiosemicarbazone complexes. Int. J. Mol. Sci. 2023, 24. 10.3390/ijms241511910 PMC10419307 37569285

[B18] Jevtovic V. Alshammari N. Latif S. Alsukaibi A. K. D. Humaidi J. Alanazi T. Y. A. (2022). Synthesis, crystal structure, theoretical calculations, antibacterial activity, electrochemical behavior, and molecular docking of Ni(II) and Cu(II) complexes with pyridoxal-semicarbazone. Molecules 27, 6322. 10.3390/molecules27196322 36234859 PMC9570950

[B19] Karthick K. A. Shankar B. Gayathri S. Aravind M. K. Ashokkumar B. Tamilselvi A. (2023). Dual responsive pyridoxal-AHMT based fluorescent sensor towards zinc(ii) and mercury(ii) ions and its bioimaging application. New J. Chem. 47, 9427–9439. 10.1039/d3nj00890h

[B20] Kejík Z. Kaplánek R. Havlík M. Bříza T. Vavřinová D. Dolenský B. (2016). J. Lumin. 180, 269.

[B21] Koebnik R. Locher K. P. Van Gelder P. (2000). Structure and function of bacterial outer membrane proteins: barrels in a nutshell. Mol. Microbiol. 37, 239–253. 10.1046/j.1365-2958.2000.01983.x 10931321

[B22] Li X.-X. Ma C.-Y. Du M.-X. Dong W.-K. Ding Y.-J. (2024). A rare salamo-salophen type “on-off-on” fluorescent probe for relay recognition of Hg2+ and phosphate ions and its applications. J. Mol. Struct. 1299, 137188. 10.1016/j.molstruc.2023.137188

[B23] Liu R. Cui J. Ding T. Liu Y. Liang H. (2022). Research progress on the biological activities of metal complexes bearing polycyclic aromatic hydrazones. Molecules 27, 8393. 10.3390/molecules27238393 36500482 PMC9739244

[B24] Ma J. Gao B. Song G. Zhang R. Wang Q. Ye Z. (2022). Asymmetric α‐allylation of glycinate with switched chemoselectivity enabled by customized bifunctional pyridoxal catalysts. Angew. Chem. Int. Ed. 61, e202200850. 10.1002/anie.202200850 35182094

[B25] Macrae C. F. Sovago I. Cottrell S. J. Galek P. T. A. McCabe P. Pidcock E. (2020). Mercury 4.0: from visualization to analysis, design and prediction. Appl. Crystallogr. 53, 226–235. 10.1107/s1600576719014092 PMC6998782 32047413

[B26] Man L.-L. Ding Y.-F. Li X. Tong L. Dong W.-K. (2023). Exploring the structural diversities of three Co(II) complexes constructed from a new quinoline-containing N3O-cavity half-salamo type ligand and various counteranions. Polyhedron 245, 116654. 10.1016/j.poly.2023.116654

[B27] Marrero A. Mallorquí-Fernández G. Guevara T. García-Castellanos R. Gomis-Rüth F. X. (2006). Unbound and acylated structures of the MecR1 extracellular antibiotic-sensor domain provide insights into the signal-transduction system that triggers methicillin resistance. J. Mol. Biol. 361, 506–521. 10.1016/j.jmb.2006.06.046 16846613

[B28] McKinnon J. J. Spackman M. A. Mitchell A. S. (2004). Novel tools for visualizing and exploring intermolecular interactions in molecular crystals. Acta Crystallogr. B 60, 627–668. 10.1107/s0108768104020300 15534375

[B29] Morris G. M. Huey R. Lindstrom W. Sanner M. F. Belew R. K. Goodsell D. S. (2009). AutoDock4 and AutoDockTools4: automated docking with selective receptor flexibility. J. Comput. Chem. 30, 2785–2791. 10.1002/jcc.21256 19399780 PMC2760638

[B30] Murašková V. Eigner V. Dušek M. Sedmidubský D. (2021). Iron(III) and cobalt(III) complexes with pentadentate pyridoxal Schiff base ligand – structure, spectral, electrochemical, magnetic properties and DFT calculations. Polyhedron 197, 115019. 10.1016/j.poly.2021.115019

[B31] Nascimento É. C. M. Oliva M. Świderek K. Martins J. B. L. Andrés J. (2017). Binding analysis of some classical acetylcholinesterase inhibitors: insights for a rational design using free energy perturbation method calculations with QM/MM MD simulations. J. Chem. Inf. Model. 57, 958–976. 10.1021/acs.jcim.7b00037 28406297

[B32] Neidhardt F. C. Roy C. (1996). Escherichia coli and Salmonella: cellular and molecular biology. Washington, D.C.: ASM Press.

[B33] Omidinia R. Ali Beyramabadi S. Allameh S. Morsali A. Pordel M. (2022). Synthesis, characterization, DFT and antibacterial studies of a novel vitamin B6 Schiff base and its Cu(II) and Zn(II) complexes. J. Mol. Struct. 1248, 131452. 10.1016/j.molstruc.2021.131452

[B34] Patel J. B. Cockerill R. F. Bradford A. P. Eliopoulos M. G. Hindler A. J. Jenkins G. S. (2015). M07-A10: methods for dilution antimicrobial susceptibility tests for bacter.

[B35] Poladian Q. Şahin O. Karakurt T. İlhan-Ceylan B. Kurt Y. (2021). A new zinc(II) complex with N2O2-tetradentate schiff-base derived from pyridoxal-S-methylthiosemicarbazone: synthesis, characterization, crystal structure, DFT, molecular docking and antioxidant activity studies. Polyhedron 201, 115164. 10.1016/j.poly.2021.115164

[B36] Qi J. Zheng Y. Li B. Ai Y. Chen M. Zheng X. (2022). Pyridoxal hydrochloride thiosemicarbazones with copper ions inhibit cell division via Topo-I and Topo-IIɑ. J. Inorg. Biochem. 232, 111816. 10.1016/j.jinorgbio.2022.111816 35405490

[B37] Ramírez-Contreras D. García-García A. Sánchez-Gaytán B. L. Serrano-de la Rosa L. E. Melendez F. J. Choquesillo-Lazarte D. (2022). Bis-citrullinato copper(II) complex: synthesis, crystal structure, and non-covalent interactions. Crystals 12, 1386. 10.3390/cryst12101386

[B38] Salehi M. Ghasemi F. Kubicki M. Asadi A. Behzad M. Ghasemi M. H. (2016). Synthesis, characterization, structural study and antibacterial activity of the Schiff bases derived from sulfanilamides and related copper(II) complexes. Inorganica Chim. Acta 453, 238–246. 10.1016/j.ica.2016.07.028

[B39] Santiago P. H. de O. Duarte E. de A. Nascimento É. C. M. Martins J. B. L. Castro M. S. Gatto C. C. (2022). A binuclear copper(II) complex based on hydrazone ligand: characterization, molecular docking, and theoretical and antimicrobial investigation. Appl. Organomet. Chem. 36, e6461. 10.1002/aoc.6461

[B40] Santiago P. H. O. Santiago M. B. Martins C. H. G. Gatto C. C. (2020). Copper(II) and zinc(II) complexes with Hydrazone: synthesis, crystal structure, Hirshfeld surface and antibacterial activity. Inorganica Chim. Acta 508, 119632. 10.1016/j.ica.2020.119632

[B41] Sarno F. Papulino C. Franci G. Andersen J. H. Cautain B. Melardo C. (2018). 3-Chloro-N′-(2-hydroxybenzylidene) benzohydrazide: an LSD1-selective inhibitor and iron-chelating agent for anticancer therapy. Front. Pharmacol. 9, 1006. 10.3389/fphar.2018.01006 30245629 PMC6137965

[B42] Sharma P. C. Sharma D. Sharma A. Saini N. Goyal R. Ola M. (2020). Hydrazone comprising compounds as promising anti-infective agents: chemistry and structure-property relationship. Mat. Today Chem. 18, 100349. 10.1016/j.mtchem.2020.100349

[B43] Sheldrick G. M. (1997). Program for empirical absorption correction of area detector data.

[B44] Sheldrick G. M. (2008). A short history of*SHELX* . Acta Crystallogr. Sect. A 64, 112–122. 10.1107/s0108767307043930 18156677

[B45] Sheldrick G. M. (2015). *SHELXT*– Integrated space-group and crystal-structure determination. Acta Crystallogr. Sect. C 71, 3–8. 10.1107/s2053273314026370 PMC4283466 25537383

[B46] Shtyrlin N. V. Khaziev R. M. Shtyrlin V. G. Gilyazetdinov E. M. Agafonova M. N. Usachev K. S. (2021). Isonicotinoyl hydrazones of pyridoxine derivatives: synthesis and antimycobacterial activity. Med. Chem. Res. 30, 952–963. 10.1007/s00044-021-02705-w

[B47] Soroceanu A. Bargan A. (2022). Advanced and biomedical applications of schiff-base ligands and their metal complexes: a review. Crystals 12 (10), 1436. 10.3390/cryst12101436

[B48] Spackman M. A. Jayatilaka D. (2009). Hirshfeld surface analysis. CrystEngComm 11, 19–32. 10.1039/b818330a

[B49] Spackman P. R. Turner M. J. McKinnon J. J. Wolff S. K. Grimwood D. J. Jayatilaka D. (2021). *CrystalExplorer*: a program for Hirshfeld surface analysis, visualization and quantitative analysis of molecular crystals. J. Appl. Crystallogr. 54, 1006–1011. 10.1107/s1600576721002910 34188619 PMC8202033

[B50] Trott O. Olson A. J. (2010). AutoDock Vina: improving the speed and accuracy of docking with a new scoring function, efficient optimization, and multithreading. J. Comput. Chem. 31, 455–461. 10.1002/jcc.21334 19499576 PMC3041641

[B51] Vidovic D. Radulovic A. Jevtovic V. (2011). Synthesis, characterization and structural analysis of new copper(II) complexes incorporating a pyridoxal-semicarbazone ligand. Polyhedron 30, 16–21. 10.1016/j.poly.2010.09.022

[B52] Wood P. A. Olsson T. S. G. Cole J. C. Cottrell S. J. Feeder N. Galek P. T. A. (2013). Evaluation of molecular crystal structures using Full Interaction Maps. CrystEngComm 15, 65–72. 10.1039/c2ce25849h

[B53] Xu Y. Shi Y. Lei F. Dai L. (2020). A novel and green cellulose-based Schiff base-Cu (II) complex and its excellent antibacterial activity. Carbohydr. Polym. 230, 115671. 10.1016/j.carbpol.2019.115671 31887926

[B54] Yan Y.-B. Yang R.-W. Zhang H.-W. Zhang Y. Dong W.-K. (2024). Crystal structure and luminescent mechanochromism of a quinoline-appended acylhydrazone ligand and its Zn(II) complex. J. Mol. Struct. 1299, 137148. 10.1016/j.molstruc.2023.137148

[B55] Yilmaz S. K. Agar A. A. Cinar E. B. Dege N. Vidya V. G. Viju Kumar V. G. (2024). Deciphering non-covalent interactions in unprecedented binuclear copper complex: spectroscopic, Hirshfeld surface and DFT investigation. J. Mol. Struct. 1299, 137111. 10.1016/j.molstruc.2023.137111

[B56] Yuan J. Song J.-Y. Yang H.-H. Lan H.-R. Xing A.-P. Li K.-H. (2023). Synthesis, cytotoxicity and DNA binding of novel Ni(II), Co(II) and Zn(II) complexes bearing pyrimidinyl hydrazone ligand. Mol. Struct. 1276, 134724. 10.1016/j.molstruc.2022.134724

[B57] Ziervogel B. K. Roux B. (2013). The binding of antibiotics in OmpF porin. Structure 21, 76–87. 10.1016/j.str.2012.10.014 23201272 PMC3545085

