# Supplementary material for: Effects of changing ions on the crystal design, non-covalent interactions, antimicrobial activity, and molecular docking of Cu(II) complexes with a pyridoxal-hydrazone ligand
Source: Front Chem. 2024 Feb 1;12:1347370. doi: 10.3389/fchem.2024.1347370 (PMC10867249; doi:10.3389/fchem.2024.1347370)

checkCIF/PLATON report

Structure factors have been supplied for datablock(s) ccg_fcl_cu5_0m

THIS REPORT IS FOR GUIDANCE ONLY. IF USED AS PART OF A REVIEW PROCEDURE FOR PUBLICATION, IT SHOULD NOT REPLACE THE EXPERTISE OF AN EXPERIENCED CRYSTALLOGRAPHIC REFEREE.

No syntax errors found. CIF dictionary Interpreting this report

Datablock: ccg_fcl_cu5_0m

Bond precision: C-C = 0.0052 A Wavelength=0.71073

Cell:	a=7.6431(17)	b=18.955(4)	c=12.926(3)
	alpha=90	beta=96.112(5)	gamma=90
Temperature:	273 K		

	Calculated	Reported
Volume	1862.0(7)	1862.1(7)
Space group	P 21/c	P 1 21/c 1
Hall group	-P 2ybc	-P 2ybc
Moiety formula	C15 H15 Cl2 Cu N3 O3 [+ solvent]	C15 H15 Cl2 Cu N3 O3
Sum formula	C15 H15 Cl2 Cu N3 O3 [+ solvent]	C15 H15 Cl2 Cu N3 O3
Mr	419.75	419.74
Dx,g cm-3	1.497	1.497
Z	4	4
Mu (mm-1)	1.477	1.477
F000	852.0	852.0
F000'	854.72	
h,k,lmax	9,22,15	9,22,15
Nref	3445	3440
Tmin,Tmax	0.737,0.806	0.740,0.810
Tmin'	0.723	

Correction method= # Reported T Limits: Tmin=0.740 Tmax=0.810
AbsCorr = MULTI-SCAN

Data completeness= 0.999 Theta(max)= 25.415

R(reflections)= 0.0391(2385) wR2(reflections)= 0.1064(3440)

S = 1.035 Npar= 224

The following ALERTS were generated. Each ALERT has the format
test-name_ALERT_alert-type_alert-level.
Click on the hyperlinks for more details of the test.

Alert level B

```
PLAT420_ALERT_2_B D-H Bond Without Acceptor  O1       --H1A      .     Please Check
```

Alert level C

```
PLAT222_ALERT_3_C NonSolvent Resd 1  H   Uiso(max)/Uiso(min) Range        9.6 Ratio
PLAT245_ALERT_2_C U(iso) H3        Smaller than U(eq) N3        by      0.020 Ang**2
PLAT352_ALERT_3_C Short  N-H (X0.87,N1.01A)  N3       - H3       .      0.70 Ang.
PLAT905_ALERT_3_C Negative K value in the Analysis of Variance ...     -1.037 Report
```

Alert level G

```
PLAT007_ALERT_5_G Number of Unrefined Donor-H Atoms .............          2 Report
PLAT066_ALERT_1_G Predicted and Reported Tmin&Tmax Range Identical          ? Check
PLAT199_ALERT_1_G Reported _cell_measurement_temperature ..... (K)        273 Check
PLAT200_ALERT_1_G Reported   _diffrn_ambient_temperature ..... (K)        273 Check
PLAT232_ALERT_2_G Hirshfeld Test Diff (M-X)  Cu1      --Cl2      .        6.0 s.u.
PLAT480_ALERT_4_G Long H...A H-Bond Reported H8       ..CL2      .       2.95 Ang.
PLAT480_ALERT_4_G Long H...A H-Bond Reported H5C      ..CL1      .       2.91 Ang.
PLAT605_ALERT_4_G Largest Solvent Accessible VOID in the Structure        141 A**3
PLAT794_ALERT_5_G Tentative Bond Valency for Cu1       (II)      .       2.25 Info
PLAT868_ALERT_4_G ALERTS Due to the Use of _smtbx_masks Suppressed          ! Info
PLAT912_ALERT_4_G Missing # of FCF Reflections Above STh/L=  0.600          5 Note
PLAT978_ALERT_2_G Number C-C Bonds with Positive Residual Density.          3 Info
```

---

0 **ALERT level A** = Most likely a serious problem - resolve or explain
1 **ALERT level B** = A potentially serious problem, consider carefully
4 **ALERT level C** = Check. Ensure it is not caused by an omission or oversight
12 **ALERT level G** = General information/check it is not something unexpected

3 ALERT type 1 CIF construction/syntax error, inconsistent or missing data
4 ALERT type 2 Indicator that the structure model may be wrong or deficient
3 ALERT type 3 Indicator that the structure quality may be low
5 ALERT type 4 Improvement, methodology, query or suggestion
2 ALERT type 5 Informative message, check

It is advisable to attempt to resolve as many as possible of the alerts in all categories. Often the minor alerts point to easily fixed oversights, errors and omissions in your CIF or refinement strategy, so attention to these fine details can be worthwhile. In order to resolve some of the more serious problems it may be necessary to carry out additional measurements or structure refinements. However, the purpose of your study may justify the reported deviations and the more serious of these should normally be commented upon in the discussion or experimental section of a paper or in the "special_details" fields of the CIF. checkCIF was carefully designed to identify outliers and unusual parameters, but every test has its limitations and alerts that are not important in a particular case may appear. Conversely, the absence of alerts does not guarantee there are no aspects of the results needing attention. It is up to the individual to critically assess their own results and, if necessary, seek expert advice.

Publication of your CIF in IUCr journals

A basic structural check has been run on your CIF. These basic checks will be run on all CIFs submitted for publication in IUCr journals (*Acta Crystallographica*, *Journal of Applied Crystallography*, *Journal of Synchrotron Radiation*); however, if you intend to submit to *Acta Crystallographica Section C* or *E* or *IUCrData*, you should make sure that full publication checks are run on the final version of your CIF prior to submission.

Publication of your CIF in other journals

Please refer to the *Notes for Authors* of the relevant journal for any special instructions relating to CIF submission.

PLATON version of 06/07/2023; check.def file version of 30/06/2023

Datablock ccg_fcl_cu5_0m - ellipsoid plot

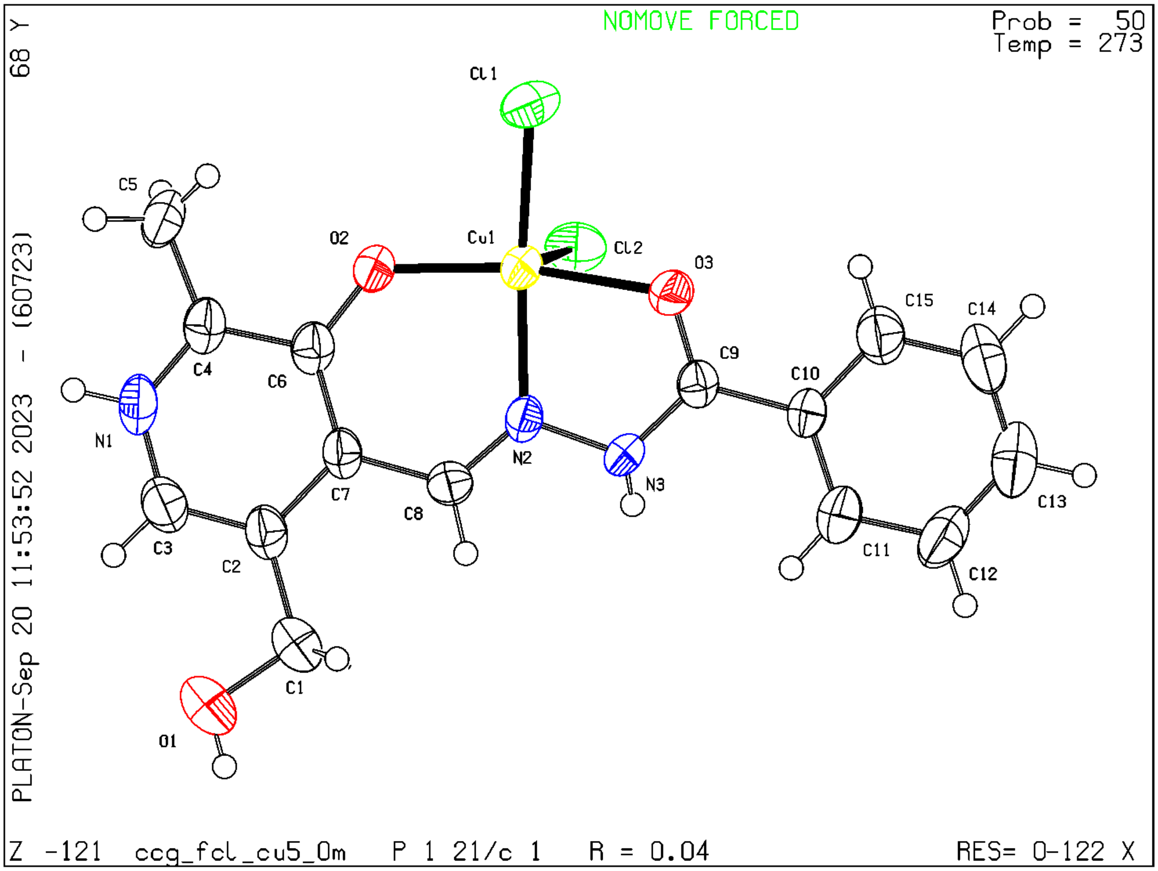

Supplement: Supplementary file 1 [file DataSheet1.ZIP › checkcif(1).pdf]
