# Supplementary material for: Effects of changing ions on the crystal design, non-covalent interactions, antimicrobial activity, and molecular docking of Cu(II) complexes with a pyridoxal-hydrazone ligand
Source: Front Chem. 2024 Feb 1;12:1347370. doi: 10.3389/fchem.2024.1347370 (PMC10867249; doi:10.3389/fchem.2024.1347370)

checkCIF/PLATON report

Structure factors have been supplied for datablock(s) shelx

THIS REPORT IS FOR GUIDANCE ONLY. IF USED AS PART OF A REVIEW PROCEDURE FOR PUBLICATION, IT SHOULD NOT REPLACE THE EXPERTISE OF AN EXPERIENCED CRYSTALLOGRAPHIC REFEREE.

No syntax errors found. CIF dictionary Interpreting this report

Datablock: shelx

Bond precision: C-C = 0.0036 A Wavelength=0.71073

Cell:	a=9.5137(13)	b=15.720(2)	c=12.8249(17)
	alpha=90	beta=100.198(2)	gamma=90
Temperature:	273 K		

	Calculated	Reported
Volume	1887.7(4)	1887.8(4)
Space group	P 21/n	P 1 21/n 1
Hall group	-P 2yn	-P 2yn
Moiety formula	C15 H17 Cl Cu N3 O4, N O3, H2 O	C15 H17 Cl Cu N3 O4, H2 O, N O3
Sum formula	C15 H19 Cl Cu N4 O8	C15 H19 Cl Cu N4 O8
Mr	482.34	482.33
Dx,g cm-3	1.697	1.697
Z	4	4
Mu (mm-1)	1.352	1.352
F000	988.0	988.0
F000'	990.32	
h,k,lmax	11,19,15	11,18,15
Nref	3490	3485
Tmin,Tmax	0.611,0.826	0.750,0.830
Tmin'	0.572	

Correction method= # Reported T Limits: Tmin=0.750 Tmax=0.830
AbsCorr = MULTI-SCAN

Data completeness= 0.999 Theta(max)= 25.438

R(reflections)= 0.0298(2811) wR2(reflections)= 0.0776(3485)

S = 1.043 Npar= 273

The following ALERTS were generated. Each ALERT has the format
test-name_ALERT_alert-type_alert-level.
Click on the hyperlinks for more details of the test.

Alert level C

```
PLAT031_ALERT_4_C Refined Extinction Parameter Within Range of ...      2.667 Sigma
PLAT042_ALERT_1_C Calc. and Reported MoietyFormula Strings  Differ     Please Check
PLAT244_ALERT_4_C Low    'Solvent' Ueq as Compared to Neighbors of         N4 Check
```

Alert level G

```
PLAT007_ALERT_5_G Number of Unrefined Donor-H Atoms .............           6 Report
PLAT199_ALERT_1_G Reported _cell_measurement_temperature ..... (K)        273 Check
PLAT200_ALERT_1_G Reported   _diffrn_ambient_temperature ..... (K)        273 Check
PLAT480_ALERT_4_G Long H...A H-Bond Reported H4A      ..N4       .       2.69 Ang.
PLAT480_ALERT_4_G Long H...A H-Bond Reported H4A      ..N4       .       2.69 Ang.
PLAT790_ALERT_4_G Centre of Gravity not Within Unit Cell: Resd.  #          2 Note
              N O3
PLAT794_ALERT_5_G Tentative Bond Valency for Cu1       (II)      .       2.27 Info
PLAT912_ALERT_4_G Missing # of FCF Reflections Above STh/L=  0.600          7 Note
PLAT978_ALERT_2_G Number C-C Bonds with Positive Residual Density.          6 Info
```

0 **ALERT level A** = Most likely a serious problem - resolve or explain
0 **ALERT level B** = A potentially serious problem, consider carefully
3 **ALERT level C** = Check. Ensure it is not caused by an omission or oversight
9 **ALERT level G** = General information/check it is not something unexpected

3 ALERT type 1 CIF construction/syntax error, inconsistent or missing data
1 ALERT type 2 Indicator that the structure model may be wrong or deficient
0 ALERT type 3 Indicator that the structure quality may be low
6 ALERT type 4 Improvement, methodology, query or suggestion
2 ALERT type 5 Informative message, check

It is advisable to attempt to resolve as many as possible of the alerts in all categories. Often the minor alerts point to easily fixed oversights, errors and omissions in your CIF or refinement strategy, so attention to these fine details can be worthwhile. In order to resolve some of the more serious problems it may be necessary to carry out additional measurements or structure refinements. However, the purpose of your study may justify the reported deviations and the more serious of these should normally be commented upon in the discussion or experimental section of a paper or in the "special_details" fields of the CIF. checkCIF was carefully designed to identify outliers and unusual parameters, but every test has its limitations and alerts that are not important in a particular case may appear. Conversely, the absence of alerts does not guarantee there are no aspects of the results needing attention. It is up to the individual to critically assess their own results and, if necessary, seek expert advice.

Publication of your CIF in IUCr journals

A basic structural check has been run on your CIF. These basic checks will be run on all CIFs submitted for publication in IUCr journals (*Acta Crystallographica*, *Journal of Applied Crystallography*, *Journal of Synchrotron Radiation*); however, if you intend to submit to *Acta Crystallographica Section C* or *E* or *IUCrData*, you should make sure that full publication checks are run on the final version of your CIF prior to submission.

Publication of your CIF in other journals

Please refer to the *Notes for Authors* of the relevant journal for any special instructions relating to CIF submission.

PLATON version of 06/07/2023; check.def file version of 30/06/2023

Datablock shelx - ellipsoid plot

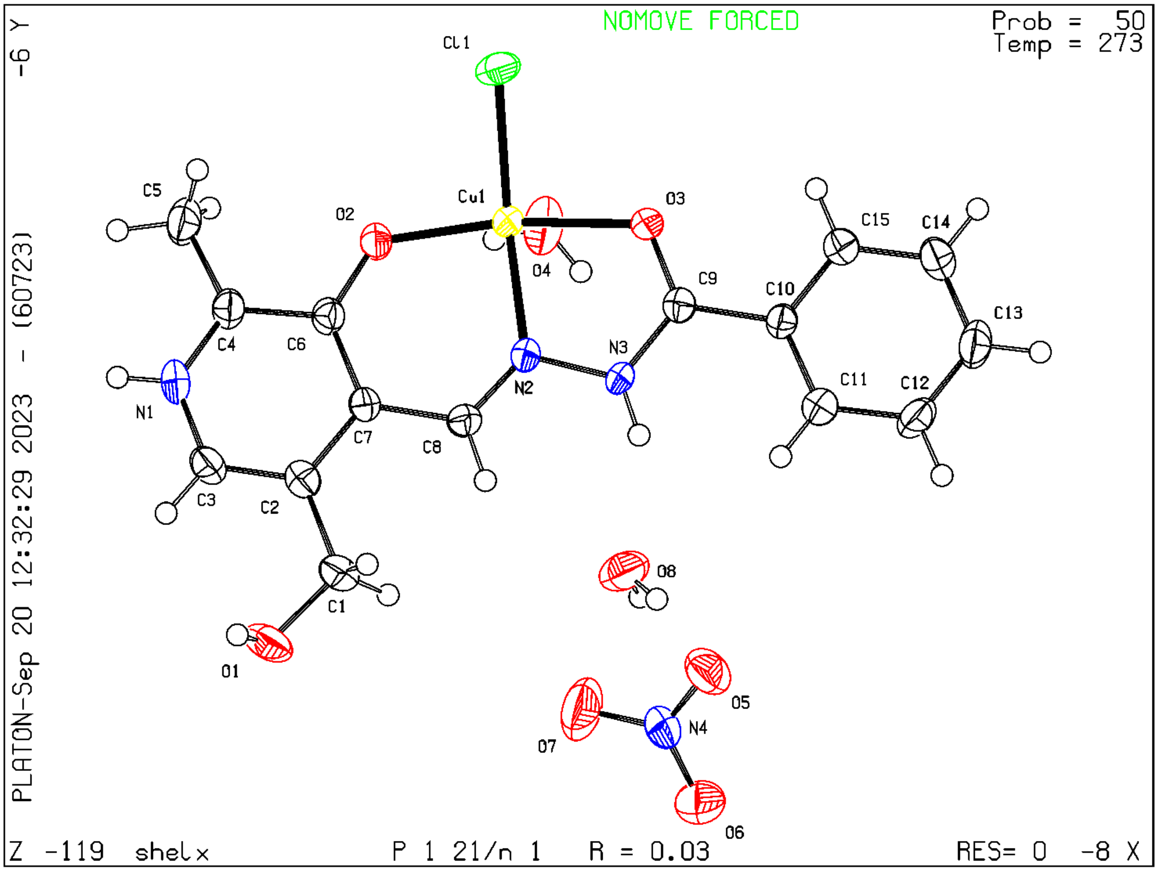

Supplement: Supplementary file 1 [file DataSheet1.ZIP › checkcif(3).pdf]
