# Supplementary material for: Effects of changing ions on the crystal design, non-covalent interactions, antimicrobial activity, and molecular docking of Cu(II) complexes with a pyridoxal-hydrazone ligand
Source: Front Chem. 2024 Feb 1;12:1347370. doi: 10.3389/fchem.2024.1347370 (PMC10867249; doi:10.3389/fchem.2024.1347370)
Supplement: Supplementary file 1 [file DataSheet1.ZIP › Photograph of the biological test.pdf]

Biological activity analysis

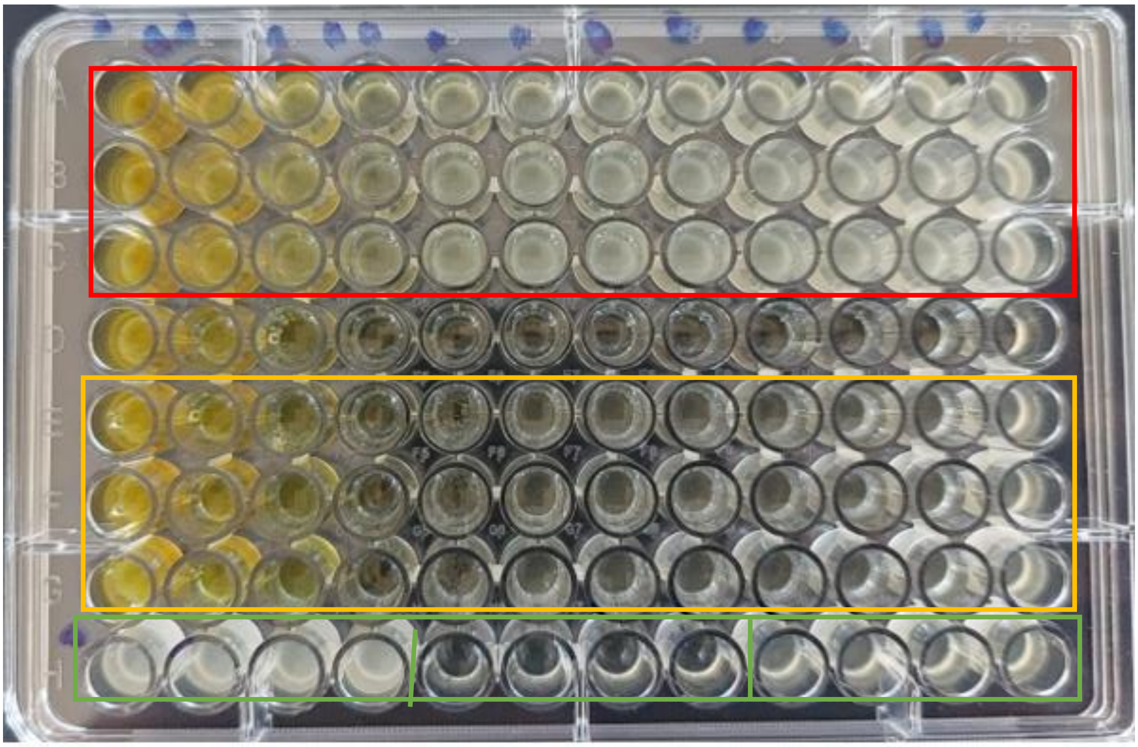

Figure 1. Photograph of the biological test of the complex (1).

- *Escherichia coli*
- *Staphylococcus aureus*
- Growth control (negative) *E. coli*, pure MH broth and Growth control (negative) *S. aureus*. Line D: White (broth with the compound).

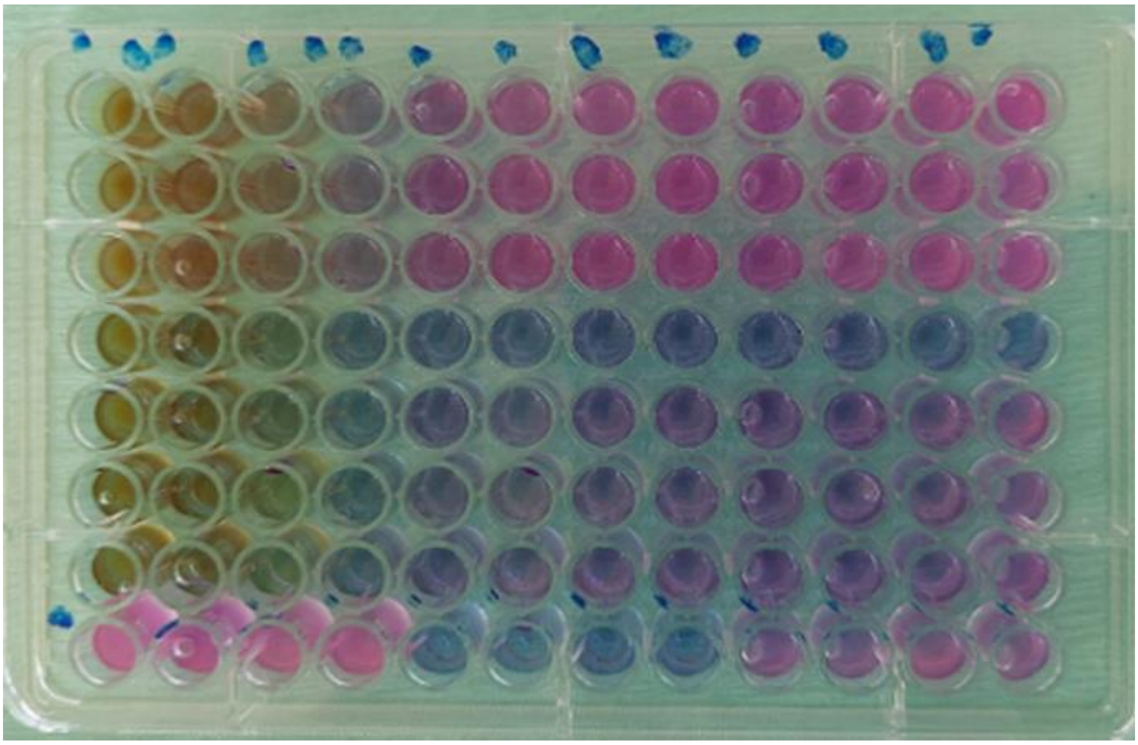

Figure 2. Photograph (*Escherichia coli and Staphylococus aures*) of the plate with resazurin of the complex (1). Pink indicates cellular viability and blue colors or shades of green or yellow indicate cell death.

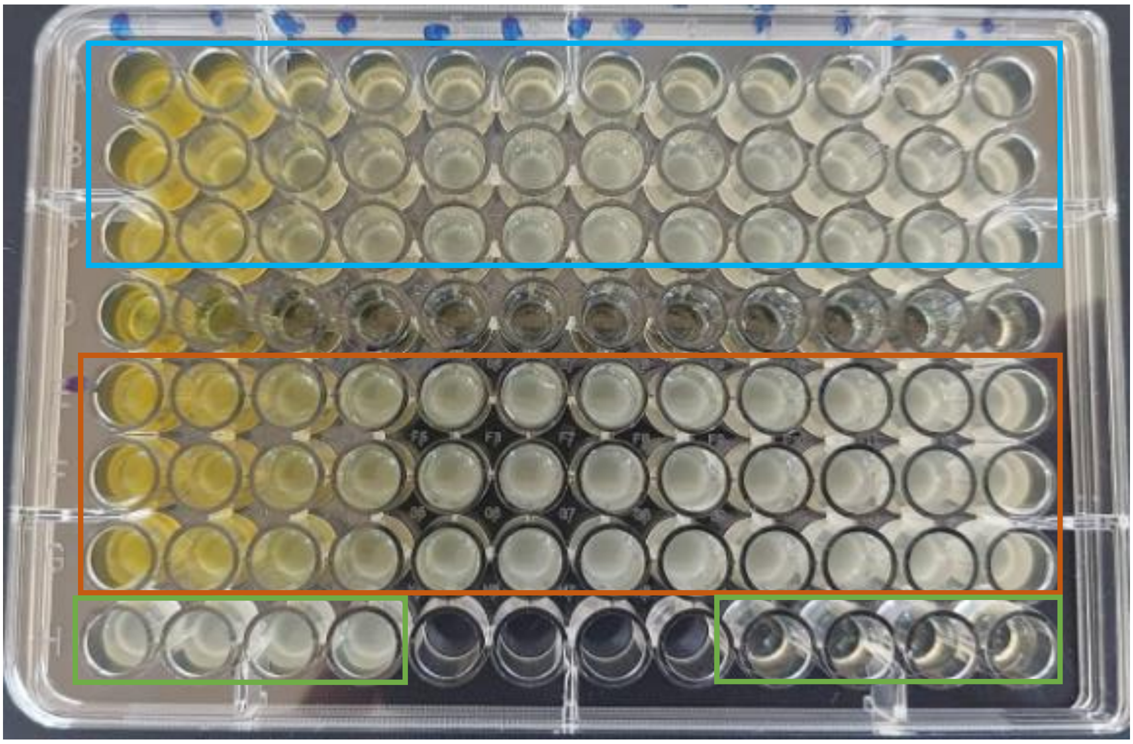

Figure 3. Photograph of the biological test with *Escherichia coli* of the complexes (2) and (3).

Complex (3)

Complex (2)

Growth control (negative) *E. coli*, pure MH broth. Line D: White (broth with complex 3).

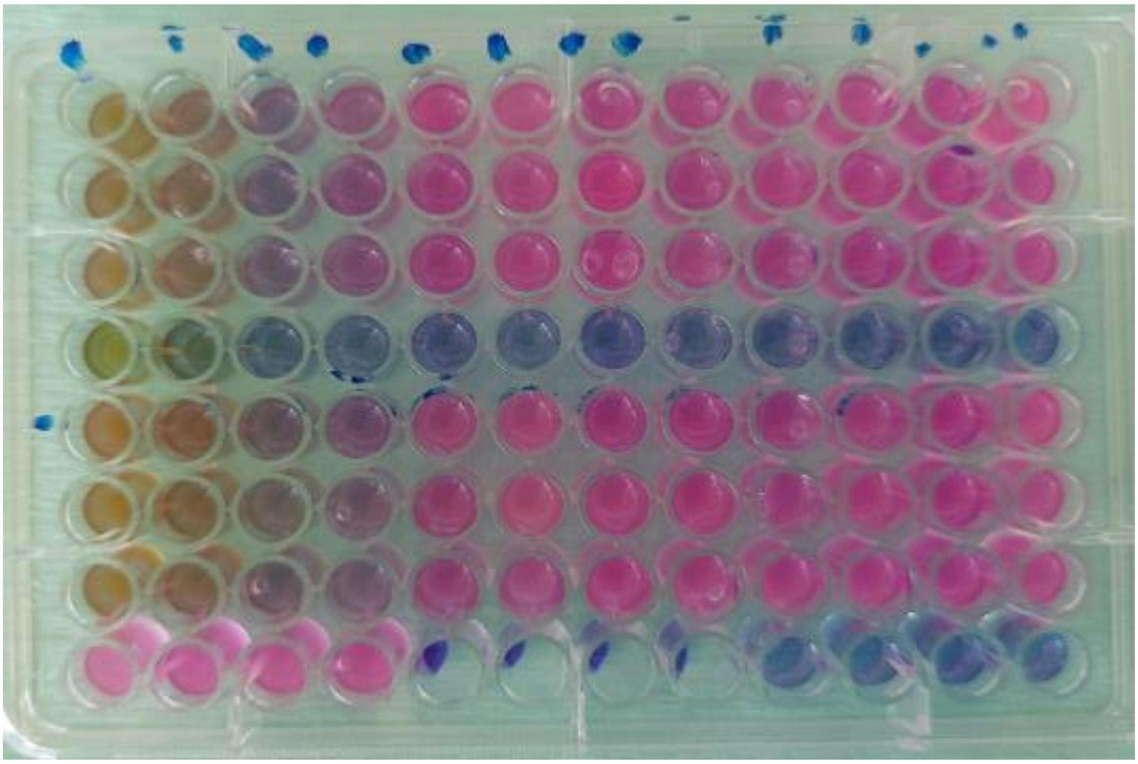

Figure 4. Photograph (*Escherichia coli*) of the plate with resazurin of the complexes (2) and (3). Pink indicates cellular viability and blue colors or shades of green or yellow indicate cell death.

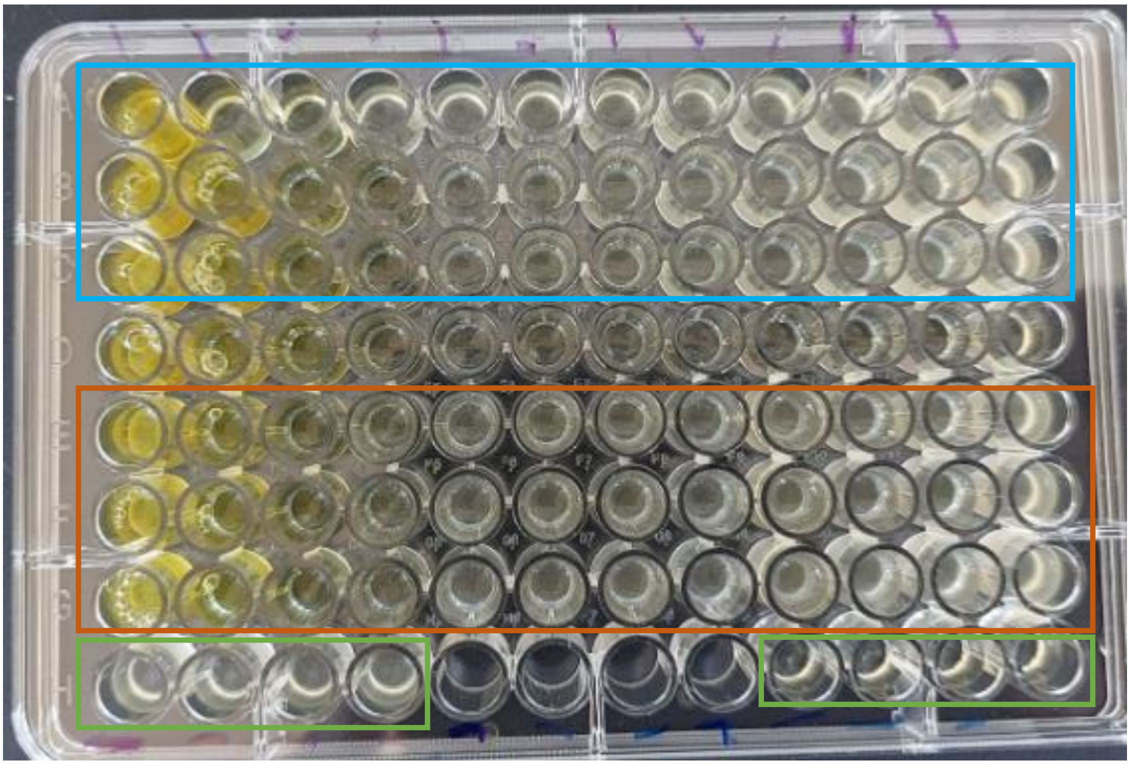

Figure 5. Photograph of the biological test with *Staphylococus aures* of the complexes (2) and (3).

Complex 3

Complex 2

Growth control (negative) *Staphylococus aures*, pure MH broth. Line D: White (broth with complex 3).

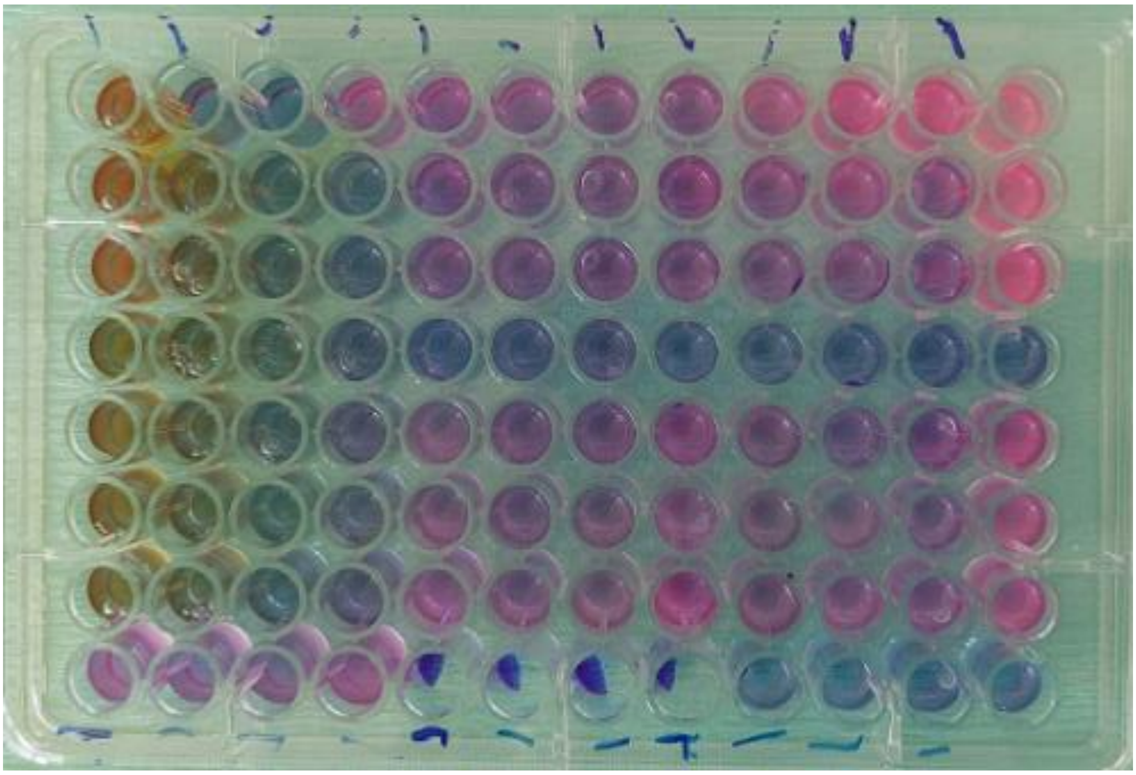

Figure 6. Photograph (*Staphylococus aures)* of the plate with resazurin of the complexes (2) and (3). Pink indicates cellular viability and blue colors or shades of green or yellow indicate cell death.
