# Supplementary material for: Effects of changing ions on the crystal design, non-covalent interactions, antimicrobial activity, and molecular docking of Cu(II) complexes with a pyridoxal-hydrazone ligand
Source: Front Chem. 2024 Feb 1;12:1347370. doi: 10.3389/fchem.2024.1347370 (PMC10867249; doi:10.3389/fchem.2024.1347370)
Supplement: Supplementary file 1 [file DataSheet1.ZIP › Supplementary_Material_26-12-23.pdf]

Supplementary Material

Effects of Changing Ions on the Crystal Design, Noncovalent Interactions, Antimicrobial Activity and Molecular Docking of Cu(II) Complexes with a Pyridoxal-Hydrazone Ligand

Claudia C. Gatto,[a,] Lucas M. Dias,[a] Clarisse A. Paiva,[a] Izabel C. R. da Silva,[b] Daniel O. Freire,[b] Renata P. I. Tormena[b], Érica C. M. Nascimento[c] and João B. L. Martins[c]*

[a]University of Brasilia, Institute of Chemistry, Laboratory of Inorganic Synthesis and Crystallography, Brasília-DF, Brazil.

[b]University of Brasilia, Faculty UnB Ceilândia, Graduate Program in Health Sciences and Technologies, Brasília-DF, Brazil.

[c]University of Brasilia, Institute of Chemistry, Laboratory of Computational Chemistry, Brasília-DF, Brazil.

*Correspondence: Claudia C. Gatto (e-mail address: ccgatto@gmail.com; ORCID: https://orcid.org/0000-0002-3736-6861) – University of Brasilia (IQ-UnB), LASIC - Laboratory of Inorganic Synthesis and Crystallography, CEP 70904-970, Brasília-DF, Brazil; Tel.: 55 61 31073872; fax: 55 61 31073900.

SUMMARY

Figure S1. Projection view of the complex (1) showing the π···π stacking interactions (as a dashed line)……………………………………………….……………………...……………………….3
Figure S2. Projection view of the complex (2) showing the π···π stacking interactions (as a dashed line)……………………………………………….…………………...……………………….3
Figure S3. Projection view of the complex (3) showing the π···π stacking interactions (as a dashed line)……………………………………………….…………………...……..........................4
Figure S4. Projection view of the complex (4) showing the π···π stacking interactions (as a dashed line)……………………………………………....………...………………………4
Table S1. Hydrogen bonds found in the complexes (1-4)………………………..……….……5
Figure S5. ^{1}H-NMR spectra of PLBHZ…………………….………….…………………7
Figure S6. ^{13}C-NMR spectra of PLBHZ……………….…………………...………………7
Figure S7. IR spectra of PLBHZ……………………………….………….……………………8
Figure S8. IR spectra of compound (1) ……………………..………….………………………8
Figure S9. IR spectra of compound (2) ……………………..………….………………………9
Figure S10. IR spectra of compound (3) ………………….…..……….……………………9
Figure S11. IR spectra of compound (4) …………………...……..……..………...……………10
Figure S12. UV-vis spectra of compound PLBHZ in MeOH …….…………………...…….10
Figure S13. UV-vis spectra of compound (1-4) in MeOH ………………………………….....11
Figure S14. UV-vis spectra of compound (1-4) in DMF…………………….…………………..11
Table S2. Results obtained in the electron spectroscopy with wavelength values in nm……………………………….………………………………………………………...….12
Figure S15. UV-vis spectra of compound (1-4) in DMF (d-d transition) ……………………..12
Table S3. Results obtained in the electron spectroscopy (d-d transition) with wavelength values in nm……………………………….………………………………………………………...….12
Figure S16. ESI(+)-MSMS of PLBHZ…………………………………………………………..13
Figure S17. ESI(+)-MSMS of (1)……………………………………………...……………...13
Figure S18. ESI(+)-MSMS of (2)…………………………………...………………………...14
Figure S19. ESI(+)-MSMS of (3)……………………………………………………………..14
Figure S20. ESI(+)-MSMS of (4)……………………………………………………………..15
Figure S21. Hirshfeld surface mapped in *shape index* for (1-4)……...……………………...…...15
Figure S22. Fingerprint plots for (1)……………………………………………………………16
Figure S23. Fingerprint plots for (2)……………………………………………………………16
Figure S24. Fingerprint plots for (3)……………………………………………………………17
Figure S25. Fingerprint plots for (4)……………………………………………………………17
Figure S26. Superposing of best score pose of the redocking study. (a) MecR1-oxalloyl (**PDB** 2IWD). (b) OmpF/A-ampicillin (**PDB** 24GCP)…..……………………..…………………………18
Figure S27. Superposing of best score pose of the docking study of the complex MecR1-ligands (**PDB** 2IWD). Molecule/color: Oxacillin (ball and stick)/blue; Oxacillin/yellow, redocked/orange; PLBHZ (ball and stick)/red; (1)/green; (2)/garnet; (3)/light green; (4)/pink…….……………………18
Figure S28. Superposing of best score pose of the docking study of the complex OmpF/A-ligands (**PDB** 4GCP). Molecule/color: ampicillin (ball and stick)/blue; PLBHZ (ball and stick)/red; (1)/green; (2)/garnet; (3)/light green; (4)/pink…..……………………………………………19

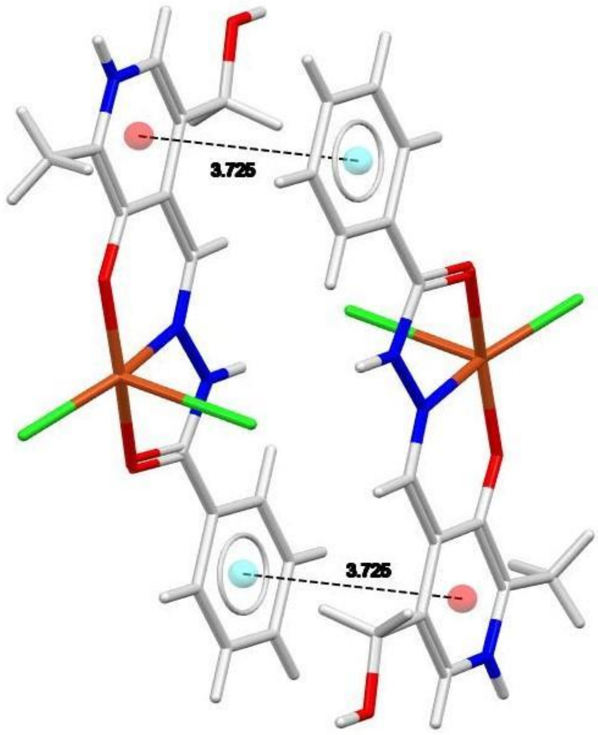

Figure S1. Projection view of the complex (1) showing the π···π stacking interactions (as a dashed line).

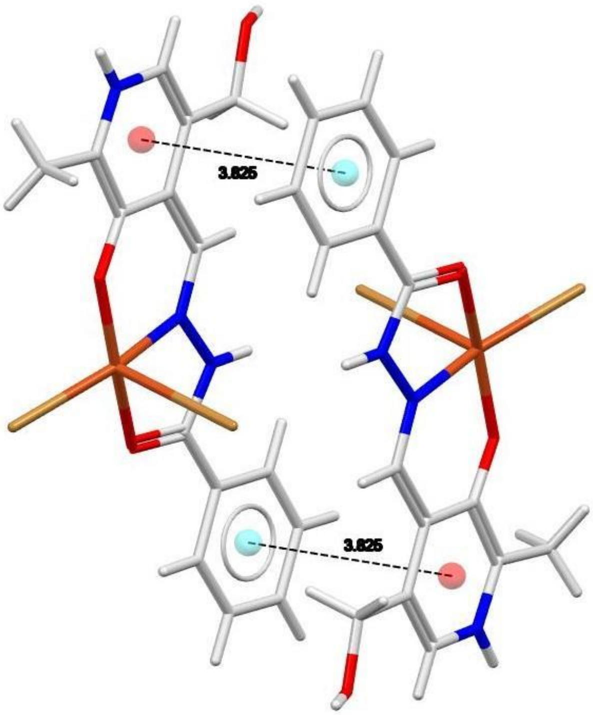

Figure S2. Projection view of the complex (2) showing the π···π stacking interactions (as a dashed line).

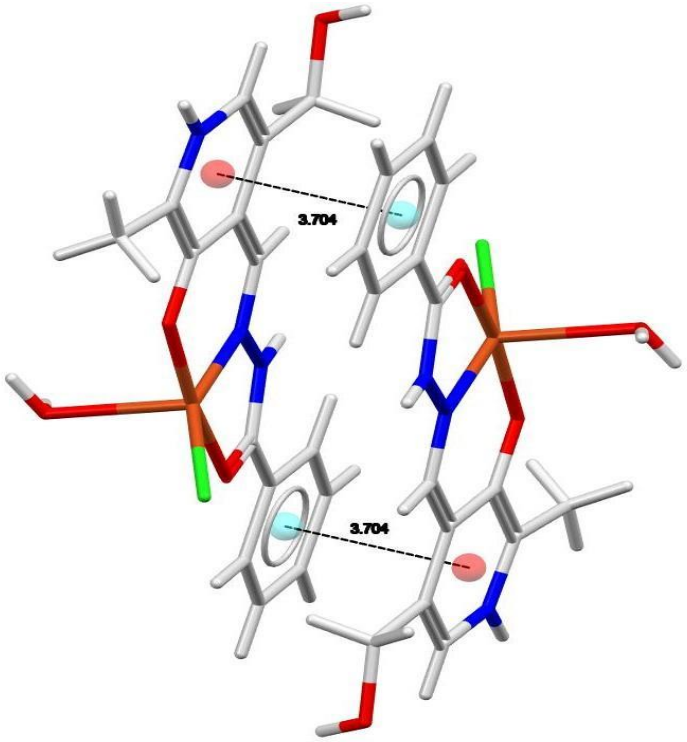

Figure S3. Projection view of the complex (3) showing the π···π stacking interactions (as a dashed line).

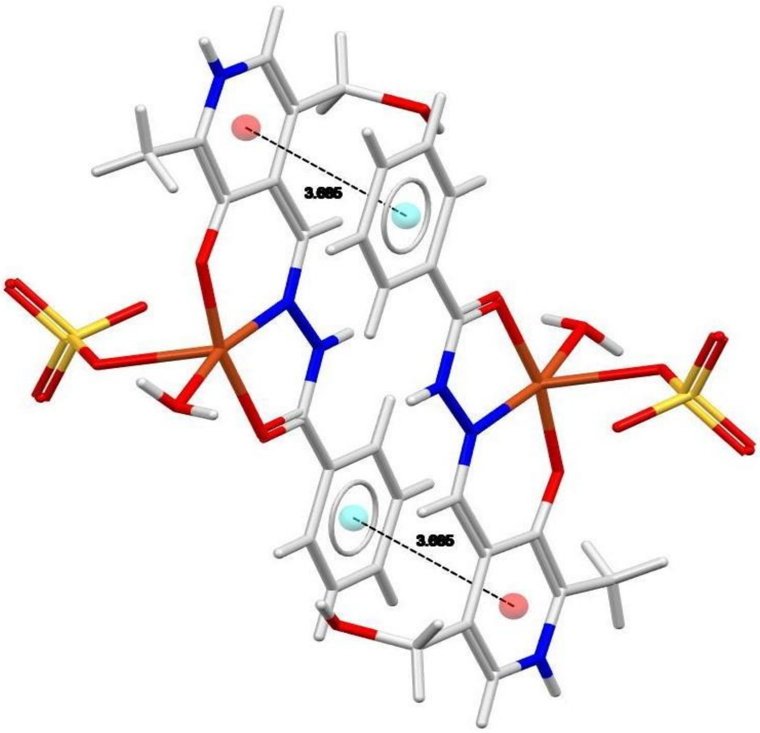

Figure S4. Projection view of the complex (4) showing the π···π stacking interactions (as a dashed line).

Table S1. Hydrogen bonds found in the complexes (1-4).

Complex (1)

D-H...A	d(D-H)	d(H...A)	d(D...A)	<(DHA)
N(3)-H(3)...Cl(2)#1	0.86	2.35	3.175(3)	160.2
N(1)-H(1)...Cl(1)#2	0.86	2.45	3.262(3)	158.3
C(8)-H(8)...Cl(2)#1	0.93	2.95	3.697(4)	138.0
C(5)-H(5A)...O(1)#3	0.96	2.42	3.360(6)	167.1
C(5)-H(5C)...Cl(1)#2	0.96	2.91	3.764(4)	148.4

Symmetry transformations used to generate equivalent atoms: #1 -x, -y+1, -z+1 #2 x, -y+1/2, z-1/2 #3 x-y+1/2, z+1/2.

Complex (2)

D-H...A	d(D-H)	d(H...A)	d(D...A)	<(DHA)
N(3)-H(3)...Br(2)#1	0.86	2.50	3.327(6)	161.7
C(8)-H(8)...Br(2)#1	0.93	3.02	3.779(7)	140.1
N(1)-H(1)...Br(1)#2	0.86	2.58	3.380(6)	155.6
C(5)-H(5B)...Br(1)#2	0.96	2.98	3.843(9)	149.8
C(5)-H(5C)...O(1)#3	0.96	2.43	3.367(13)	163.8

Symmetry transformations used to generate equivalent atoms: #1 -x+2,-y+1,-z+1 #2 x,-y+1/2,z+1/2 #3 x,-y+1/2,z-1/2.

Complex (3)

O(1)-H(1)...O(6)#1	0.82	2.43	3.066(3)	135.4
O(1)-H(1)...N(4)#1	0.82	2.54	3.335(3)	164.3
O(1)-H(1)...O(7)#1	0.82	2.00	2.805(3)	165.7
O(4)-H(4A)...O(6)#2	0.85	2.06	2.871(3)	158.7
O(4)-H(4A)...N(4)#2	0.85	2.69	3.355(3)	135.6
O(4)-H(4B)...O(1)#3	0.85	2.05	2.879(3)	163.4
N(3)-H(3)...O(8)	0.86	1.99	2.755(3)	148.2
C(8)-H(8)...O(8)	0.93	2.42	3.135(3)	133.5
C(3)-H(3A)...O(5)#4	0.93	2.54	3.039(3)	114.3
C(5)-H(5C)...O(4)#5	0.96	2.48	3.379(3)	155.5
N(1)-H(1A)...O(5)#4	0.83(3)	2.31(3)	2.906(3)	129(3)
O(1)-H(1)...O(6)#1	0.82	2.43	3.066(3)	135.4
O(1)-H(1)...N(4)#1	0.82	2.54	3.335(3)	164.3
O(1)-H(1)...O(7)#1	0.82	2.00	2.805(3)	165.7
O(4)-H(4A)...O(6)#2	0.85	2.06	2.871(3)	158.7

O(4)-H(4A)...N(4)#2	0.85	2.69	3.355(3)	135.6
O(4)-H(4B)...O(1)#3	0.85	2.05	2.879(3)	163.4
N(3)-H(3)...O(8)	0.86	1.99	2.755(3)	148.2
C(8)-H(8)...O(8)	0.93	2.42	3.135(3)	133.5
C(3)-H(3A)...O(5)#4	0.93	2.54	3.039(3)	114.3
C(5)-H(5C)...O(4)#5	0.96	2.48	3.379(3)	155.5
N(1)-H(1A)...O(5)#4	0.83(3)	2.31(3)	2.906(3)	129(3)
Symmetry transformations used to generate equivalent atoms: #1 x+1/2,-y+3/2,z+1/2 #2 x+1,y,z #3 -x+1/2,y-1/2,-z+3/2 #4 -x+1/2,y+1/2,-z+3/2 #5 -x+3/2,y+1/2,-z+3/2.				
**Complex (4)**				
O(4)-H(4A)...S(1)#1	0.85	2.88	3.6503(19)	151.8
O(4)-H(4A)...O(8)#1	0.85	1.80	2.638(3)	169.6
O(4)-H(4B)...O(10)	0.85	1.97	2.804(3)	165.4
O(1)-H(1)...O(7)#2	0.82	1.88	2.685(3)	167.8
N(3)-H(3)...S(1)#2	0.86	3.02	3.836(2)	159.8
N(3)-H(3)...O(6)#2	0.86	1.89	2.617(3)	141.8
N(1)-H(1A)...S(1)#3	0.86	2.79	3.497(2)	141.1
N(1)-H(1A)...O(5)#3	0.86	1.85	2.686(3)	163.6
C(8)-H(8)...O(1)	0.93	2.58	3.134(3)	118.7
C(8)-H(8)...O(6)#2	0.93	2.33	3.023(3)	130.7
C(5)-H(5A)...O(3)#3	0.96	2.56	3.475(4)	159.4
Symmetry transformations used to generate equivalent atoms: #1 x+1,y,z #2 -x+1,-y+1,-z+1 #3 x,-y+1/2,z+1/2.				

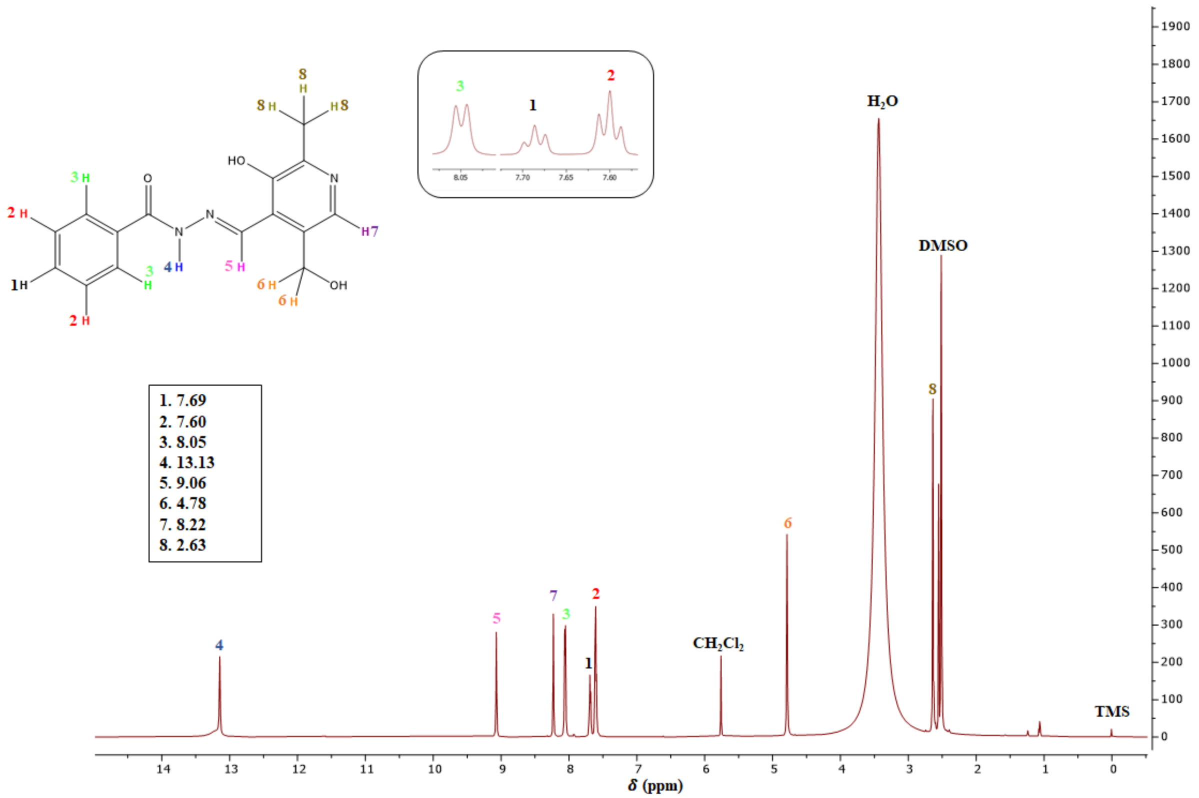

Figure S5. ^{1}H-NMR spectra of PLBHZ.

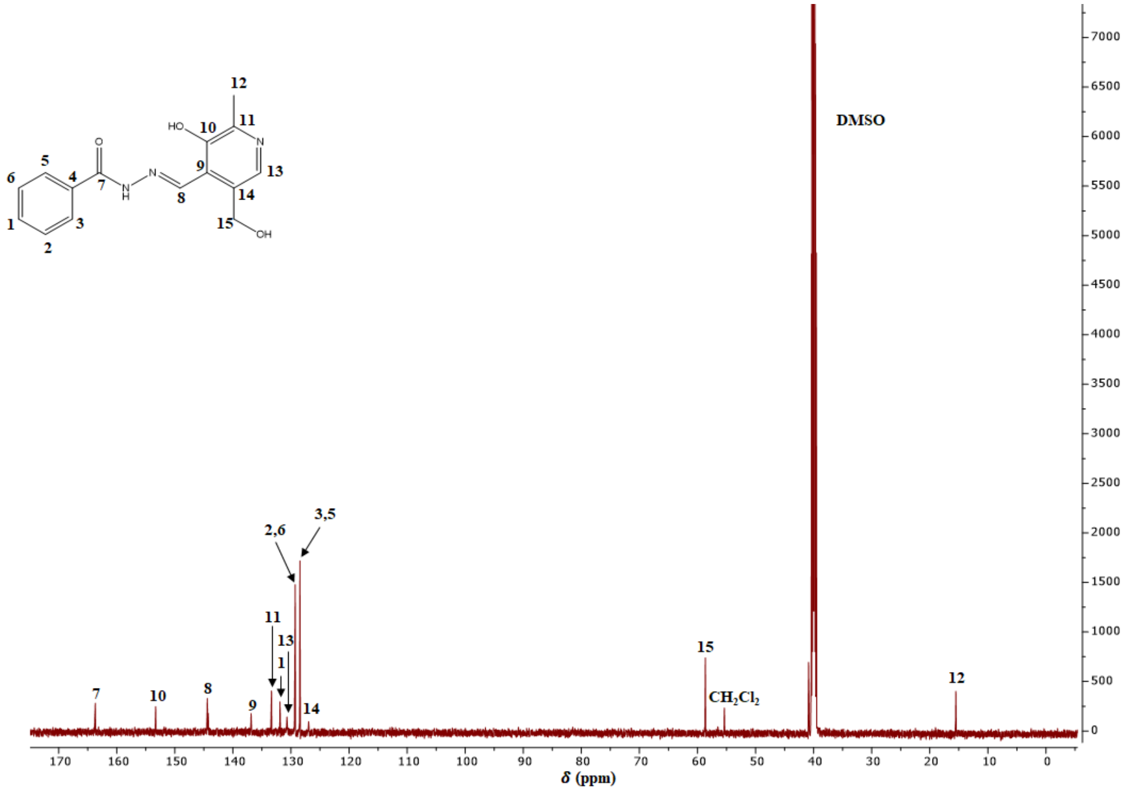

Figure S6. ^{13}C-NMR spectra of PLBHZ.

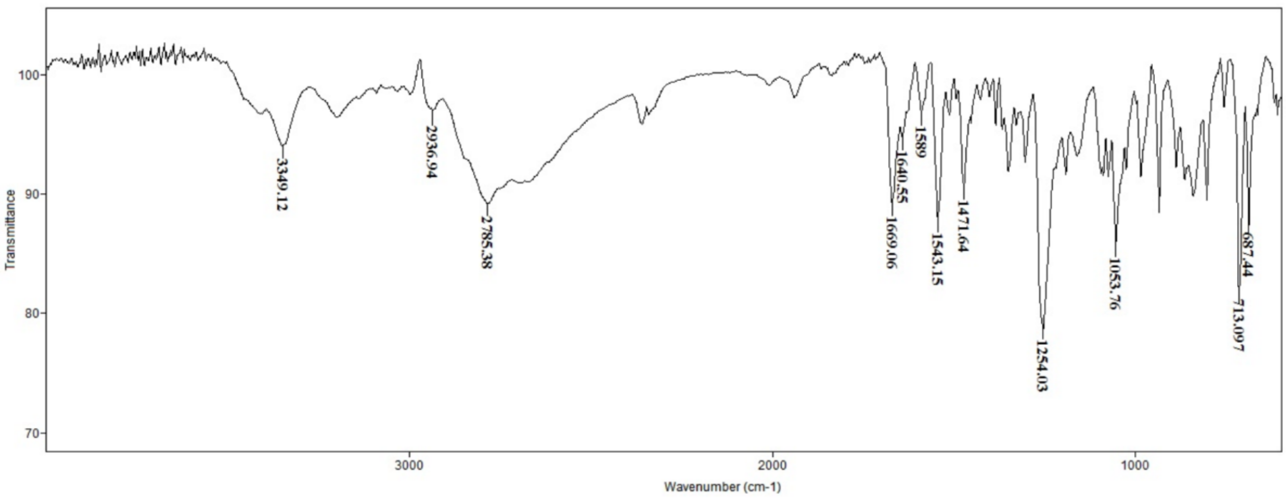

Figure S7. IR spectra of PLBHZ.

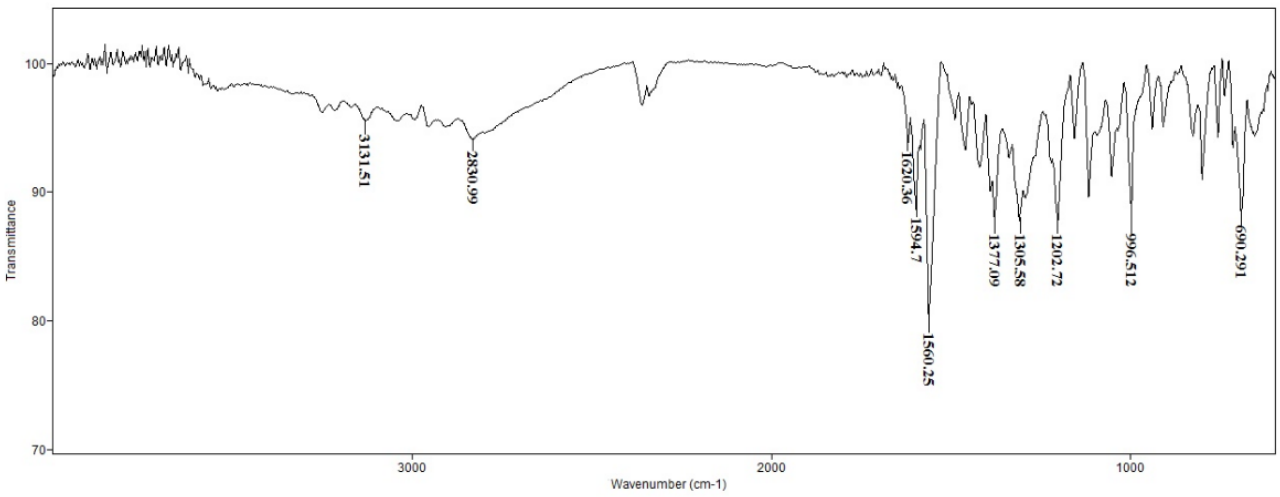

Figure S8. IR spectra of compound (1).

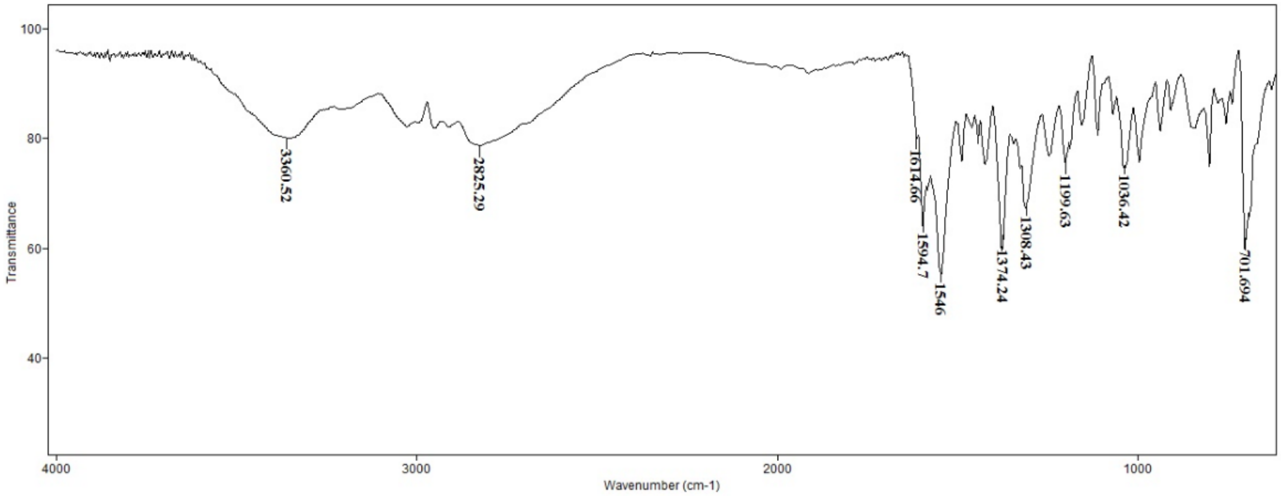

Figure S9. IR spectra of compound (2).

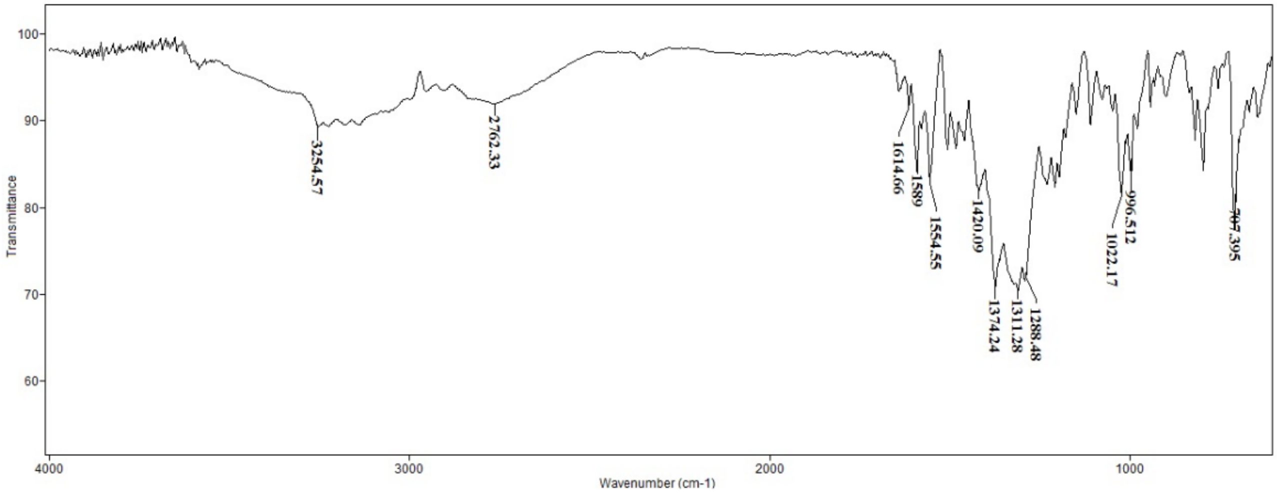

Figure S10. IR spectra of compound (3).

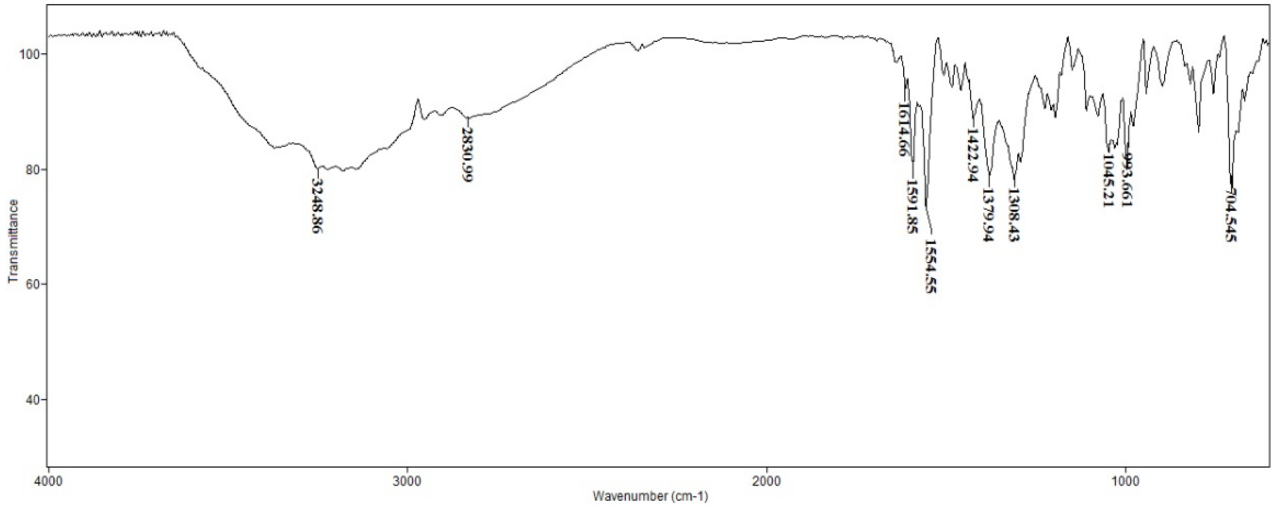

Figure S11. IR spectra of compound (4).

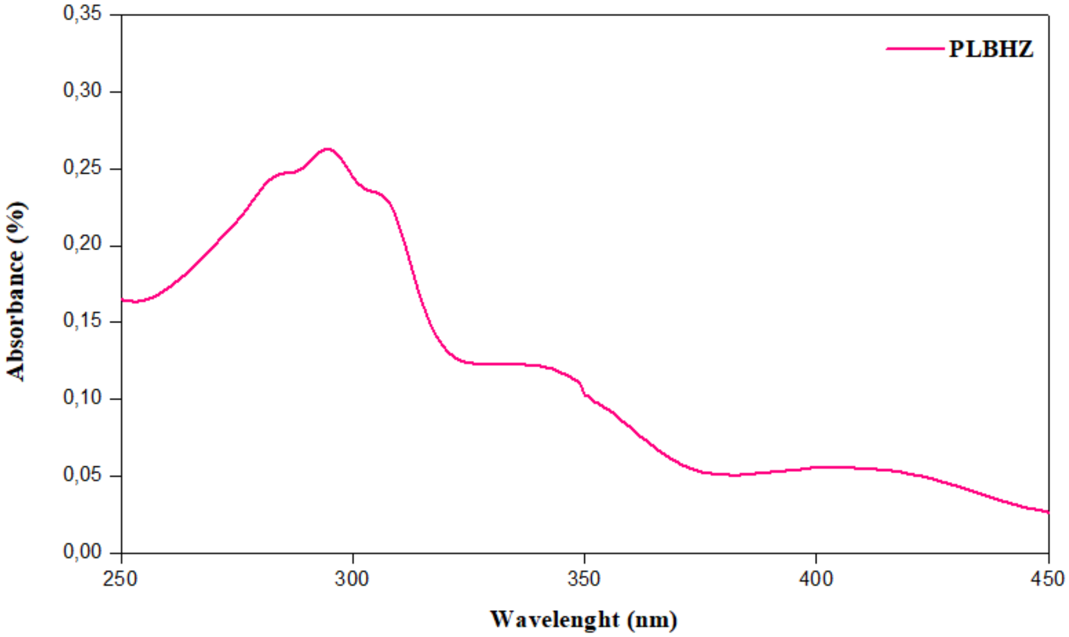

Figure S12. UV-vis spectra of compound PLBHZ in MeOH.

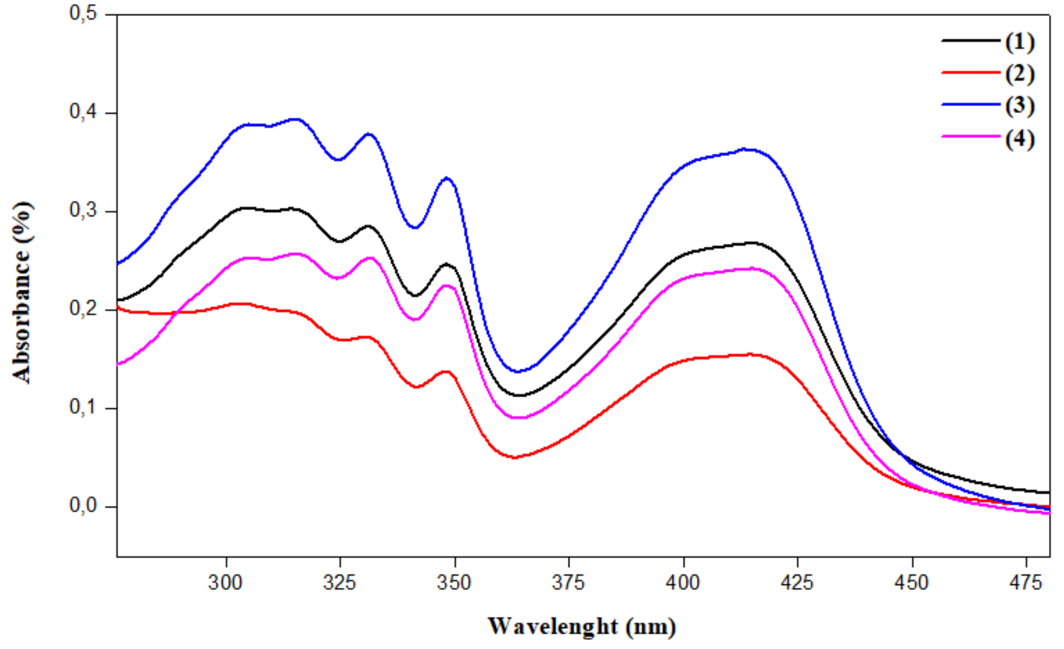

Figure S13. UV-vis spectra of compound (1-4) in MeOH.

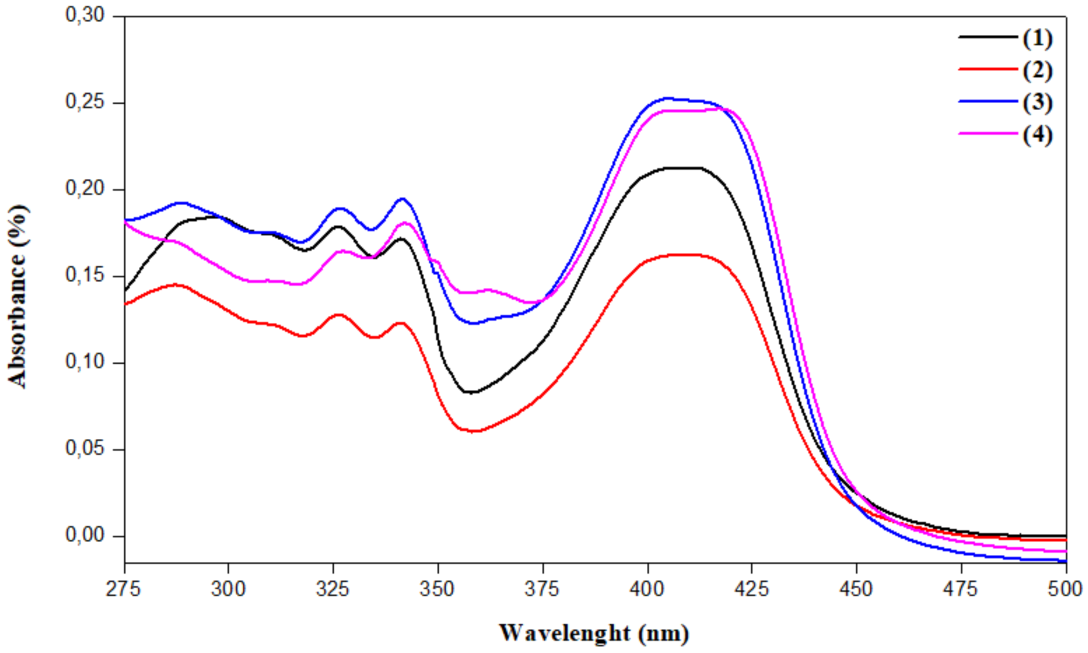

Figure S14. UV-vis spectra of compound (1-4) in DMF.

Table S2. Results obtained in the electron spectroscopy with wavelength values in nm.

Compound	Solvent	$\pi - \pi^{*a}$	Log ε	$n - \pi^{*a}$	Log ε	LMCT [a]	Log ε
**PLBHZ**	MeOH	306	4.11	341	3.84	-	-
**(1)**	MeOH	326	3.95	341	3.98	411	4.04
	DMF	331	4.14	348	4.10	415	4.13
**(2)**	MeOH	326	3.81	341	3.78	410	3.90
	DMF	330	3.93	348	3.84	415	3.90
**(3)**	MeOH	327	3.98	342	3.98	405	4.10
	DMF	331	4.28	348	4.22	413	4.27
**(4)**	MeOH	327	3.90	342	3.95	406	4.10
	DMF	331	4.10	348	4.04	415	4.08

a) Absorption band values in nm.

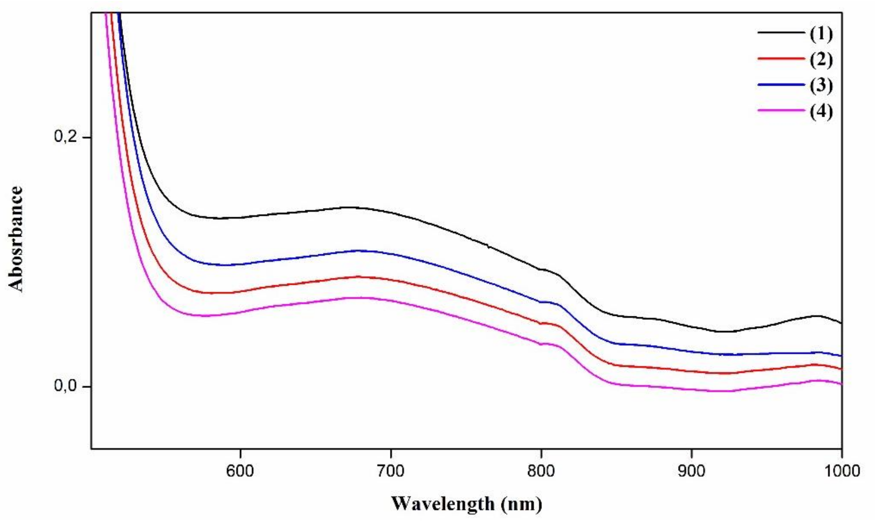

Figure S15. UV-vis spectra of compound (1-4) in DMF (d-d transition).

Table S3. Results obtained in the electron spectroscopy (d-d transition) with wavelength values in nm.

Complex	Solvent	d - d	Log ε
**(1)**	MeOH	672	1.85
**(2)**	MeOH	680	1.64
**(3)**	MeOH	678	1.74
**(4)**	MeOH	680	1.54

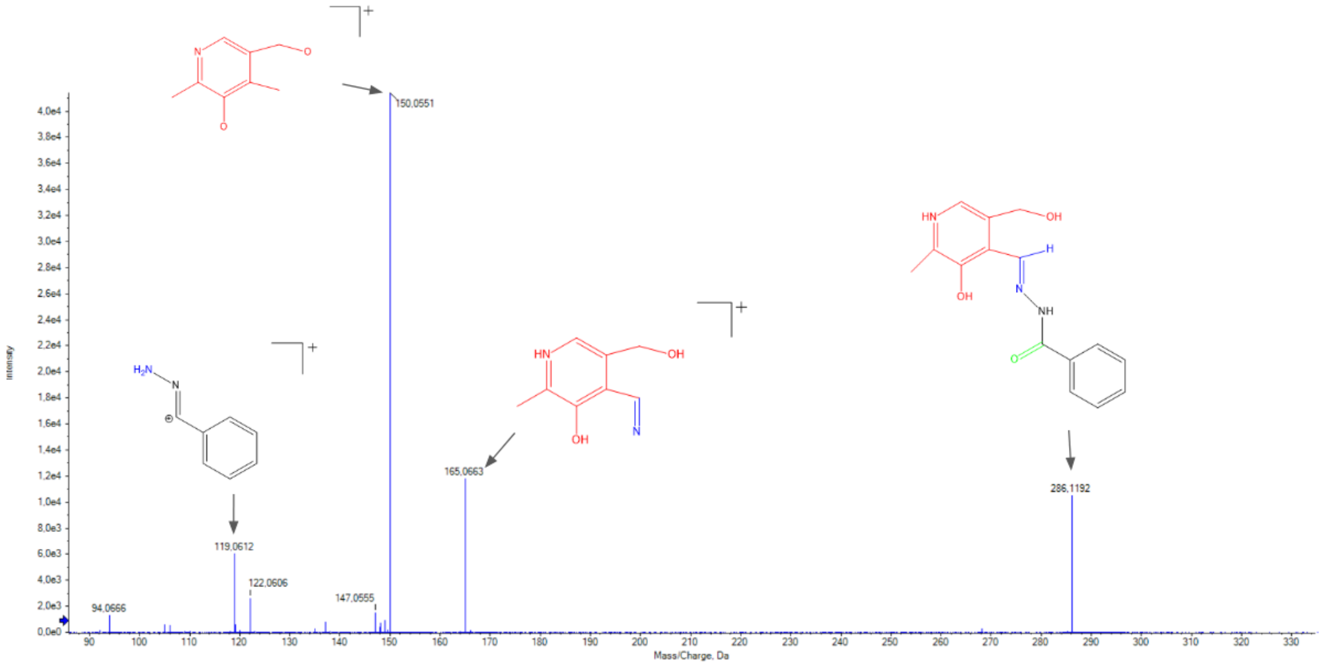

Figure S16. ESI(+)-MSMS of PLBHZ.

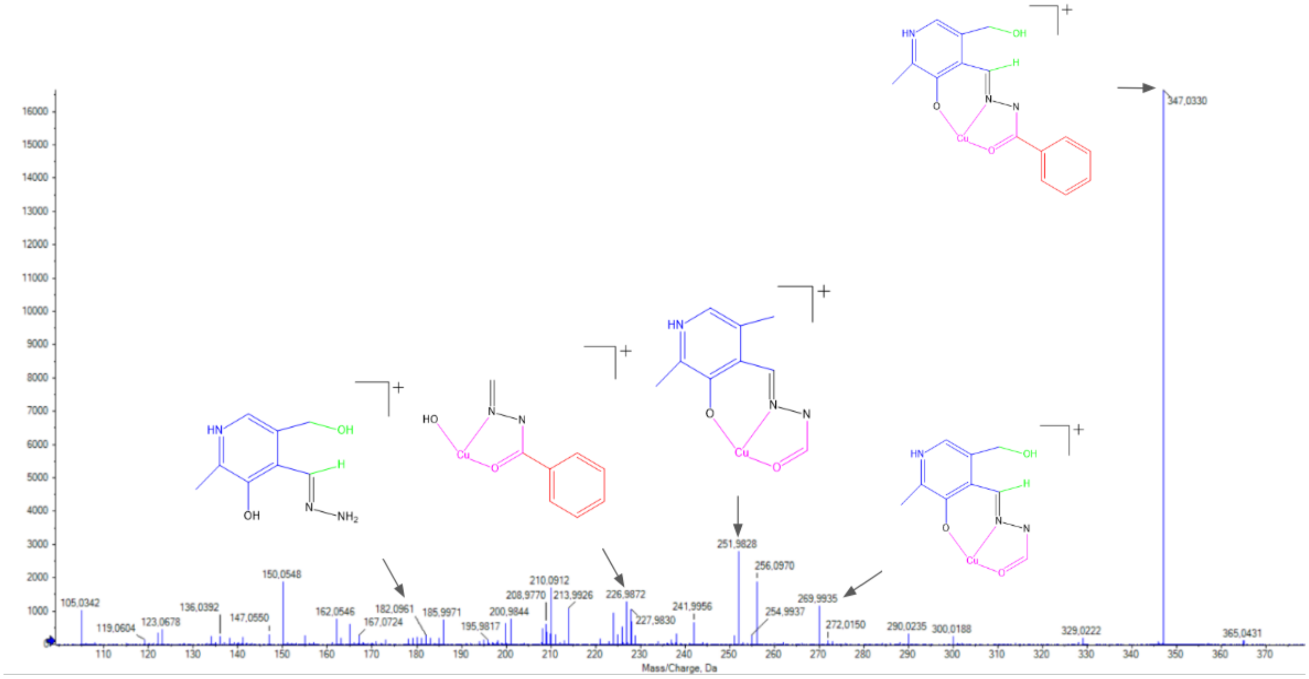

Figure S17. ESI(+)-MSMS of (1).

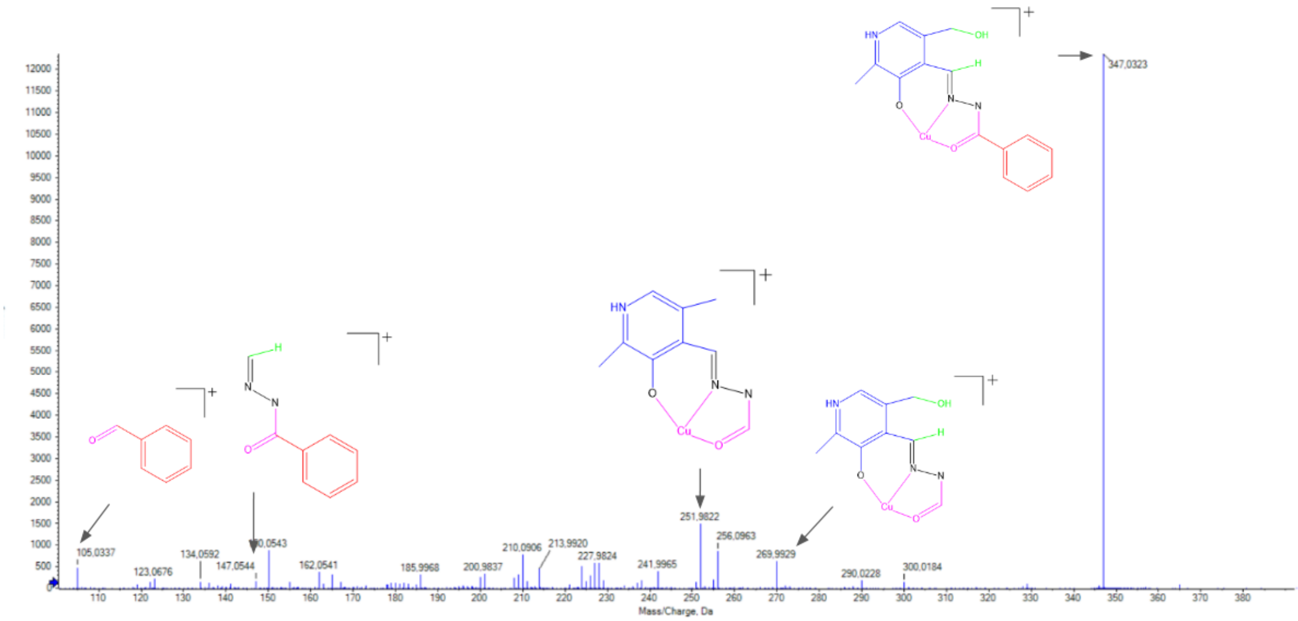

Figure S18. ESI(+)-MSMS of (2).

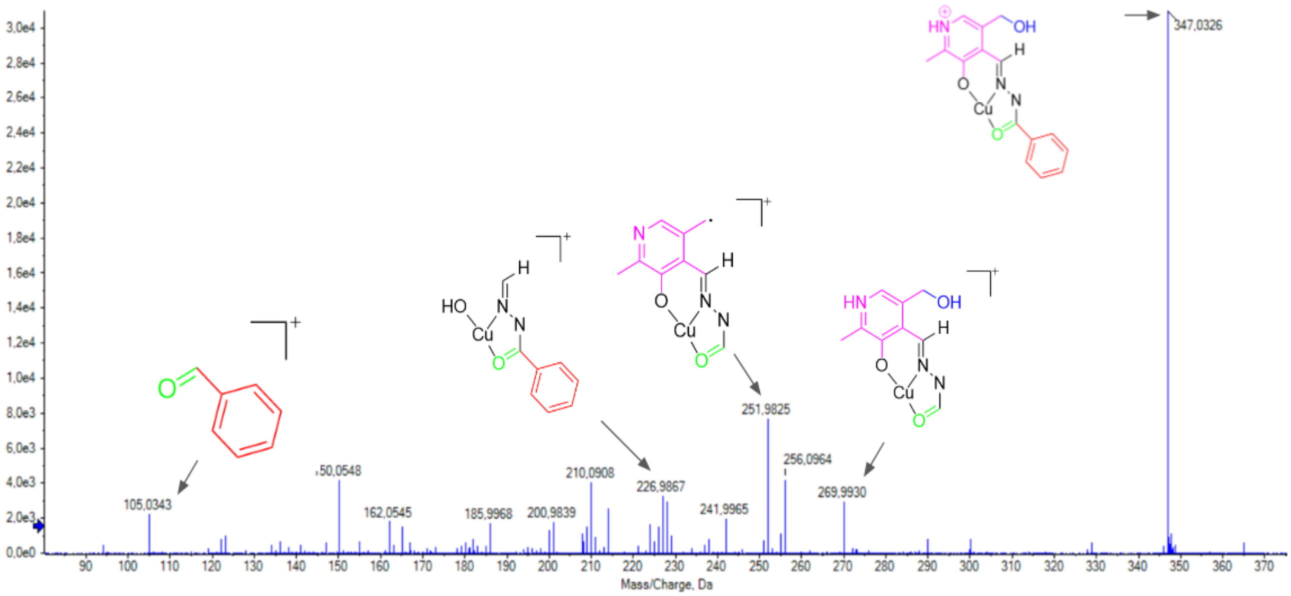

Figure S19. ESI(+)-MSMS of (3).

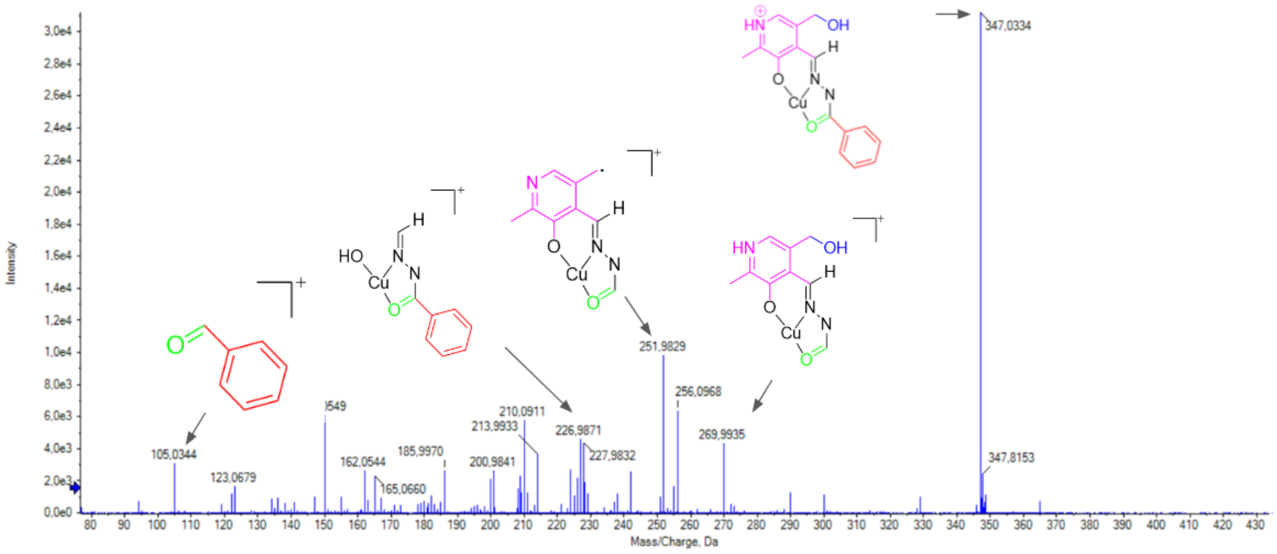

Figure S20. ESI(+)-MSMS of (4).

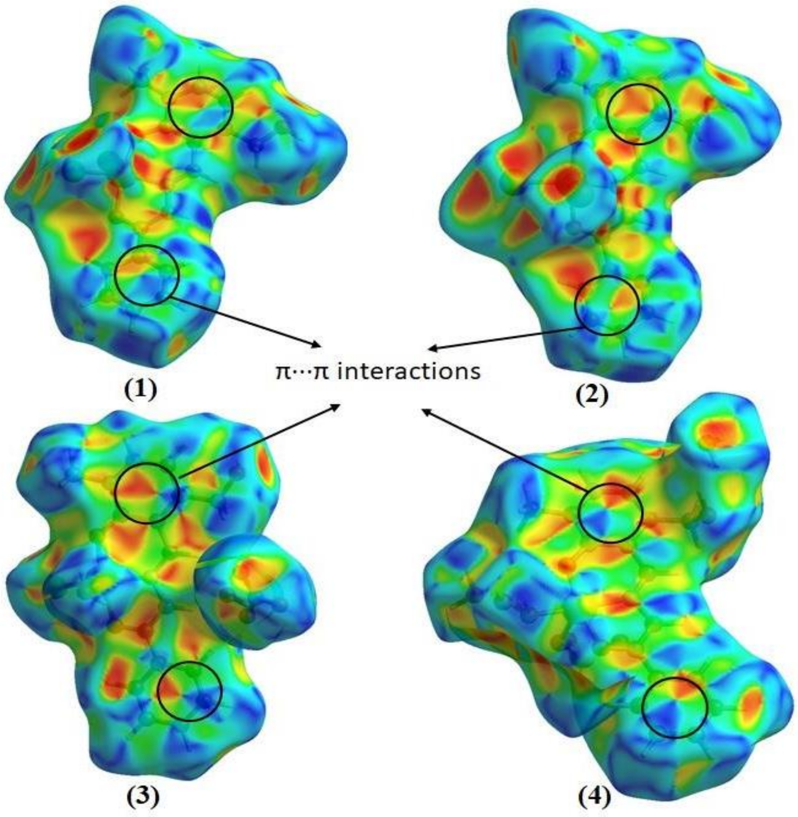

Figure 21. Hirshfeld surface mapped in *shape index* for (1-4).

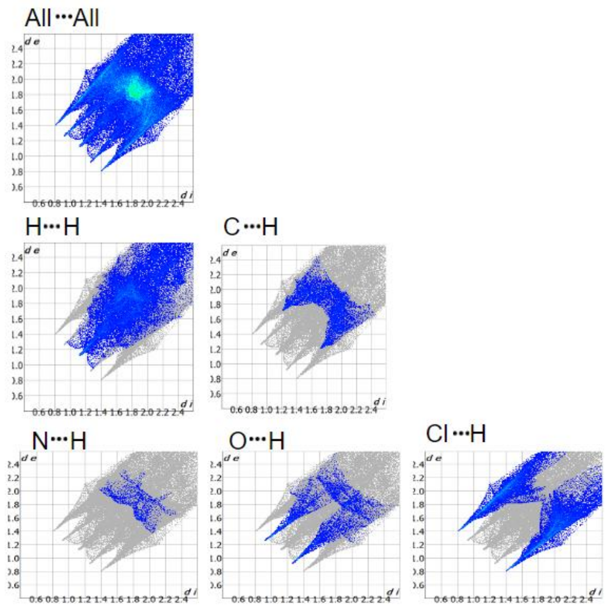

$[CuCl_2(PLBHZ)]$	% contributions
All···All	100
H···H	36.8
C···H	9.4
N···H	2
O··· H	7.7
Cl···H	31.1

Figure S22. Fingerprint plots for (1).

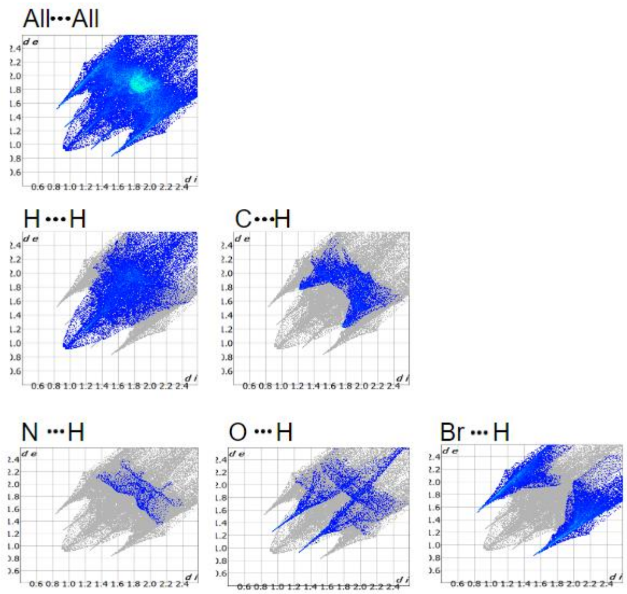

$[CuBr_2(PLBHZ)]$	% contributions
All···All	100
H···H	34.6
C···H	9.9
N···H	2.2
O··· H	7.7
Br··· H	33.1

Figure S23. Fingerprint plots for (2).

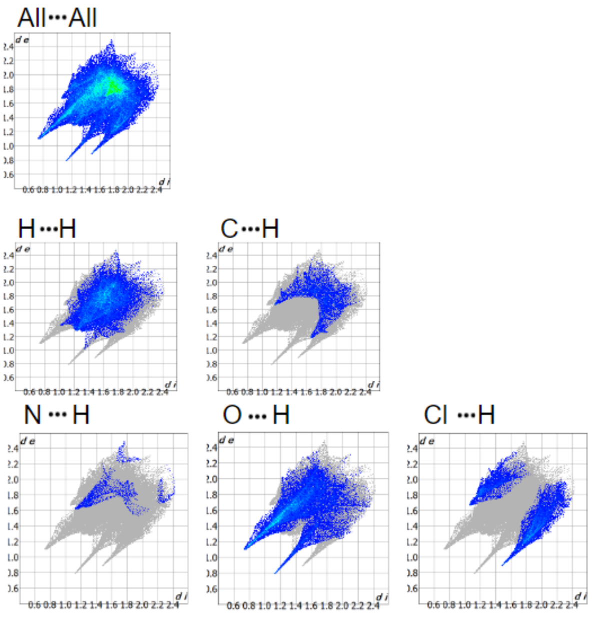

$[CuCl(PLBHZ)H_2O]\cdot NO_3\cdot H_2O$	% contributions
All···All	100
H···H	32.3
C···H	8.3
N···H	1.9
O··· H	27.8
Cl···H	13.1

Figure S24. Fingerprint plots for (3).

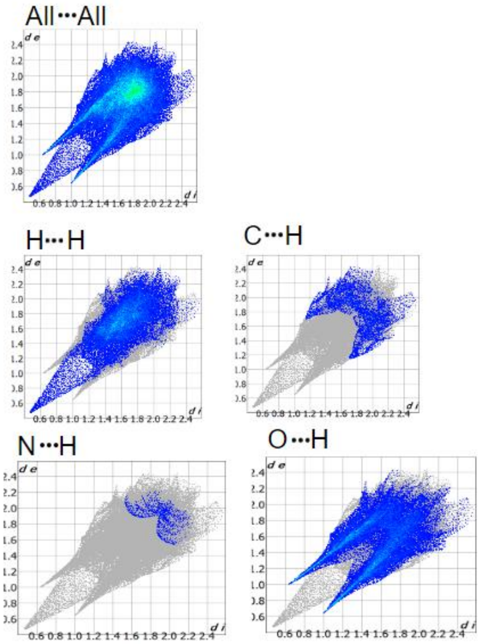

$[CuSO_4(PLBHZ)H_2O]\cdot 3H_2O$	% contributions
All···All	100
H···H	38
C···H	9.2
N···H	1.5
O··· H	39.9

Figure S25. Fingerprint plots for (4).

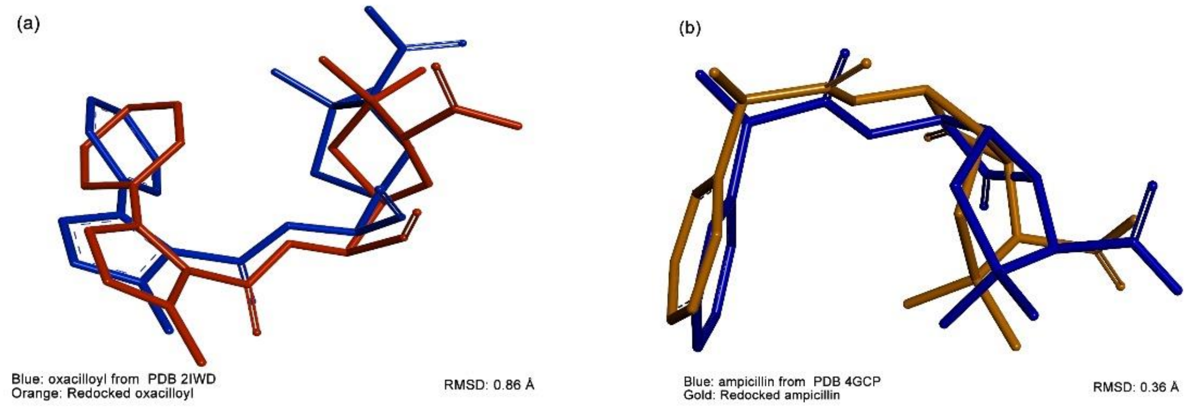

Figure S26. Superposing of best score pose of the redocking study. (a) MecR1-oxalloyl (**PDB** 2IWD). (b) OmpF/A-ampicillin (**PDB** 24GCP).

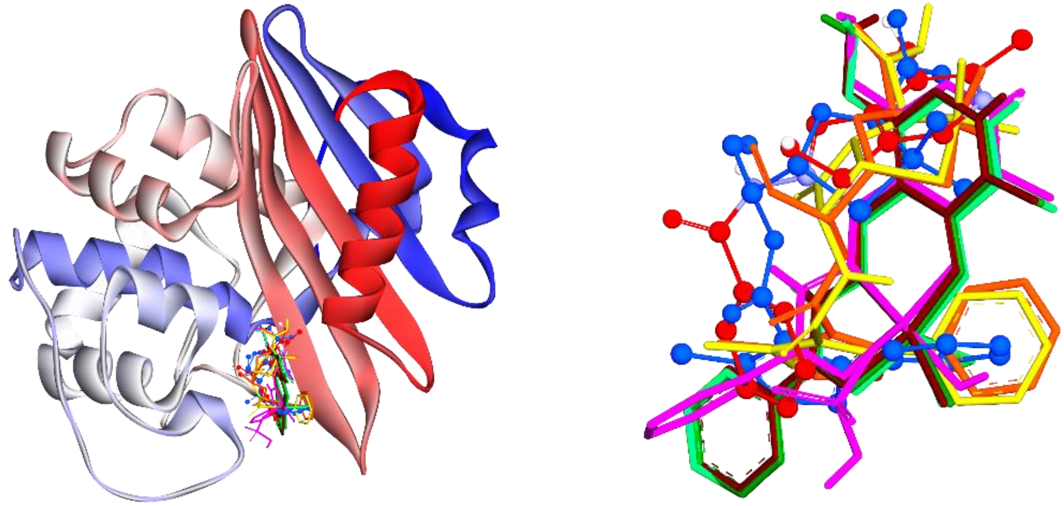

Figure S27. Superposing of best score pose of the docking study of the complex MecR1-ligands (**PDB** 2IWD). Molecule/color: Oxacillin (ball and stick)/blue; Oxacillin/yellow, redocked/orange; PLBHZ (ball and stick)/red; (1)/green; (2)/garnet; (3)/light green; (4)/pink.

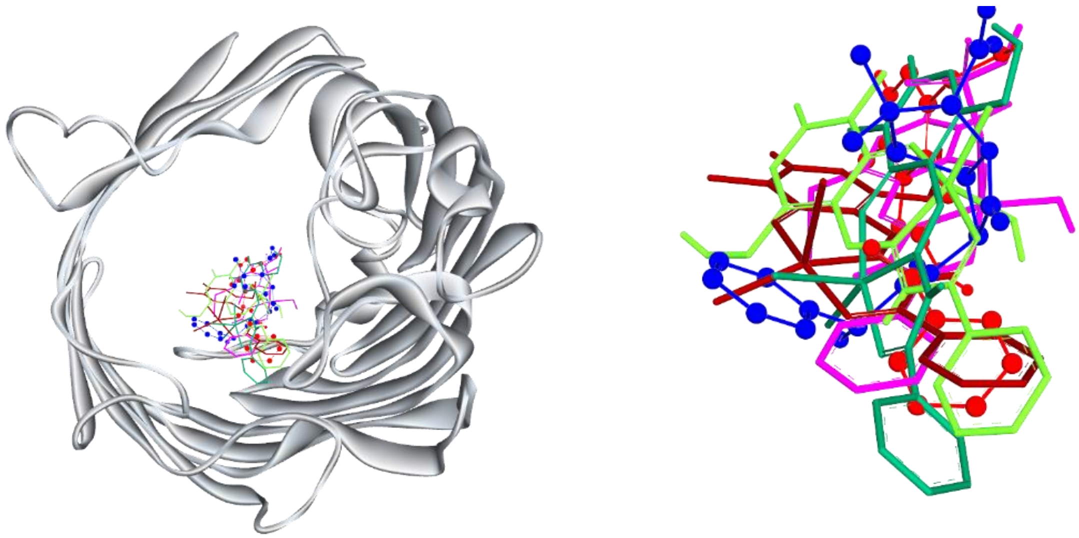

Figure S28. Superposing of best score pose of the docking study of the complex OmpF/A-ligands (**PDB** 4GCP). Molecule/color: ampicillin (ball and stick)/blue; PLBHZ (ball and stick)/red; (1)/green; (2)/garnet; (3)/light green; (4)/pink.
